# Effectiveness of mRNA-1273 vaccination against SARS-CoV-2 omicron subvariants BA.1, BA.2, BA.2.12.1, BA.4, and BA.5

**DOI:** 10.1038/s41467-023-35815-7

**Published:** 2023-01-12

**Authors:** Hung Fu Tseng, Bradley K. Ackerson, Katia J. Bruxvoort, Lina S. Sy, Julia E. Tubert, Gina S. Lee, Jennifer H. Ku, Ana Florea, Yi Luo, Sijia Qiu, Soon Kyu Choi, Harpreet S. Takhar, Michael Aragones, Yamuna D. Paila, Scott Chavers, Carla A. Talarico, Lei Qian

**Affiliations:** 1 Kaiser Permanente Southern California, 100 S. Los Robles Ave., Pasadena, CA 91101 USA; 2 Kaiser Permanente Bernard J. Tyson School of Medicine, 98 S. Los Robles Ave., Pasadena, CA 91101 USA; 3 University of Alabama at Birmingham, 1665 University Blvd, Birmingham, AL 35233 USA; 4 Moderna, Inc., 200 Technology Square, Cambridge, MA 02139 USA; 5 AstraZeneca, 1 Medimmune Way, Gaithersburg, MD 20878 USA

**Keywords:** Viral infection, Epidemiology, SARS-CoV-2, Vaccines

## Abstract

Studies have reported reduced natural SARS-CoV-2 infection- and vaccine-induced neutralization against omicron BA.4/BA.5 compared with earlier omicron subvariants. This test-negative case–control study evaluates mRNA-1273 vaccine effectiveness (VE) against infection and hospitalization with omicron subvariants. The study includes 30,809 SARS-CoV-2 positive and 92,427 SARS-CoV-2 negative individuals aged ≥18 years tested during 1/1/2022-6/30/2022. While 3-dose VE against BA.1 infection is high and wanes slowly, VE against BA.2, BA.2.12.1, BA.4, and BA.5 infection is initially moderate to high (61.0%-90.6% 14-30 days post third dose) and wanes rapidly. The 4-dose VE against infection with BA.2, BA.2.12.1, and BA.4 ranges between 64.3%-75.7%, and is low (30.8%) against BA.5 14-30 days post fourth dose, disappearing beyond 90 days for all subvariants. The 3-dose VE against hospitalization for BA.1, BA.2, and BA.4/BA.5 is 97.5%, 82.0%, and 72.4%, respectively; 4-dose VE against hospitalization for BA.4/BA.5 is 88.5%. Evaluation of the updated bivalent booster is warranted.

## Abstract (web summary)

In this study, the authors estimate the effectiveness of the mRNA-1273 (Moderna) vaccine for Omicron subvariants using data from the USA on ~31,000 cases and ~92,000 matched controls. They find that effectiveness against infection waned rapidly after third and fourth doses, but effectiveness against hospitalization remained high.

## Introduction

Since the detection of SARS-CoV-2 in Wuhan, China, in December 2019, several new variants of concern (VOC) have emerged, many of which were associated with pandemic waves^1^. The most recent VOC, omicron, first detected in South Africa in November 2021, is substantially more transmissible than earlier VOCs^2^ and contains multiple mutations that confer greater escape from naturally acquired and vaccine-elicited immunity compared with earlier variants^3,4^. Together, these characteristics allowed omicron to rapidly become the dominant strain globally and resulted in large waves of infection much greater than any seen previously during the pandemic^2,5,6^. Within a few months after the emergence of omicron, the initially dominant subvariant BA.1 was replaced by BA.2 and BA.2.12.1 subvariants, which are more transmissible^5,7,8^ but do not appear to have a greater ability to evade vaccine-elicited protection than BA.1^9^. However, soon after, the subvariants BA.4 and BA.5 became the dominant strains globally^10–12^. Several in vitro studies reported lower natural SARS-CoV-2 infection- and vaccine-induced neutralization activity against BA.4 and BA.5 than earlier omicron subvariants, raising concerns about potentially increased escape from natural and vaccine-induced protection^11,13–20^.

Previous studies have shown markedly reduced vaccine effectiveness (VE) of two doses of mRNA vaccines, including mRNA-1273 (Spikevax; Moderna Inc, Cambridge, MA, USA) and BNT162b2 (Cominarty; Pfizer Inc, New York, NY, USA; BioNTech Manufacturing GmbH, Mainz, Germany), against infection with BA.1 compared with earlier VOCs^21^. After a third dose, VE of mRNA vaccines initially improved but waned quickly^9,21–25^. We previously found that the two-dose VE of mRNA-1273 against BA.1 infection was initially 44.0% compared with 80.2% against delta infection, waning to 5.9% and 61.3% at >270 days, for BA.1 and delta, respectively^22^. Similarly, three-dose VE of mRNA-1273 against BA.1 infection decreased from 72.1% to 51.2% at >60 days, while three-dose VE against delta infection only declined from 94.2% to 88.1% over the same time interval^22^. Additional studies of mRNA vaccines found that two-dose VE against hospitalization with BA.1 was modest and waned quickly^23–25^, and while three-dose VE against hospitalization with BA.1 was initially higher, it also waned^23–28^. Of concern, the substantially greater ability of BA.4 and BA.5 to escape vaccine-elicited immunity compared with BA.1 suggests that there may be even greater declines in VE of current vaccines against the BA.4 and BA.5 subvariants.

Few studies have examined the effectiveness of mRNA vaccines against emerging omicron subvariants; this research is critical to inform decisions around the need for variant-specific boosters that may offer broader protection against omicron subvariants. As such, we conducted a test-negative case-control study in the Kaiser Permanente Southern California (KPSC) healthcare system in the United States to evaluate the effectiveness of monovalent mRNA-1273 against infection with and COVID-19 hospitalization for omicron subvariants, including BA.4 and BA.5.

## Results

**Fig. 1:**
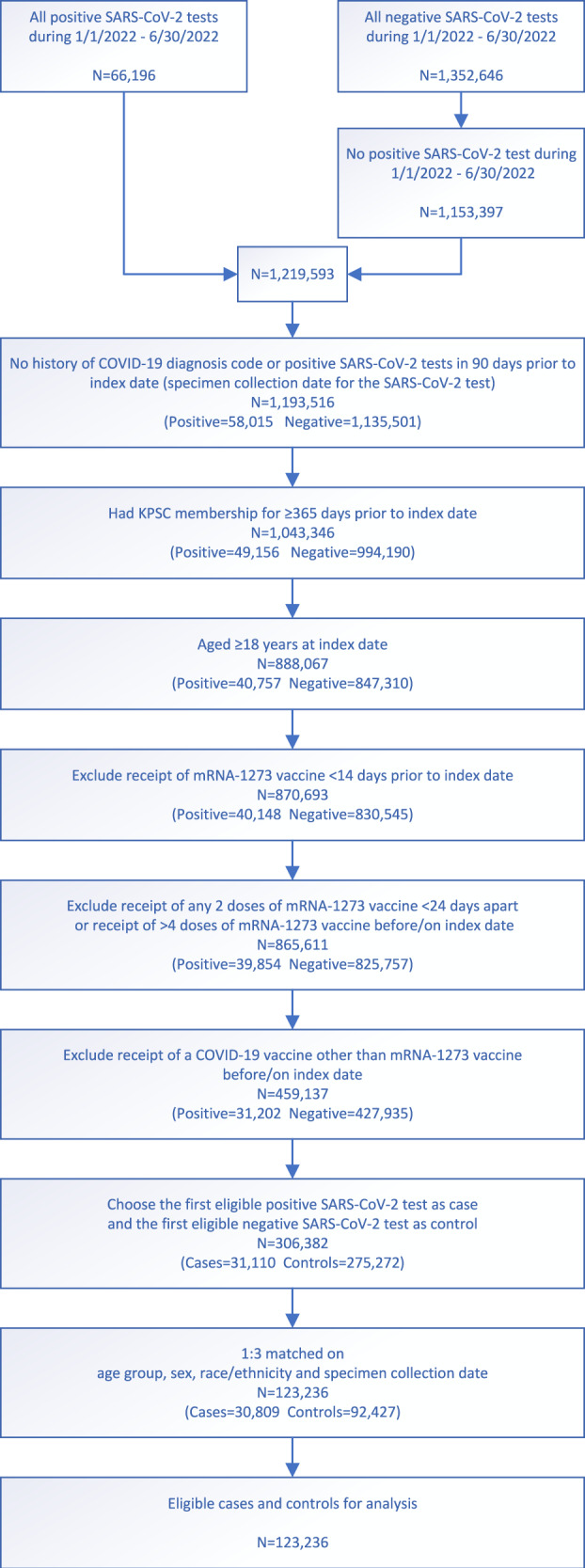
Flow chart for mRNA-1273 vaccine test-negative design. (*N* = 123,236). Steps for selection of 30,809 cases and 92,427 controls by inclusion and exclusion criteria, and subsequent matching for vaccine effectiveness estimation. COVID-19 coronavirus disease; KPSC Kaiser Permanente Southern California.

We describe the flow of case and control selection in Fig. 1. A total of 123,236 individuals (30,809 test-positive cases and 92,427 test-negative controls) were included in the study. Of the 30,809 cases, 16,418 (53.3%) were successfully sequenced, 93.2% of which had a composite Ct value ≤27, compared to only 13.1% of the failed sequencing samples (Supplementary Table 1). We present the distribution of SARS-CoV-2 variants by mRNA-1273 vaccination status and by month of specimen collection in Supplementary Fig. 1 and Supplementary Fig. 2, respectively. Overall, BA.1 circulated between January and April 2022; BA.2 (excluding BA.2.12.1) appeared at the end of January 2022 and BA.2.12.1 appeared in late March 2022; both subvariants continued to circulate through the remainder of the study period. BA.4 and BA.5 appeared in early May 2022, and the proportion attributed to these subvariants, especially BA.5, rapidly increased in June 2022.

We described baseline characteristics of cases and controls in Table 1. The median age of individuals included in the study was 46 years, of which 18% were aged ≥65 years. Females accounted for 55.7% of the total population. Forty-five percent of the individuals were Hispanic, 28.9% non-Hispanic White, 8.1% non-Hispanic Black, 11.4% non-Hispanic Asian, and 6.6% other or unknown race/ethnicity. Overall, 17.6% of specimens were saliva (used for testing asymptomatic individuals). Cases and controls had a similar distribution of many covariates (absolute standardized difference [ASD] ≤ 0.1), including body mass index, smoking history, Charlson comorbidity score, frailty index, prevalence of chronic diseases, pregnancy status, immunocompromised status, prevalence of autoimmune conditions, history of emergency department (ED) visits, history of hospitalization, use of preventive care, Medicaid status, neighborhood median household income, KSPC physician/employee status, and specimen type.

**Table 1:** Characteristics of SARS-CoV-2 test-positive cases and test-negative controls

	Test positive	Test negative	*p* value	ASD
	N = 30809	*N* = 92427		
Vaccination status, *n* (%)			<0.01	0.16
1-dose	278 (0.9%)	877 (0.9%)		
2-dose	6932 (22.5%)	18914 (20.5%)		
3-dose	12724 (41.3%)	43995 (47.6%)		
4-dose	966 (3.1%)	4084 (4.4%)		
Unvaccinated	9909 (32.2%)	24557 (26.6%)		
Age at specimen collection date, years			0.19	0.01
Mean (sd)	47.63 (17.07)	47.76 (17.32)		
Median	46	46		
Q1, Q3	34, 60	34, 61		
Min, max	18, 105	18, 107		
Age at specimen collection date, years, *n* (%)			N/A	N/A
18–44	14536 (47.2%)	43608 (47.2%)		
45–64	10708 (34.8%)	32124 (34.8%)		
65–74	3271 (10.6%)	9813 (10.6%)		
≥75	2294 (7.4%)	6882 (7.4%)		
Sex, *n* (%)			N/A	N/A
Female	17167 (55.7%)	51501 (55.7%)		
Male	13642 (44.3%)	40926 (44.3%)		
Race/Ethnicity, *n* (%)			N/A	N/A
Non-Hispanic White	8903 (28.9%)	26709 (28.9%)		
Non-Hispanic Black	2503 (8.1%)	7509 (8.1%)		
Hispanic	13869 (45.0%)	41607 (45.0%)		
Non-Hispanic Asian	3499 (11.4%)	10497 (11.4%)		
Other/unknown	2035 (6.6%)	6105 (6.6%)		
Body mass index^a^, kg/m^2^, *n* (%)			<0.01	0.06
<18.5	303 (1.0%)	987 (1.1%)		
18.5–<25	6140 (19.9%)	18913 (20.5%)		
25–<30	8696 (28.2%)	26804 (29.0%)		
30–<35	6351 (20.6%)	18828 (20.4%)		
35–<40	3122 (10.1%)	9734 (10.5%)		
40–<45	1366 (4.4%)	4253 (4.6%)		
≥45	933 (3.0%)	2857 (3.1%)		
Unknown	3898 (12.7%)	10051 (10.9%)		
Smoking^a^, *n* (%)			<0.01	0.06
No	22543 (73.2%)	67110 (72.6%)		
Yes	5206 (16.9%)	17359 (18.8%)		
Unknown	3060 (9.9%)	7958 (8.6%)		
Charlson comorbidity score^b,c^			<0.01	0.06
Mean (sd)	0.73 (1.59)	0.83 (1.69)		
Median	0	0		
Q1, Q3	0, 1	0, 1		
Min, Max	0, 18	0, 15		
Charlson comorbidity score^b,c^ *n* (%)			<0.01	0.07
0	21590 (70.1%)	61993 (67.1%)		
1	4442 (14.4%)	13821 (15.0%)		
≥2	4777 (15.5%)	16613 (18.0%)		
Frailty index^b,d^			<0.01	0.06
Mean (sd)	0.12 (0.03)	0.12 (0.03)		
Median	0.11	0.11		
Q1, Q3	0.10, 0.13	0.10, 0.13		
Min, max	0.05, 0.40	0.05, 0.42		
Frailty index^b,d^, *n* (%)			<0.01	0.08
Quartile 1	7169 (23.3%)	21404 (23.2%)		
Quartile 2	8926 (29.0%)	24117 (26.1%)		
Quartile 3	7666 (24.9%)	23147 (25.0%)		
Quartile 4, most frail	7048 (22.9%)	23759 (25.7%)		
Chronic diseases^b^, *n* (%)				
Kidney disease	1623 (5.3%)	5549 (6.0%)	<0.01	0.03
Heart disease	1069 (3.5%)	3632 (3.9%)	<0.01	0.02
Lung disease	2842 (9.2%)	9495 (10.3%)	<0.01	0.04
Liver disease	1155 (3.7%)	4137 (4.5%)	<0.01	0.04
Diabetes	4313 (14.0%)	13766 (14.9%)	<0.01	0.03
Immunocompromised, *n* (%)	1165 (3.8%)	4326 (4.7%)	<0.01	0.04
HIV/AIDS, *n*	78	365		
Leukemia/lymphoma, congenital and other immunodeficiencies, asplenia/hyposplenia, *n*	425	1443		
Hematopoietic stem cell transplant/organ transplant, *n*	145	459		
Immunosuppressant medications, *n*	785	2906		
Autoimmune conditionsb, *n* (%)	922 (3.0%)	3110 (3.4%)	<0.01	0.02
Rheumatoid arthritis, *n*	388	1340		
Inflammatory bowel disease, *n*	194	705		
Psoriasis and psoriatic arthritis, *n*	326	973		
Multiple sclerosis, *n*	54	165		
Systemic lupus erythematosus, *n*	79	317		
Pregnant at specimen collection date, *n* (%)	742 (2.4%)	3954 (4.3%)	<0.01	0.10
1st trimester, *n*	126	370		
2nd trimester, *n*	216	533		
3rd trimester, *n*	400	3051		
History of SARS-CoV-2 infection, *n* (%)	4512 (14.6%)	19811 (21.4%)	<0.01	0.18
History of SARS-CoV-2 molecular teste, *n* (%)	23802 (77.3%)	64378 (69.7%)	<0.01	0.17
Number of outpatient and virtual visits^b^, *n* (%)			<0.01	0.15
0	2157 (7.0%)	5649 (6.1%)		
1–4	8192 (26.6%)	19958 (21.6%)		
5–10	8951 (29.1%)	26395 (28.6%)		
≥11	11509 (37.4%)	40425 (43.7%)		
Number of Emergency Department visits^b^, *n* (%)			<0.01	0.07
0	24801 (80.5%)	71843 (77.7%)		
1	3920 (12.7%)	13648 (14.8%)		
≥2	2088 (6.8%)	6936 (7.5%)		
Number of hospitalizationsb, n (%)			<0.01	0.03
0	29016 (94.2%)	86543 (93.6%)		
1	1391 (4.5%)	4437 (4.8%)		
≥2	402 (1.3%)	1447 (1.6%)		
Preventive careb, *n* (%)	21899 (71.1%)	69064 (74.7%)	<0.01	0.08
Medicaid, *n* (%)	2941 (9.5%)	10057 (10.9%)	<0.01	0.04
Neighborhood median household income, *n* (%)			0.02	0.02
<$40,000	1099 (3.6%)	3545 (3.8%)		
$40,000–$59,999	5657 (18.4%)	17505 (18.9%)		
$60,000–$79,999	7710 (25.0%)	22652 (24.5%)		
$80,000+	16324 (53.0%)	48665 (52.7%)		
Unknown	19 (0.1%)	60 (0.1%)		
KPSC physician/employee, *n* (%)	1755 (5.7%)	3664 (4.0%)	<0.01	0.08
Medical center area^f^			<0.01	0.15
Month of specimen collection, *n* (%)			<0.01	0.23
January 2022	6797 (22.1%)	23700 (25.6%)		
February 2022	4602 (14.9%)	9108 (9.9%)		
March 2022	1311 (4.3%)	6199 (6.7%)		
April 2022	2613 (8.5%)	11177 (12.1%)		
May 2022	7828 (25.4%)	21047 (22.8%)		
June 2022	7658 (24.9%)	21196 (22.9%)		
Specimen type, *n* (%)			0.85	<0.01
Nasopharyngeal/oropharyngeal swab	25387 (82.4%)	76116 (82.4%)		
Saliva	5422 (17.6%)	16311 (17.6%)		

^a^Defined in the 2 years prior to specimen collection date.

^b^Defined in the 1 year prior to specimen collection date.

^c^Possible range: 0–29^43^.

^d^Possible range: 0–1^44^.

^e^Defined based on all available medical records from 1 March 2020 to specimen collection date.

^f^Frequency and percent for the 19 medical center areas are not shown.

*χ*^2^ tests were used for categorical variables and two-sided, two-sample *t* tests were used for continuous variables. No adjustments were made for multiple comparisons. *ASD* absolute standardized difference, *N/A* not applicable.

**Fig. 2:**
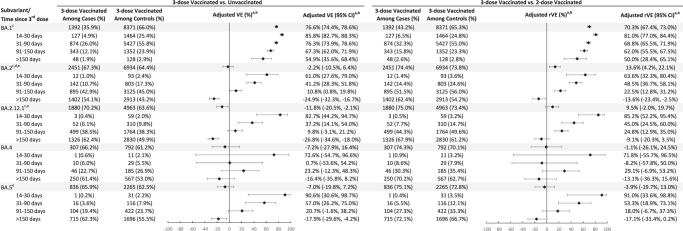
Adjusted vaccine effectiveness (VE) and relative vaccine effectiveness (rVE) of mRNA-1273 against infection with omicron subvariants by time since 3rd dose vaccination. Adjusted VE of three doses of mRNA-1273 and adjusted rVE of three versus two doses of mRNA-1273 and their 95% confidence intervals against infection with SARS-CoV-2 omicron subvariants. Sample size for each analysis equals the number of three-dose vaccinated divided by the percentage of three-dose vaccinated. Symbols represent the adjusted vaccine effectiveness or adjusted relative vaccine effectiveness. ^a^When the OR or its 95% CI was >1, the VE/rVE or its 95% CI was transformed as ([1/OR] – 1) × 100. ^b^Adjusted for age, sex, race/ethnicity, month of specimen collection, history of SARS-CoV-2 infection, history of SARS-CoV-2 molecular test, number of outpatient and virtual visits, medical center area, and time between second dose and specimen collection date (for three-dose versus two-dose models only). ^c^Medical center area removed from adjustment set in three-dose versus two-dose models due to lack of model convergence. ^d^BA.2 excluding BA.2.12.1. ^e^Medical center area removed from adjustment set in three-dose versus unvaccinated models due to lack of model convergence. CI confidence interval, OR odds ratio, rVE relative vaccine effectiveness, VE vaccine effectiveness.

In analyses of three-dose VE (versus unvaccinated) against infection with omicron subvariants by time since vaccination, the three-dose VE against BA.1 ranged from 85.8% (95% confidence interval [CI] 82.7%, 88.3%) in the 14–30 days after the third dose to 54.9% (95% CI 35.6%, 68.4%) >150 days after the third dose (Fig. 2, Supplementary Table 2a). VE for these two time intervals, respectively, was 61.0% (95% CI 27.6%, 79.0%) and −24.9% (95% CI −32.3%, −16.7%) for BA.2, excluding BA.2.12.1; 82.7% (95% CI 44.2%, 94.7%) and −26.8% (95% CI −34.6%, −18.0%) for BA.2.12.1; 72.6% (95% CI −54.7%, 96.6%) and −16.4% (95% CI −35.8%, 8.2%) for BA.4; and 90.6% (95% CI 30.6%, 98.7%) and −17.9% (95% CI −29.6%, −4.2%) for BA.5. We also present the relative VE (rVE) comparing three doses to two doses against omicron subvariants by time since vaccination (Fig. 2, Supplementary Table 2b). In general, we observed consistent incremental protection of three doses versus two doses in the 14–90 days after the third dose, other than against BA.4, which had a small number of cases and wide CI. The incremental benefit in protection decreased over time since the third dose. For BA.5, the 95% CI of rVE included 0 after >90 days after the third dose.

**Fig. 3:**
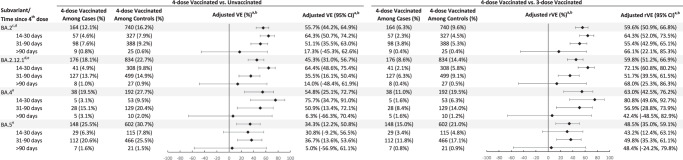
Adjusted vaccine effectiveness (VE) and relative vaccine effectiveness (rVE) of mRNA-1273 against infection with omicron subvariants by time since 4th dose vaccination. Adjusted VE of four doses of mRNA-1273 and adjusted rVE of four versus three doses of mRNA-1273 and their 95% confidence intervals against infection with SARS-CoV-2 omicron subvariants. Sample size for each analysis equals the number of four-dose vaccinated divided by the percentage of four-dose vaccinated. Symbols represent the adjusted vaccine effectiveness or adjusted relative vaccine effectiveness. ^a^When the OR or its 95% CI was >1, the VE/rVE or its 95% CI was transformed as ([1/OR] – 1) × 100. ^b^Adjusted for age, sex, race/ethnicity, month of specimen collection, history of SARS-CoV-2 infection, history of SARS-CoV-2 molecular test, number of outpatient and virtual visits, medical center area, and time between third dose and specimen collection date (for four-dose versus three-dose models only). ^c^BA.2 excluding BA.2.12.1. ^d^Medical center area removed from adjustment set in four-dose versus three-dose models due to lack of model convergence. ^e^Medical center area removed from adjustment set in four-dose versus unvaccinated models due to lack of model convergence. CI confidence interval, OR odds ratio, rVE relative vaccine effectiveness, VE vaccine effectiveness.

In analyses of four-dose VE against infection with omicron subvariants by time since vaccination, because a fourth dose (second booster) in adults ages ≥50 years was not recommended until the tail-end of the BA.1 period, there were insufficient numbers to estimate four-dose VE against BA.1. The four-dose VE against BA.2 was 64.3% (95% CI 50.7%, 74.2%) 14–30 days after the fourth dose and 17.3% (95% CI −45.3%, 62.6%) >90 days after the fourth dose (Fig. 3, Supplementary Table 3a). VE for these time intervals, respectively, was 64.4% (95% CI 48.6%, 75.4%) and 14.0% (95% CI −48.4%, 61.9%) for BA.2.12.1; 75.7% (95% CI 34.7%, 91.0%) and 6.3% (95% CI −66.3%, 70.4%) for BA.4; and 30.8% (95% CI −9.2%, 56.5%) and 5.0% (95% CI −56.9%, 61.1%) for BA.5. We also present the rVE comparing four doses to three doses against omicron subvariants by time since vaccination (Fig. 3, Supplementary Table 3b). We observed consistent incremental protection of four doses compared with three doses in the 14–90 days after the fourth dose. For BA.4 and BA.5, the 95% CI of rVE included 0 after >90 days after the fourth dose.

**Fig. 4:**
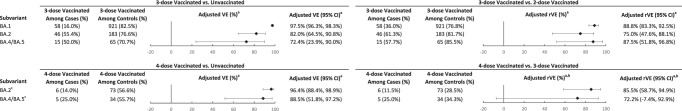
Adjusted vaccine effectiveness (VE) and relative vaccine effectiveness (rVE) of mRNA-1273 against hospitalization for omicron subvariants by time since 3rd or 4th dose vaccination. Adjusted VE of 3 and four doses of mRNA-1273 and adjusted rVE of 3 versus two doses and 4 versus three doses of mRNA-1273 and their 95% confidence intervals against hospitalization for SARS-CoV-2 omicron subvariants. Sample size for each analysis equals the number of three-dose vaccinated divided by the percentage of three-dose vaccinated for the three-dose models, and is equal to the number of four-dose vaccinated divided by the percentage of four-dose vaccinated for the four-dose models. ^a^Adjusted for age, sex, race/ethnicity, month of specimen collection, history of SARS-CoV-2 infection, history of SARS-CoV-2 molecular test, number of outpatient and virtual visits, and time between second/third dose and specimen collection date (for three-dose versus two-dose and four-dose versus three-dose models, respectively). Medical center area dropped from adjustment set due to lack of model convergence. Symbols represent the adjusted vaccine effectiveness or adjusted relative vaccine effectiveness. ^b^When the OR or its 95% CI was >1, the VE/rVE or its 95% CI was transformed as ([1/OR] – 1) × 100. ^c^History of SARS-CoV-2 infection dropped from adjustment set in four-dose versus three-dose models due to lack of model convergence. CI confidence interval, OR odds ratio, rVE relative vaccine effectiveness, VE vaccine effectiveness.

We examined three-dose VE against COVID-19 hospitalization for BA.1, BA.2 (including BA.2.12.1), and BA.4/BA.5. The three-dose VE against hospitalization for BA.1 was 97.5% (95% CI 96.3%, 98.3%) (Fig. 4, Supplementary Table 4a). The three-dose VE against hospitalization for BA.2 was 82.0% (95% CI 64.5%, 90.8%), while three-dose VE against hospitalization for BA.4/BA.5 was 72.4% (95% CI 23.9%, 90.0%). We also present the rVE comparing three doses to two doses against hospitalization for BA.1, BA.2, and BA.4/BA.5 (Fig. 4, Supplementary Table 4b). The rVE against these omicron subvariants, respectively, was 88.8% (95% CI 83.3%, 92.5%), 75.0% (95% CI 47.6%, 88.1%), and 87.5% (95% CI 51.8%, 96.8%).

In the analyses of four-dose VE against COVID-19 hospitalization for BA.2 (including BA.2.12.1) and BA.4/BA.5, the four-dose VE against hospitalization for BA.2 was 96.4% (95% CI 88.4%, 98.9%) (Fig. 4, Supplementary Table 5a). The four-dose VE against hospitalization for BA.4/BA.5 was 88.5% (95% CI 51.8%, 97.2%). We also present the rVE comparing four doses to three doses against hospitalization for BA.2 and BA.4/BA.5 (Fig. 4, Supplementary Table 5b). The rVE against these omicron subvariants, respectively, was 85.5% (95% CI 58.7%, 94.9%) and 72.2% (95% CI −7.4%, 92.9%).

In the sensitivity analyses, we imputed omicron subvariant information using available S-gene target failure (SGTF) data for 12,006 specimens (83.4%) that were not successfully sequenced. By comparing whole genome sequencing (WGS) results and available SGTF results, the positive predictive value of using SGTF results combined with calendar month to predict omicron subvariants BA.1, BA.2, and BA.4/BA.5 was 99.9%, 99.1%, and 96.1%, respectively. The VE and rVE results are presented in Supplementary Tables 6a–9b. In general, the increased sample size allowed for more precise estimation of VE and rVE against infection and hospitalization, as shown in narrower CIs. The VE and rVE against infection generally appeared to be lower than VE and rVE estimates that included successfully sequenced samples only. The VE and rVE against hospitalization were less impacted in the sensitivity analyses because imputation of SGTF data did not substantively change the numbers.

In the sensitivity analysis excluding immunocompromised individuals, the VE or rVE estimates against infection generally did not vary substantially from the main analyses, except that the point estimates of VE against BA.2 and BA.5 infection at >90 days after the fourth dose were higher; however, the CI still included 0. This also translated to higher rVE of four doses compared with three doses against the two subvariants, compared with the main analyses. In addition, the point estimate for three-dose VE against hospitalization for BA.4/BA.5 was higher in the sensitivity analysis 88.5% (95% CI 61.0%, 96.6%) compared with 72.4% (95% CI 23.9%, 90.0%) in the main analysis (Supplementary Tables 10a–13b).

Among those with a known history of SARS-CoV-2 infection, we present the frequency distribution of SARS-CoV-2 variants by mRNA-1273 vaccination status in Supplementary Table 14, and the three-dose versus unvaccinated VE and three-dose versus two-dose rVE against infection with omicron subvariants by time since third dose (Supplementary Tables 15a,b, respectively). Although the confidence intervals were wide, the point estimates for three-dose VE against BA.2, BA.2.12.1, BA.4, and BA.5 infection appeared to be higher in the subgroup with prior SARS-CoV-2 infection than in the overall study population (Supplementary Table 2a).

## Discussion

The study evaluated the effectiveness of three and four doses of mRNA-1273 against infection with and hospitalization for omicron subvariants in a large, racially, ethnically, and socioeconomically diverse population. The rapid emergence of several subvariants of omicron, particularly BA.4 and BA.5, which have markedly increased transmissibility and ability to evade natural and vaccine-elicited immunity, raise concerns about the ability of original monovalent COVID-19 vaccines to protect against SARS-COV-2 infections^2,16^. Using successfully sequenced results, we were able to focus our analyses on cases that tended to have a higher viral load and were more likely symptomatic. In addition, COVID-19 hospitalized cases met a prespecified, previously validated case definition or charts were reviewed to confirm hospitalization for severe COVID-19, rather than hospitalization that was coincident with COVID-19. The results provide relevant evidence of mRNA-1273 effectiveness in alleviating COVID-related disease burden in a real-world setting.

Our study found that three-dose VE of mRNA-1273 against infection with BA.1 was high and waned slowly, whereas VE against infection with more recent omicron subvariants, including BA.2, BA.2.12.1, BA.4, and BA.5, waned more rapidly. Our results are similar to those of a large, multicenter study in which initial three-dose mRNA VE against BA.1 was found to be greater with slower waning than that against BA.2 (84% [95% CI 83%, 85%] versus 56% [95% CI 51%, 61%] for ED and urgent care encounters at <120 days after dose 3 compared to our results of 76.3% [95% CI 73.9%, 78.6%] versus 41.2% [95% CI 28.3%, 51.8%] for infection at 31–90 days)^24^. Similarly, Chemaitelly et al. reported lower initial three-dose mRNA VE against BA.1 and BA.2 than we found, but assessed initial VE at <1 month and therefore included participants with infection at 1–13 days after vaccination when VE is lower than it is at 14–30 days; this may have accounted for some of the difference seen^9^. Likewise, Tartof and colleagues’ estimates of three-dose BNT162b2 VE against infection with BA.4 or BA.5 was 55–71% depending on the setting (outpatient, urgent care, or ED), which is slightly lower than our three-dose mRNA-1273 VE estimate of 72.6% to 90.6% at 14–30 days; however, their analysis of VE at <90 days included 31–90 days, during which our more granular analysis found waning against infection with these variants that was more substantial than that against infection with BA.1^29^.

Similarly, four-dose VE against infection with BA.2, BA.2.12.1, BA.4, and BA.5 was moderate, and was only approximately 35% against BA.5. The four-dose VE against these subvariants was short-lived, disappearing beyond 90 days after the fourth dose. Although most studies evaluating four-dose VE assessed relative four-dose versus three-dose VE against hospitalization and severe outcomes among older adults for whom the 4th dose was initially recommended, Link-Gelles et al. found four-dose mRNA VE against infection in the ED and urgent care setting was slightly greater (66%, 95%CI 60%, 71%) at ≥7 days than three-dose mRNA VE (58%, 95% CI 51%, 64%) at 7–119 days among adults ≥50 years^30^. However, the median time from vaccination to time of infection was substantially shorter among four-dose versus three-dose recipients (28 versus 96 days, respectively), likely contributing to the small difference observed. Taken together, these findings appear to be consistent with those of a recent study that found that the primary benefit of booster vaccines is augmentation of neutralizing antibodies without a strong effect on cellular immunity beyond that already induced by the primary vaccination series^31^. In a recent study, Qu et al. indicated that although the decay rate of booster neutralizing antibody was similar among variants, the omicron subvariants, especially BA.4 and BA.5, had substantial neutralization resistance. Their data suggest that both SARS-CoV-2 variant evolution and waning neutralizing antibody titers may reduce booster-induced immune protection^32^.

However, simultaneous with the emergence of increasingly immune-evasive omicron subvariants, infection-induced immunity in the population substantially increased during and after the BA.1 period. In the United States, overall seroprevalence estimates of infection-induced antibodies increased from 33.5% in December 2021 to 57.7% at the end of February 2022^33^. Although the increase in seroprevalence in adults was lower than that in children during this period, and we adjusted for calendar time and history of SARS-CoV-2 infection, we may not have been able to sufficiently control for natural immunity. Cumulative infections prior to BA.2, BA.4, and BA.5 periods were greater than they were prior to the BA.1 period, resulting in a larger proportion of unvaccinated participants acquiring infection-induced immunity. Such differential natural immunity and reduced susceptibility to infection likely biases our VE estimates downward. In some situations, prior infections among vaccinated participants could have provided greater hybrid immunity than prior infection in unvaccinated participants^34,35^. Therefore, the net impact of prior infection on VE estimates can be difficult to disentangle without complete information on history of infection. Our results show that protection from a booster dose of monovalent Wuhan strain-formulated mRNA-1273 against infection with the BA.4 and BA.5 subvariants could wane quickly, suggesting that updated bivalent vaccines may better protect against infection with emerging variants. However, the four-dose protection of monovalent mRNA-1273 against hospitalization for COVID-19 disease remains high, at least in the short term.

Although three-dose VE of mRNA-1273 against hospitalization was much higher than VE against infection for all omicron subvariants assessed, the three-dose VE against hospitalization for BA.2 and especially against hospitalization for BA.4/BA.5 was lower than that against hospitalization for BA.1. The results from sensitivity analyses suggest the three-dose VE against hospitalization for BA.4/BA.5 could be particularly low for immunocompromised individuals. Compared with three doses of mRNA-1273, four doses confer additional protection against hospitalization for either BA.2 or BA.4/BA.5. The durability against hospitalization for BA.4/BA.5 is still unknown. Monitoring for waning protection against hospitalization for BA.4/BA.5 or subsequent new subvariants that may emerge will be critically important as more data becomes available^19^.

This study provides important data on the effectiveness of mRNA-1273 against infection with and hospitalization for omicron subvariants, including predominant subvariants, BA.4 and BA.5. This study has several strengths and limitations. First, the results of our test-negative case-control study may not be generalizable to people who are not tested for SARS-CoV-2, including those with milder symptoms who may not seek testing in healthcare settings. There are several risk factors for infections or severe outcomes that may be associated with both testing and vaccination that could introduce bias, for example, mask use, social distancing, and hygiene practices. We attempted to reduce potential bias by adjusting for sociodemographic characteristics, prior healthcare use, prior SARS-CoV-2 testing, and comorbidities in the models, but residual confounding may remain. For example, some negative VE estimates observed at >150 days after vaccination could be due to differential risk behaviors among vaccinated and unvaccinated individuals when protection from antibodies becomes minimal. Second, as predominant subvariants evolved during the study period, many other factors could also change over time, such as practice of non-pharmacologic interventions, availability of antiviral medications or monoclonal antibody treatments, preventive public health policy, and individual behaviors. These changes might impact the comparison of VE across subvariants.

Third, while rapid antigen test results were included in the history of SARS-CoV-2 infection covariate, some at-home positive rapid antigen test results that were not self-reported may have been missed. Because both cases and controls had a PCR test performed at KPSC, we expect that the rate of under-reporting of at-home rapid antigen test results would be nondifferential between cases and controls, but it may have differed by vaccination status.

Fourth, misclassification of test-positive cases and test-negative controls was another possible source of bias. However, we used a highly specific and sensitive RT-PCR test that minimized misclassification and allowed us to monitor variant proportions through WGS and SGTF analysis. Similarly, misclassification of vaccination status was possible but likely minimal, as KPSC electronic health records (EHRs) captured all vaccinations administered within KPSC and were updated daily with vaccine administration data from the California Immunization Registry, to which all facilities are required by law to report COVID-19 vaccinations within 24 hours. In addition, inclusion of patients hospitalized for reasons other than COVID-19 who are found to have coincident SARS-CoV-2 infection with minimal or no symptoms could also introduce bias^36–38^. In this study, hospitalizations for COVID-19 were identified using a prespecified algorithm, or charts were reviewed to confirm severe COVID-19 disease leading to hospitalization, decreasing the possibility of spuriously reduced estimates of VE against severe disease^39^.

Fifth, statistical power might have been insufficient for testing VE against some subvariants that had lower numbers of cases, resulting in wide confidence intervals for some VE estimates. This was addressed by the sensitivity analysis using SGTF results, in which the VE estimates became more precise. Finally, multiple comparisons were not adjusted for in the analyses, as the focus of the study was on estimating clinically meaningful VE over time across subvariants, rather than statistical significance.

In conclusion, our data indicate that the three-dose or four-dose effectiveness of mRNA-1273 against infection with omicron subvariants is moderate and short-lived, but protection against severe COVID-19 disease remains robust. With the updated bivalent BA.4/BA.5–containing booster (mRNA-1273.222) available in the United States, it is imperative to continue to evaluate its effectiveness, durability, and impact on SARS-CoV-2 evolution.

## Methods

### Study setting

KPSC is an integrated health system that provides healthcare services and insurance coverage to >4.7 million members with sociodemographic characteristics representative of the diverse Southern California population^40,41^. EHRs comprehensively capture details of patient care, including vaccinations, diagnoses, laboratory tests, procedures, and pharmacy records. Although most members seek care at KPSC facilities (i.e., 15 hospitals and 236 medical offices), care received outside of KPSC is incorporated into the EHR as part of claims reimbursement. In addition, vaccinations received outside of KPSC are imported daily from external sources, including the California Immunizations Registry (CAIR), Care Everywhere (system on the Epic EHR platform that allows different healthcare systems to exchange patients’ medical information), claims (for example, retail pharmacies), and self-report by members (with valid documentation). The study protocol was submitted to regulatory agencies prior to the conduct of the study and is available in the Supplementary Material. The study was approved by the KPSC Institutional Review Board, which waived requirements for written informed consent and written Health Insurance Portability and Accountability Act authorization, as the use of EHRs for this observational study involved minimal risk.

### Laboratory methods

SARS-CoV-2 molecular diagnostic testing is conducted routinely at KPSC for members with and without symptoms who request testing for any reason and prior to certain procedures or hospital admission. Nasopharyngeal specimens (for symptomatic or asymptomatic individuals) or saliva specimens (for asymptomatic individuals) are tested using the RT-PCR TaqPath COVID-19 High-Throughput Combo Kit (Thermo Fisher Scientific, CA, USA). SGTF is defined as a SARS-CoV-2 positive specimen with N and ORF1ab genes detected (cycle threshold values <37), but with undetected S gene. Random samples of SARS-CoV-2–positive specimens are sent on a weekly basis to a commercial laboratory for WGS, as detailed in our prior publications^22,41^.

### Study design

We used a test-negative case-control design to assess the effectiveness of 3 and four doses of mRNA-1273 against SARS-CoV-2 omicron subvariants. Cases were identified from individuals with positive SARS-CoV-2 RT-PCR tests from specimens collected between 1/1/2022 and 6/30/2022 that were sent for WGS and controls that were identified from those with only negative SARS-CoV-2 RT-PCR tests during the same period. Individuals were included if they were aged ≥18 years and had ≥12 months of KPSC membership before the specimen collection date (necessary for accurate ascertainment of exposure status and covariates) and were excluded if they had a history of SARS-CoV-2 infection in the 90 days prior to specimen collection date, received any dose of mRNA-1273 < 14 days before the specimen collection date, received any two doses of mRNA-1273 < 24 days apart or >four doses of mRNA-1273 before the specimen collection date, or received a COVID-19 vaccine other than mRNA-1273. The first eligible positive and negative SARS-CoV-2 RT-PCR tests were included.

We matched cases and controls by a ratio of 1 to 3 on age (18–44 years, 45–64 years, 65–74 years, and ≥75 years), sex, race/ethnicity (non-Hispanic White, non-Hispanic Black, Hispanic, non-Hispanic Asian, and Other/Unknown), and specimen collection date (±10 days).

### Outcomes

Cases consisted of persons infected with BA.1, BA.2 (excluding BA.2.12.1), BA.2.12.1, BA.4, or BA.5, the omicron subvariants monitored by the World Health Organization that were circulating during the study period^6^. Other variants (e.g., delta, BA.2.75, BA.3, and recombinant lineages) were not analyzed due to low prevalence during the study period. COVID-19 hospitalization was defined as hospitalization *for* severe COVID-19, rather than hospitalization *with* coincident SARS-CoV-2 infection^36^. COVID-19 hospitalization was initially identified as a SARS-CoV-2–positive test ≤7 days prior to or during hospitalization and further confirmed by (1) ≥1 documented oxygen saturation (SpO_2_) of <90% during hospital stay for all patients or during a labor/delivery stay >2 days for pregnant patients or (2) manual chart review, as needed, performed by a physician investigator (B.K.A.) and trained chart abstractors to verify the presence of severe COVID-19 symptoms.

### Exposures

The study focused on mRNA-1273, as it was conducted as part of a regulatory commitment from Moderna to multiple health authorities. The mRNA-1273 product used during the study period was the original monovalent vaccine. The exposures of interest were three doses (versus two doses and versus unvaccinated) or four doses (versus three doses and versus unvaccinated) of mRNA-1273. We included both 50-µg and 100-µg doses for third and fourth doses, as dosing information was not available for vaccines given outside of KPSC.

### Covariates

We identified potential confounders a priori based on the literature. Variables collected from EHRs before specimen collection included age, sex, self-reported race/ethnicity, body mass index, smoking, Charlson comorbidity score, frailty index, chronic diseases, immunocompromised status, autoimmune conditions, healthcare visits (outpatient, virtual, ED, and inpatient), preventive care (other vaccinations, screenings, and wellness visits), history of SARS-CoV-2 infection, and history of SARS-CoV-2 molecular tests. Additional variables at date of specimen collection included pregnancy status, socioeconomic status (Medicaid and neighborhood median household income), KPSC physician/employee status, medical center area, month of specimen collection, and specimen type (nasopharyngeal versus saliva).

### Statistical analyses

We described the distribution of SARS-CoV-2 variants by mRNA-127 three dose and calendar time. We compared the characteristics of cases and controls using the *χ*^2^ test or Fisher exact test for categorical variables and two-sample *t* test or Wilcoxon rank-sum test for continuous variables, calculating the ASD to assess the balance of covariates. We used logistic regression adjusting for potential confounders to assess odds ratio (OR) and 95% CI for three doses versus unvaccinated or four doses versus unvaccinated of mRNA-1273 against infection and hospitalization with omicron subvariants (BA.1 [not assessed for four doses], BA.2 [excluding BA.2.12.1], BA.2.12.1, BA.4, and BA.5 [BA.4/BA.5 were combined for hospitalization models]). We calculated VE (%) as (1–OR) × 100 when OR was ≤1, and ([1/OR]–1) × 100 when OR was >1. We also assessed three-dose and four-dose effectiveness against infection with omicron subvariants by time since receipt of third or fourth dose of mRNA-1273 (for three-dose VE: 14–30 days, 31–90 days, 91–150 days, and >150 days since the third dose; for four-dose VE: 14–30 days, 31–90 days, and >90 days since the fourth dose).

To evaluate the incremental effectiveness of (a) three doses versus two doses and (b) four doses versus three doses of mRNA-1273, we further evaluated the rVE using the same approach as noted previously. Cases or controls receiving two doses or three doses of mRNA-1273, respectively, were combined as the comparison groups. The rVE by time since receipt of the third dose or the fourth dose against infection with omicron subvariants was also assessed (for three-dose versus two-dose rVE: 14–30 days, 31–90 days, 91–150 days, and >150 days since the third dose; for four-dose versus three-dose rVE: 14–30 days, 31–90 days, and >90 days since the fourth dose). rVE is interpreted as the incremental effectiveness of receiving an additional dose of mRNA-1273 compared with those who only received two doses or three doses, respectively.

Covariates included for adjustment across models were matching variables (age, sex, race/ethnicity, month of specimen collection) and other covariates with ASD > 0.1 and *P* < 0.1 from the comprehensive list of prespecified potential confounders. Additionally, we adjusted three-dose versus two-dose models for time between the second dose and specimen collection date and adjusted four-dose versus three-dose models for time between the third dose and specimen collection date to help account for possible differences in the timing of the second/third dose, respectively. SAS 9.4 software (SAS Institute) was used for all analyses.

We also conducted two sets of sensitivity analyses. In the first set of sensitivity analyses, we included cases that failed sequencing. For these analyses, according to the distribution of SARS-CoV-2 variants by month among successfully sequenced cases at KPSC, SGTF status and calendar month were used as a proxy to impute variant among cases that failed sequencing: specimens that were SGTF+ and collected during January–April 2022 were considered BA.1, those that were SGTF+ and collected during May–June 2022 were considered BA.4/BA.5 (combined, as it is not possible to distinguish BA.4 and BA.5 based on SGTF status), those that were SGTF- and were collected during January 2022 were considered delta, and those that were SGTF- and were collected during February–June 2022 were considered BA.2. In the second set of sensitivity analyses, we excluded immunocompromised subjects to estimate the VE and rVE in the immunocompetent subjects. Because separate analyses with immunocompromised subjects only were not feasible given the small numbers across subvariants, both immunocompetent and immunosuppressed subjects were included in the main analyses.

In exploratory analyses of individuals with a known history of SARS-CoV-2 infection, we characterized the frequency of omicron subvariants by mRNA-1273 vaccination status. We estimated the three-dose versus unvaccinated VE and three-dose versus two-dose rVE against infection with omicron subvariants by time since third dose, among those with a known history of SARS-CoV-2 infection. Due to insufficient sample size, we were unable to conduct a similar analysis among the four-dose recipients.

### Reporting summary

Further information on research design is available in the Nature Portfolio Reporting Summary linked to this article.

## Supplementary information

Supplementary Information

Reporting Summary

## Data Availability

Individual-level data reported in this study involving human research participants are not publicly shared due to potentially identifying or sensitive patient information. Upon request to the corresponding author [H.F.T.], and subject to review and approval of an analysis proposal, KPSC may provide the deidentified aggregate-level data that support the findings of this study within 6 months. Anonymized data (deidentified data including participant data as applicable) that support the findings of this study may be made available from the investigative team in the following conditions: (1) agreement to collaborate with the study team on all publications, (2) provision of external funding for administrative and investigator time necessary for this collaboration, (3) demonstration that the external investigative team is qualified and has documented evidence of training for human subjects protections, and (4) agreement to abide by the terms outlined in data use agreements between institutions.

## Code availability

Standard epidemiological analyses were conducted using standard commands in SAS 9.4 (SAS Institute, Cary NC). The commands/code are available at https://github.com/juliatubert/P901-Variant-VE-Manuscript---Nature-Comms (10.5281/zenodo.7387988)^42^.
