# Supplementary material for: Effectiveness of mRNA-1273 vaccination against SARS-CoV-2 omicron subvariants BA.1, BA.2, BA.2.12.1, BA.4, and BA.5
Source: Nat Commun. 2023 Jan 12;14:189. doi: 10.1038/s41467-023-35815-7 (PMC9836332; doi:10.1038/s41467-023-35815-7)
Supplement: Supplementary file 1 — Supplementary Information [file 41467_2023_35815_MOESM1_ESM.pdf]

Effectiveness of mRNA-1273 vaccination against SARS-CoV-2 Omicron subvariants BA.1, BA.2, BA.2.12.1, BA.4, and BA.5

SUPPLEMENTARY INFORMATION

Authors: Hung Fu Tseng,[1,2] Bradley K. Ackerson,[1] Katia J. Bruxvoort,[1,3] Lina S. Sy,[1] Julia E. Tubert,[1] Gina S. Lee,[1] Jennifer H. Ku,[1] Ana Florea,[1] Yi Luo,[1] Sijia Qiu,[1] Soon Kyu Choi,[1] Harpreet S. Takhar,[1] Michael Aragones,[1] Yamuna D. Paila,[4] Scott Chavers,[4] Carla A. Talarico,[4,5] Lei Qian[1]

Affiliations:

[1]Kaiser Permanente Southern California, 100 S Los Robles Ave., Pasadena, CA 91101, USA

[2]Kaiser Permanente Bernard J. Tyson School of Medicine, 98 S. Los Robles Ave., Pasadena, CA 91101, USA

[3]University of Alabama at Birmingham, 1665 University Blvd, Birmingham, AL 35233, USA

[4]Moderna Inc., 200 Technology Square, Cambridge, MA 02139, USA

[5]Astrazeneca, 1 Medimmune Way, Gaithersburg, MD 20878, USA

Corresponding Author:

Hung Fu Tseng, PhD

Department of Research and Evaluation, Kaiser Permanente Southern California, 100 S Los Robles, Pasadena, CA 91101

Phone: (626) 564-3451

Email: Hung-Fu.X.Tseng@kp.org

Contents

Supplementary Table 1. Characteristics of SARS-CoV-2 specimens, by sequencing status and mRNA-1273 vaccination status 4
Supplementary Figure 1. Distribution of SARS-CoV-2 variants by mRNA-1273 vaccination status 5
Supplementary Figure 2. Distribution of SARS-CoV-2 variants by month of specimen collection........ 6
Supplementary Table 2a. Vaccine effectiveness of 3 doses of mRNA-1273 against infection with SARS-CoV-2 variants by time since vaccination 7
Supplementary Table 2b. Relative vaccine effectiveness of 3 vs. 2 doses of mRNA-1273 against infection with SARS-CoV-2 variants by time since vaccination 7
Supplementary Table 3a. Vaccine effectiveness of 4 doses of mRNA-1273 against infection with SARS-CoV-2 variants by time since vaccination 9
Supplementary Table 3b. Relative vaccine effectiveness of 4 vs. 3 doses of mRNA-1273 against infection with SARS-CoV-2 variants by time since vaccination 10
Supplementary Table 4a. Vaccine effectiveness of 3 doses of mRNA-1273 against COVID-19 hospitalization associated with SARS-CoV-2 variants........ 11
Supplementary Table 4b. Relative vaccine effectiveness of 3 vs. 2 doses of mRNA-1273 against COVID-19 hospitalization associated with SARS-CoV-2 variants 12
Supplementary Table 5a. Vaccine effectiveness of 4 doses of mRNA-1273 against COVID-19 hospitalization associated with SARS-CoV-2 variants........ 13
Supplementary Table 5b. Relative vaccine effectiveness of 4 vs. 3 doses of mRNA-1273 against COVID-19 hospitalization associated with SARS-CoV-2 variants 14
Supplementary Table 6a. Vaccine effectiveness of 3 doses of mRNA-1273 against infection with SARS-CoV-2 variants by time since vaccination, using SGTF data to impute unidentified subvariants 15
Supplementary Table 6b. Relative vaccine effectiveness of 3 vs. 2 doses of mRNA-1273 against infection with SARS-CoV-2 variants by time since vaccination, using SGTF data to impute unidentified subvariants........ 16
Supplementary Table 7a. Vaccine effectiveness of 4 doses of mRNA-1273 against infection with SARS-CoV-2 variants by time since vaccination, using SGTF data to impute unidentified subvariants 17
Supplementary Table 7b. Relative vaccine effectiveness of 4 vs. 3 doses of mRNA-1273 against infection with SARS-CoV-2 variants by time since vaccination, using SGTF data to impute unidentified subvariants........ 18
Supplementary Table 8a. Vaccine effectiveness of 3 doses of mRNA-1273 against COVID-19 hospitalization associated with SARS-CoV-2 variants, using SGTF data to impute unidentified subvariants........ 19
Supplementary Table 8b. Relative vaccine effectiveness of 3 vs. 2 doses of mRNA-1273 against COVID-19 hospitalization associated with SARS-CoV-2 variants, using SGTF data to impute unidentified subvariants........ 20
Supplementary Table 9a. Vaccine effectiveness of 4 doses of mRNA-1273 against COVID-19 hospitalization associated with SARS-CoV-2 variants, using SGTF data to impute unidentified subvariants........ 21

Supplementary Table 9b. Relative vaccine effectiveness of 4 vs. 3 doses of mRNA-1273 against COVID-19 hospitalization associated with SARS-CoV-2 variants, using SGTF data to impute unidentified subvariants 22

Supplementary Table 10a. Vaccine effectiveness of 3 doses of mRNA-1273 against infection with SARS-CoV-2 variants by time since vaccination, excluding immunocompromised patients 23

Supplementary Table 10b. Relative vaccine effectiveness of 3 vs. 2 doses of mRNA-1273 against infection with SARS-CoV-2 variants by time since vaccination, excluding immunocompromised patients 24

Supplementary Table 11a. Vaccine effectiveness of 4 doses of mRNA-1273 against infection with SARS-CoV-2 variants by time since vaccination, excluding immunocompromised patients 25

Supplementary Table 11b. Relative vaccine effectiveness of 4 vs. 3 doses of mRNA-1273 against infection with SARS-CoV-2 variants by time since vaccination, excluding immunocompromised patients 26

Supplementary Table 12a. Vaccine effectiveness of 3 doses of mRNA-1273 against COVID-19 hospitalization associated with SARS-CoV-2 variants, excluding immunocompromised patients 27

Supplementary Table 12b. Relative vaccine effectiveness of 3 vs. 2 doses of mRNA-1273 against COVID-19 hospitalization associated with SARS-CoV-2 variants, excluding immunocompromised patients 28

Supplementary Table 13a. Vaccine effectiveness of 4 doses of mRNA-1273 against COVID-19 hospitalization associated with SARS-CoV-2 variants, excluding immunocompromised patients 29

Supplementary Table 13b. Relative vaccine effectiveness of 4 vs. 3 doses of mRNA-1273 against COVID-19 hospitalization associated with SARS-CoV-2 variants, excluding immunocompromised patients 30

Supplementary Table 14. Frequency distribution of SARS-CoV-2 positive cases among individuals with known history of SARS-CoV-2 infection, by vaccination status and variant........ 31

Supplementary Table 15a. Vaccine effectiveness of 3 doses of mRNA-1273 against infection with SARS-CoV-2 variants by time since vaccination among those with known history of SARS-CoV-2 infection........ 32

Supplementary Table 15b. Relative vaccine effectiveness of 3 vs. 2 doses of mRNA-1273 against infection with SARS-CoV-2 variants by time since vaccination among those with known history of SARS-CoV-2 infection........ 34

Study Protocol 36

Supplementary Table 1. Characteristics of SARS-CoV-2 specimens, by sequencing status and mRNA-1273 vaccination status

	Sequencing Success						Sequencing Failure					
	Vaccinated 1 dose	Vaccinated 2 dose	Vaccinated 3 dose	Vaccinated 4 dose	Unvaccinated	Total	Vaccinated 1 dose	Vaccinated 2 dose	Vaccinated 3 dose	Vaccinated 4 dose	Unvaccinated	Total
Overall, n	158	3701	6887	531	5141	16418	120	3231	5837	435	4768	14391
Specimen type, n (%)												
Nasopharyngeal/oropharyngeal swab	147 (93.0%)	3286 (88.8%)	6022 (87.4%)	478 (90.0%)	4384 (85.3%)	14317 (87.2%)	102 (85.0%)	2615 (80.9%)	4622 (79.2%)	350 (80.5%)	3381 (70.9%)	11070 (76.9%)
Saliva	11 (7.0%)	415 (11.2%)	865 (12.6%)	53 (10.0%)	757 (14.7%)	2101 (12.8%)	18 (15.0%)	616 (19.1%)	1215 (20.8%)	85 (19.5%)	1387 (29.1%)	3321 (23.1%)
Composite Ct value, n (%)[a]												
≤27	145 (91.8%)	3403 (91.9%)	6488 (94.2%)	514 (96.8%)	4757 (92.5%)	15307 (93.2%)	12 (10.0%)	390 (12.1%)	838 (14.4%)	63 (14.5%)	580 (12.2%)	1883 (13.1%)
>27	12 (7.6%)	297 (8.0%)	394 (5.7%)	17 (3.2%)	379 (7.4%)	1099 (6.7%)	64 (53.3%)	1573 (48.7%)	3229 (55.3%)	282 (64.8%)	2296 (48.2%)	7444 (51.7%)
Missing	1 (0.6%)	1 (0.0%)	5 (0.1%)	0 (0.0%)	5 (0.1%)	12 (0.1%)	44 (36.7%)	1268 (39.2%)	1770 (30.3%)	90 (20.7%)	1892 (39.7%)	5064 (35.2%)
SGTF data available, n (%)	110 (69.6%)	2786 (75.3%)	5647 (82.0%)	419 (78.9%)	3495 (68.0%)	12457 (75.9%)	96 (80.0%)	2664 (82.5%)	5125 (87.8%)	385 (88.5%)	3736 (78.4%)	12006 (83.4%)
COVID-19 hospitalization, n (%)	7 (4.4%)	144 (3.9%)	119 (1.7%)	11 (2.1%)	392 (7.6%)	673 (4.1%)	2 (1.7%)	64 (2.0%)	89 (1.5%)	11 (2.5%)	190 (4.0%)	356 (2.5%)
COVID-19 hospital death, n (%)	3 (1.9%)	7 (0.2%)	13 (0.2%)	0 (0.0%)	58 (1.1%)	81 (0.5%)	0 (0.0%)	2 (0.1%)	8 (0.1%)	0 (0.0%)	18 (0.4%)	28 (0.2%)
Variant, n (%)												
BA.1	65 (41.1%)	1832 (49.5%)	1392 (20.2%)	3 (0.6%)	2482 (48.3%)	5774 (35.2%)	N/A	N/A	N/A	N/A	N/A	N/A
BA.2[b]	48 (30.4%)	843 (22.8%)	2451 (35.6%)	164 (30.9%)	1190 (23.1%)	4696 (28.6%)	N/A	N/A	N/A	N/A	N/A	N/A
BA.2.12.1	30 (19.0%)	627 (16.9%)	1880 (27.3%)	176 (33.1%)	798 (15.5%)	3511 (21.4%)	N/A	N/A	N/A	N/A	N/A	N/A
BA.3	0 (0.0%)	0 (0.0%)	2 (0.0%)	0 (0.0%)	2 (0.0%)	4 (0.0%)	N/A	N/A	N/A	N/A	N/A	N/A
BA.4	4 (2.5%)	106 (2.9%)	307 (4.5%)	38 (7.2%)	157 (3.1%)	612 (3.7%)	N/A	N/A	N/A	N/A	N/A	N/A
BA.5	11 (7.0%)	277 (7.5%)	836 (12.1%)	148 (27.9%)	433 (8.4%)	1705 (10.4%)	N/A	N/A	N/A	N/A	N/A	N/A
Delta	0 (0.0%)	9 (0.2%)	1 (0.0%)	0 (0.0%)	73 (1.4%)	83 (0.5%)	N/A	N/A	N/A	N/A	N/A	N/A
Recombinant	0 (0.0%)	7 (0.2%)	18 (0.3%)	2 (0.4%)	6 (0.1%)	33 (0.2%)	N/A	N/A	N/A	N/A	N/A	N/A

[a] Cycle threshold (Ct) values from RT-qPCR performed at Helix OpCo, LLC, using a single signal for the combined S and N genes.

[b] BA.2 excluding BA.2.12.1

N/A, not applicable; SGTF, S gene target failure.

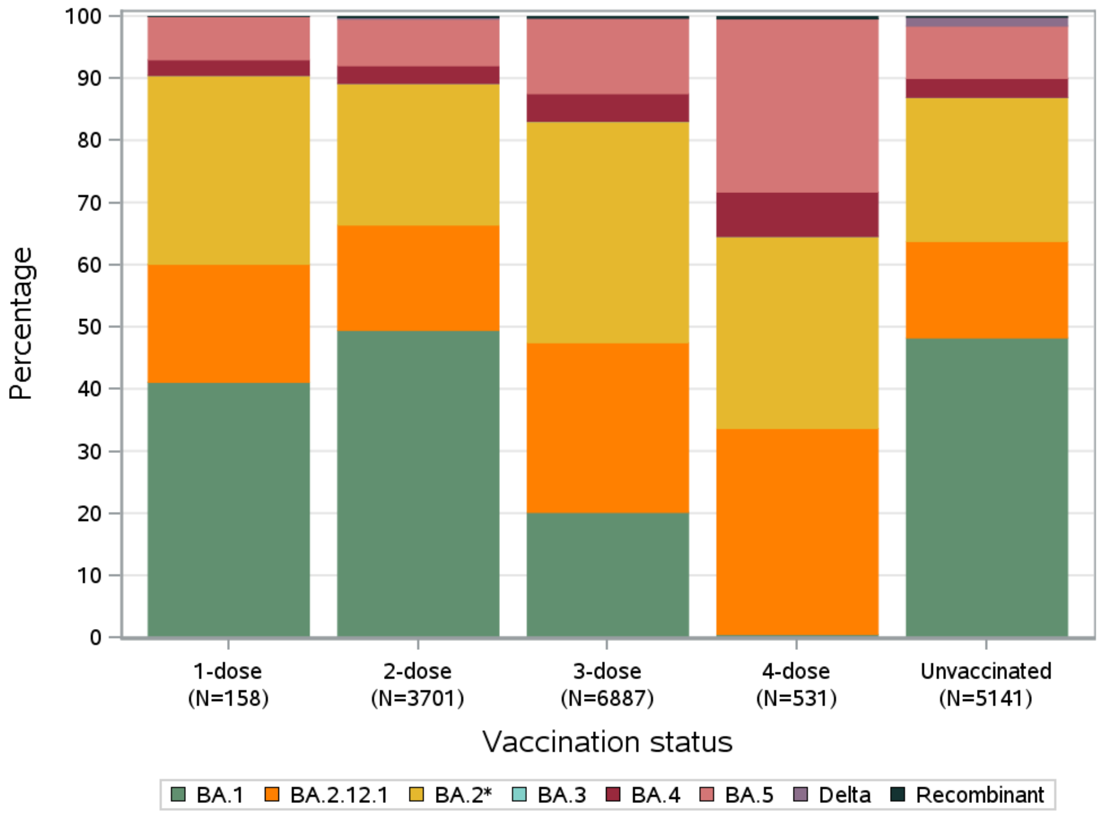

* BA.2 excluding BA.2.12.1

Supplementary Figure 1. Distribution of SARS-CoV-2 variants by mRNA-1273 vaccination status. Positive SARS-CoV-2 specimens collected at KPSC in the course of routine clinical practice were whole genome sequenced, and the distribution of identified SARS-CoV-2 variants are shown by mRNA-1273 vaccination status.

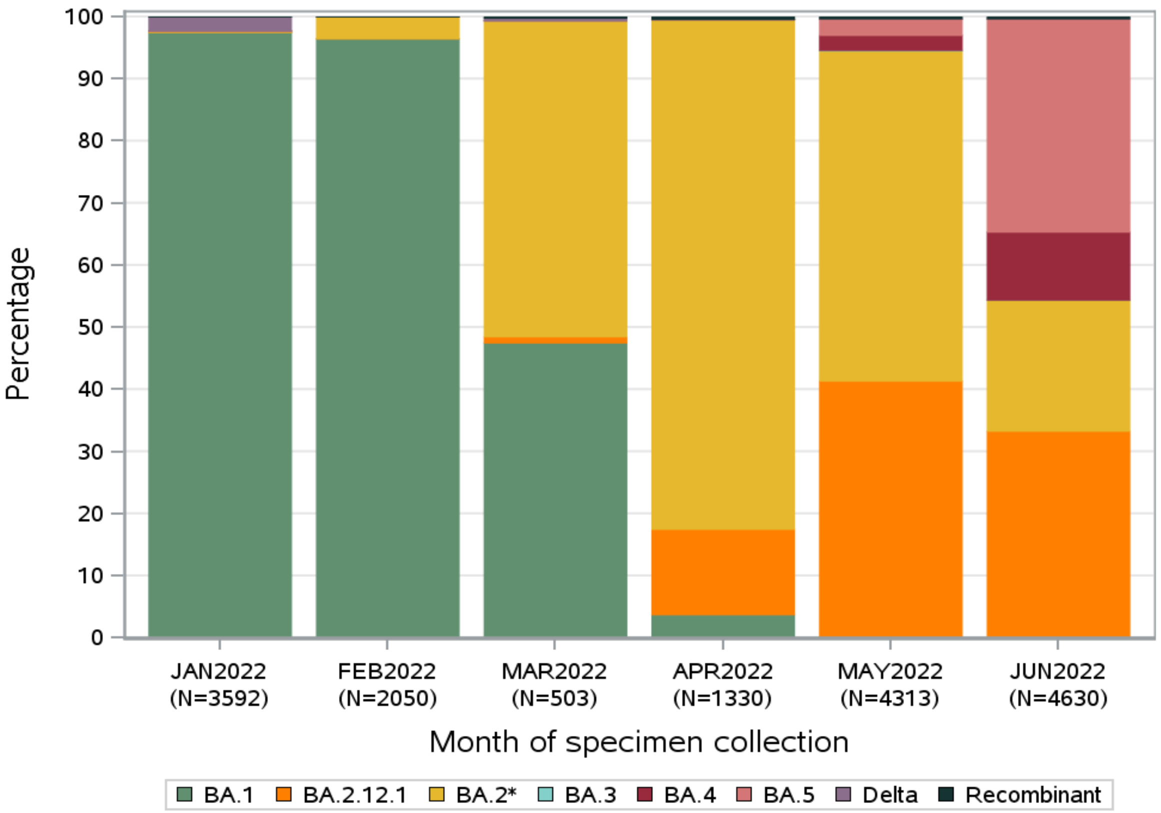

* BA.2 excluding BA.2.12.1

Supplementary Figure 2. Distribution of SARS-CoV-2 variants by month of specimen collection. Positive SARS-CoV-2 specimens collected at KPSC in the course of routine clinical practice were whole genome sequenced, and the distribution of identified SARS-CoV-2 variants are shown by the month of specimen collection.

Supplementary Table 2a. Vaccine effectiveness of 3 doses of mRNA-1273 against infection with SARS-CoV-2 variants by time since vaccination

Subvariant/Time since 3rd dose	Test Positive		Test Negative		Odds Ratio (95% CI)		VE (95% CI)[a]	
	3-dose Vaccinated (%)	Unvaccinated (%)	3-dose Vaccinated (%)	Unvaccinated (%)	Unadjusted	Adjusted[b]	Unadjusted	Adjusted[b]
BA.1	1392 (35.9%)	2482 (64.1%)	8371 (66.0%)	4305 (34.0%)	0.288 (0.268, 0.311)	0.234 (0.214, 0.256)	71.2% (68.9%, 73.2%)	76.6% (74.4%, 78.6%)
14-30 days	127 (4.9%)	2482 (95.1%)	1464 (25.4%)	4305 (74.6%)	0.150 (0.125, 0.182)	0.142 (0.117, 0.173)	85.0% (81.8%, 87.5%)	85.8% (82.7%, 88.3%)
31-90 days	874 (26.0%)	2482 (74.0%)	5427 (55.8%)	4305 (44.2%)	0.279 (0.256, 0.305)	0.237 (0.214, 0.261)	72.1% (69.5%, 74.4%)	76.3% (73.9%, 78.6%)
91-150 days	343 (12.1%)	2482 (87.9%)	1352 (23.9%)	4305 (76.1%)	0.440 (0.387, 0.500)	0.327 (0.281, 0.380)	56.0% (50.0%, 61.3%)	67.3% (62.0%, 71.9%)
>150 days	48 (1.9%)	2482 (98.1%)	128 (2.9%)	4305 (97.1%)	0.650 (0.465, 0.910)	0.451 (0.316, 0.644)	35.0% (9.0%, 53.5%)	54.9% (35.6%, 68.4%)
BA.2[c,d]	2451 (67.3%)	1190 (32.7%)	6934 (64.4%)	3827 (35.6%)	1.137 (1.050, 1.231)	1.022 (0.936, 1.117)	-12.0% (-18.8%, -4.7%)	-2.2% (-10.5%, 6.4%)
14-30 days	12 (1.0%)	1190 (99.0%)	93 (2.4%)	3827 (97.6%)	0.415 (0.227, 0.760)	0.390 (0.210, 0.724)	58.5% (24.0%, 77.3%)	61.0% (27.6%, 79.0%)
31-90 days	142 (10.7%)	1190 (89.3%)	803 (17.3%)	3827 (82.7%)	0.569 (0.470, 0.688)	0.588 (0.482, 0.717)	43.1% (31.2%, 53.0%)	41.2% (28.3%, 51.8%)
91-150 days	895 (42.9%)	1190 (57.1%)	3125 (45.0%)	3827 (55.0%)	0.921 (0.834, 1.017)	0.892 (0.802, 0.992)	7.9% (-1.6%, 16.6%)	10.8% (0.8%, 19.8%)
>150 days	1402 (54.1%)	1190 (45.9%)	2913 (43.2%)	3827 (56.8%)	1.548 (1.413, 1.695)	1.332 (1.201, 1.477)	-35.4% (-41.0%, -29.2%)	-24.9% (-32.3%, -16.7%)
BA.2.12.1[d]	1880 (70.2%)	798 (29.8%)	4963 (63.6%)	2841 (36.4%)	1.349 (1.227, 1.483)	1.134 (1.022, 1.258)	-25.8% (-32.6%, -18.5%)	-11.8% (-20.5%, -2.1%)
14-30 days	3 (0.4%)	798 (99.6%)	59 (2.0%)	2841 (98.0%)	0.181 (0.057, 0.579)	0.173 (0.053, 0.558)	81.9% (42.1%, 94.3%)	82.7% (44.2%, 94.7%)
31-90 days	52 (6.1%)	798 (93.9%)	310 (9.8%)	2841 (90.2%)	0.597 (0.441, 0.809)	0.628 (0.460, 0.859)	40.3% (19.1%, 55.9%)	37.2% (14.1%, 54.0%)
91-150 days	499 (38.5%)	798 (61.5%)	1764 (38.3%)	2841 (61.7%)	1.007 (0.887, 1.143)	0.902 (0.788, 1.032)	-0.7% (-12.5%, 11.3%)	9.8% (-3.1%, 21.2%)
>150 days	1326 (62.4%)	798 (37.6%)	2830 (49.9%)	2841 (50.1%)	1.668 (1.506, 1.847)	1.366 (1.219, 1.530)	-40.1% (-45.9%, -33.6%)	-26.8% (-34.6%, -18.0%)
BA.4	307 (66.2%)	157 (33.8%)	792 (61.2%)	502 (38.8%)	1.239 (0.992, 1.548)	1.077 (0.836, 1.388)	-19.3% (-35.4%, 0.8%)	-7.2% (-27.9%, 16.4%)
14-30 days	1 (0.6%)	157 (99.4%)	11 (2.1%)	502 (97.9%)	0.291 (0.037, 2.270)	0.274 (0.034, 2.207)	70.9% (-55.9%, 96.3%)	72.6% (-54.7%, 96.6%)
31-90 days	10 (6.0%)	157 (94.0%)	29 (5.5%)	502 (94.5%)	1.103 (0.526, 2.313)	0.993 (0.458, 2.154)	-9.3% (-56.8%, 47.4%)	0.7% (-53.6%, 54.2%)
91-150 days	46 (22.7%)	157 (77.3%)	185 (26.9%)	502 (73.1%)	0.795 (0.550, 1.150)	0.768 (0.517, 1.140)	20.5% (-13.1%, 45.0%)	23.2% (-12.3%, 48.3%)
>150 days	250 (61.4%)	157 (38.6%)	567 (53.0%)	502 (47.0%)	1.410 (1.117, 1.780)	1.196 (0.918, 1.558)	-29.1% (-43.8%, -10.5%)	-16.4% (-35.8%, 8.2%)
BA.5[d]	836 (65.9%)	433 (34.1%)	2265 (62.5%)	1358 (37.5%)	1.158 (1.012, 1.324)	1.075 (0.928, 1.246)	-13.6% (-24.5%, -1.2%)	-7.0% (-19.8%, 7.2%)
14-30 days	1 (0.2%)	433 (99.8%)	31 (2.2%)	1358 (97.8%)	0.101 (0.014, 0.743)	0.094 (0.013, 0.694)	89.9% (25.7%, 98.6%)	90.6% (30.6%, 98.7%)
31-90 days	16 (3.6%)	433 (96.4%)	116 (7.9%)	1358 (92.1%)	0.433 (0.254, 0.738)	0.430 (0.250, 0.738)	56.7% (26.2%, 74.6%)	57.0% (26.2%, 75.0%)
91-150 days	104 (19.4%)	433 (80.6%)	422 (23.7%)	1358 (76.3%)	0.773 (0.608, 0.983)	0.793 (0.618, 1.016)	22.7% (1.7%, 39.2%)	20.7% (-1.6%, 38.2%)
>150 days	715 (62.3%)	433 (37.7%)	1696 (55.5%)	1358 (44.5%)	1.322 (1.151, 1.519)	1.218 (1.044, 1.420)	-24.4% (-34.2%, -13.1%)	-17.9% (-29.6%, -4.2%)

[a] When the OR or its 95% CI was >1, the VE or its 95% CI was transformed as ([1/OR] – 1) x 100.

[b] Adjusted for age, sex, race/ethnicity, month of specimen collection, history of SARS-CoV-2 infection, history of SARS-CoV-2 molecular test, number of outpatient and virtual visits, and medical center area.
[c] BA.2 excluding BA.2.12.1.
[d] Medical center area removed from adjustment set due to lack of model convergence.
CI, confidence interval; OR, odds ratio; VE, vaccine effectiveness.

Supplementary Table 2b. Relative vaccine effectiveness of 3 versus 2 doses of mRNA-1273 against infection with SARS-CoV-2 variants by time since vaccination

Subvariant/Time since 3rd dose	Test Positive		Test Negative		Odds Ratio (95% CI)		rVE (95% CI)[a]	
	3-dose Vaccinated (%)	2-dose Vaccinated (%)	3-dose Vaccinated (%)	2-dose Vaccinated (%)	Unadjusted	Adjusted[b]	Unadjusted	Adjusted[b]
BA.1[c]	1392 (43.2%)	1832 (56.8%)	8371 (65.3%)	4447 (34.7%)	0.404 (0.373, 0.437)	0.297 (0.270, 0.326)	59.6% (56.3%, 62.7%)	70.3% (67.4%, 73.0%)
14-30 days	127 (6.5%)	1832 (93.5%)	1464 (24.8%)	4447 (75.2%)	0.211 (0.174, 0.255)	0.190 (0.156, 0.230)	78.9% (74.5%, 82.6%)	81.0% (77.0%, 84.4%)
31-90 days	874 (32.3%)	1832 (67.7%)	5427 (55.0%)	4447 (45.0%)	0.391 (0.357, 0.428)	0.312 (0.281, 0.345)	60.9% (57.2%, 64.3%)	68.8% (65.5%, 71.9%)
91-150 days	343 (15.8%)	1832 (84.2%)	1352 (23.3%)	4447 (76.7%)	0.616 (0.541, 0.702)	0.380 (0.325, 0.445)	38.4% (29.8%, 45.9%)	62.0% (55.5%, 67.5%)
>150 days	48 (2.6%)	1832 (97.4%)	128 (2.8%)	4447 (97.2%)	0.910 (0.650, 1.274)	0.500 (0.349, 0.716)	9.0% (-21.5%, 35.0%)	50.0% (28.4%, 65.1%)
BA.2[c,d]	2451 (74.4%)	843 (25.6%)	6934 (73.8%)	2459 (26.2%)	1.031 (0.941, 1.129)	0.864 (0.779, 0.958)	-3.0% (-11.4%, 5.9%)	13.6% (4.2%, 22.1%)
14-30 days	12 (1.4%)	843 (98.6%)	93 (3.6%)	2459 (96.4%)	0.376 (0.205, 0.690)	0.364 (0.196, 0.677)	62.4% (31.0%, 79.5%)	63.6% (32.3%, 80.4%)
31-90 days	142 (14.4%)	843 (85.6%)	803 (24.6%)	2459 (75.4%)	0.516 (0.425, 0.627)	0.515 (0.419, 0.633)	48.4% (37.3%, 57.5%)	48.5% (36.7%, 58.1%)
91-150 days	895 (51.5%)	843 (48.5%)	3125 (56.0%)	2459 (44.0%)	0.835 (0.750, 0.931)	0.775 (0.688, 0.872)	16.5% (6.9%, 25.0%)	22.5% (12.8%, 31.2%)
>150 days	1402 (62.4%)	843 (37.6%)	2913 (54.2%)	2459 (45.8%)	1.404 (1.269, 1.553)	1.158 (1.026, 1.306)	-28.8% (-35.6%, -21.2%)	-13.6% (-23.4%, -2.5%)
BA.2.12.1[c]	1880 (75.0%)	627 (25.0%)	4963 (73.4%)	1796 (26.6%)	1.085 (0.977, 1.206)	0.905 (0.803, 1.020)	-7.8% (-17.0%, 2.3%)	9.5% (-2.0%, 19.7%)
14-30 days	3 (0.5%)	627 (99.5%)	59 (3.2%)	1796 (96.8%)	0.146 (0.046, 0.466)	0.148 (0.046, 0.478)	85.4% (53.4%, 95.4%)	85.2% (52.2%, 95.4%)
31-90 days	52 (7.7%)	627 (92.3%)	310 (14.7%)	1796 (85.3%)	0.480 (0.353, 0.653)	0.550 (0.400, 0.755)	52.0% (34.7%, 64.7%)	45.0% (24.5%, 60.0%)
91-150 days	499 (44.3%)	627 (55.7%)	1764 (49.6%)	1796 (50.4%)	0.810 (0.708, 0.927)	0.752 (0.650, 0.871)	19.0% (7.3%, 29.2%)	24.8% (12.9%, 35.0%)
>150 days	1326 (67.9%)	627 (32.1%)	2830 (61.2%)	1796 (38.8%)	1.342 (1.200, 1.501)	1.100 (0.965, 1.255)	-25.5% (-33.4%, -16.7%)	-9.1% (-20.3%, 3.5%)
BA.4	307 (74.3%)	106 (25.7%)	792 (70.1%)	338 (29.9%)	1.236 (0.958, 1.595)	1.011 (0.755, 1.353)	-19.1% (-37.3%, 4.2%)	-1.1% (-26.1%, 24.5%)
14-30 days	1 (0.9%)	106 (99.1%)	11 (3.2%)	338 (96.8%)	0.290 (0.037, 2.272)	0.282 (0.035, 2.260)	71.0% (-56.0%, 96.3%)	71.8% (-55.7%, 96.5%)
31-90 days	10 (8.6%)	106 (91.4%)	29 (7.9%)	338 (92.1%)	1.100 (0.519, 2.330)	1.089 (0.500, 2.371)	-9.1% (-57.1%, 48.1%)	-8.2% (-57.8%, 50.0%)
91-150 days	46 (30.3%)	106 (69.7%)	185 (35.4%)	338 (64.6%)	0.793 (0.537, 1.171)	0.709 (0.468, 1.074)	20.7% (-14.6%, 46.3%)	29.1% (-6.9%, 53.2%)
>150 days	250 (70.2%)	106 (29.8%)	567 (62.7%)	338 (37.3%)	1.406 (1.080, 1.831)	1.151 (0.844, 1.570)	-28.9% (-45.4%, -7.4%)	-13.1% (-36.3%, 15.6%)
BA.5	836 (75.1%)	277 (24.9%)	2265 (72.8%)	846 (27.2%)	1.127 (0.963, 1.319)	1.041 (0.870, 1.246)	-11.3% (-24.2%, 3.7%)	-3.9% (-19.7%, 13.0%)
14-30 days	1 (0.4%)	277 (99.6%)	31 (3.5%)	846 (96.5%)	0.099 (0.013, 0.725)	0.090 (0.012, 0.664)	90.1% (27.5%, 98.7%)	91.0% (33.6%, 98.8%)
31-90 days	16 (5.5%)	277 (94.5%)	116 (12.1%)	846 (87.9%)	0.421 (0.245, 0.723)	0.467 (0.269, 0.811)	57.9% (27.7%, 75.5%)	53.3% (18.9%, 73.1%)
91-150 days	104 (27.3%)	277 (72.7%)	422 (33.3%)	846 (66.7%)	0.753 (0.584, 0.970)	0.820 (0.627, 1.071)	24.7% (3.0%, 41.6%)	18.0% (-6.7%, 37.3%)
>150 days	715 (72.1%)	277 (27.9%)	1696 (66.7%)	846 (33.3%)	1.288 (1.096, 1.513)	1.206 (0.998, 1.457)	-22.3% (-33.9%, -8.7%)	-17.1% (-31.4%, 0.2%)

[a] When the OR or its 95% CI was >1, the rVE or its 95% CI was transformed as ([1/OR] – 1) x 100.

[b] Adjusted for age, sex, race/ethnicity, month of specimen collection, history of SARS-CoV-2 infection, history of SARS-CoV-2 molecular test, number of outpatient and virtual visits, medical center area, and time between second dose and specimen collection date.

[c] Medical center area removed from adjustment set due to lack of model convergence.

[d] BA.2 excluding BA.2.12.1.

CI, confidence interval; OR, odds ratio; rVE, relative vaccine effectiveness.

Supplementary Table 3a. Vaccine effectiveness of 4 doses of mRNA-1273 against infection with SARS-CoV-2 variants by time since vaccination

Subvariant/Time since 4th dose	Test Positive		Test Negative		Odds Ratio (95% CI)		VE (95% CI)[a]	
	4-dose Vaccinated (%)	Unvaccinated (%)	4-dose Vaccinated (%)	Unvaccinated (%)	Unadjusted	Adjusted[b]	Unadjusted	Adjusted[b]
BA.2[c]	164 (12.1%)	1190 (87.9%)	740 (16.2%)	3827 (83.8%)	0.713 (0.595, 0.854)	0.443 (0.351, 0.558)	28.7% (14.6%, 40.5%)	55.7% (44.2%, 64.9%)
14-30 days	57 (4.6%)	1190 (95.4%)	327 (7.9%)	3827 (92.1%)	0.561 (0.420, 0.748)	0.357 (0.258, 0.493)	43.9% (25.2%, 58.0%)	64.3% (50.7%, 74.2%)
31-90 days	98 (7.6%)	1190 (92.4%)	388 (9.2%)	3827 (90.8%)	0.812 (0.645, 1.023)	0.489 (0.370, 0.645)	18.8% (-2.3%, 35.5%)	51.1% (35.5%, 63.0%)
>90 days	9 (0.8%)	1190 (99.2%)	25 (0.6%)	3827 (99.4%)	1.158 (0.539, 2.487)	0.827 (0.374, 1.828)	-13.6% (-59.8%, 46.1%)	17.3% (-45.3%, 62.6%)
BA.2.12.1[d]	176 (18.1%)	798 (81.9%)	834 (22.7%)	2841 (77.3%)	0.751 (0.627, 0.900)	0.547 (0.433, 0.690)	24.9% (10.0%, 37.3%)	45.3% (31.0%, 56.7%)
14-30 days	41 (4.9%)	798 (95.1%)	308 (9.8%)	2841 (90.2%)	0.474 (0.339, 0.663)	0.356 (0.246, 0.514)	52.6% (33.7%, 66.1%)	64.4% (48.6%, 75.4%)
31-90 days	127 (13.7%)	798 (86.3%)	499 (14.9%)	2841 (85.1%)	0.906 (0.734, 1.118)	0.645 (0.496, 0.839)	9.4% (-10.5%, 26.6%)	35.5% (16.1%, 50.4%)
>90 days	8 (1.0%)	798 (99.0%)	27 (0.9%)	2841 (99.1%)	1.055 (0.477, 2.331)	0.860 (0.381, 1.938)	-5.2% (-57.1%, 52.3%)	14.0% (-48.4%, 61.9%)
BA.4[d]	38 (19.5%)	157 (80.5%)	192 (27.7%)	502 (72.3%)	0.633 (0.428, 0.936)	0.452 (0.273, 0.749)	36.7% (6.4%, 57.2%)	54.8% (25.1%, 72.7%)
14-30 days	5 (3.1%)	157 (96.9%)	53 (9.5%)	502 (90.5%)	0.302 (0.119, 0.768)	0.243 (0.090, 0.653)	69.8% (23.2%, 88.1%)	75.7% (34.7%, 91.0%)
31-90 days	28 (15.1%)	157 (84.9%)	129 (20.4%)	502 (79.6%)	0.694 (0.444, 1.084)	0.491 (0.279, 0.866)	30.6% (-7.8%, 55.6%)	50.9% (13.4%, 72.1%)
>90 days	5 (3.1%)	157 (96.9%)	10 (2.0%)	502 (98.0%)	1.599 (0.538, 4.747)	0.937 (0.296, 2.966)	-37.5% (-78.9%, 46.2%)	6.3% (-66.3%, 70.4%)
BA.5[d]	148 (25.5%)	433 (74.5%)	602 (30.7%)	1358 (69.3%)	0.771 (0.625, 0.951)	0.657 (0.492, 0.878)	22.9% (4.9%, 37.5%)	34.3% (12.2%, 50.8%)
14-30 days	29 (6.3%)	433 (93.7%)	115 (7.8%)	1358 (92.2%)	0.791 (0.519, 1.205)	0.692 (0.435, 1.101)	20.9% (-17.0%, 48.1%)	30.8% (-9.2%, 56.5%)
31-90 days	112 (20.6%)	433 (79.4%)	466 (25.5%)	1358 (74.5%)	0.754 (0.597, 0.952)	0.633 (0.464, 0.864)	24.6% (4.8%, 40.3%)	36.7% (13.6%, 53.6%)
>90 days	7 (1.6%)	433 (98.4%)	21 (1.5%)	1358 (98.5%)	1.045 (0.441, 2.476)	0.950 (0.389, 2.321)	-4.3% (-59.6%, 55.9%)	5.0% (-56.9%, 61.1%)

[a] When the OR or its 95% CI was >1, the VE or its 95% CI was transformed as ([1/OR] – 1) x 100.

[b] Adjusted for age, sex, race/ethnicity, month of specimen collection, history of SARS-CoV-2 infection, history of SARS-CoV-2 molecular test, number of outpatient and virtual visits, and medical center area.

[c] BA.2 excluding BA.2.12.1.

[d] Medical center area removed from adjustment set due to lack of model convergence.

CI, confidence interval; OR, odds ratio; VE, vaccine effectiveness.

Supplementary Table 3b. Relative vaccine effectiveness of 4 versus 3 doses of mRNA-1273 against infection with SARS-CoV-2 variants by time since vaccination

Subvariant/Time since 4th dose	Test Positive		Test Negative		Odds Ratio (95% CI)		rVE (95% CI)[a]	
	4-dose Vaccinated (%)	3-dose Vaccinated (%)	4-dose Vaccinated (%)	3-dose Vaccinated (%)	Unadjusted	Adjusted[b]	Unadjusted	Adjusted[b]
BA.2[c,d]	164 (6.3%)	2451 (93.7%)	740 (9.6%)	6934 (90.4%)	0.627 (0.526, 0.747)	0.404 (0.332, 0.491)	37.3% (25.3%, 47.4%)	59.6% (50.9%, 66.8%)
14-30 days	57 (2.3%)	2451 (97.7%)	327 (4.5%)	6934 (95.5%)	0.493 (0.371, 0.656)	0.357 (0.265, 0.480)	50.7% (34.4%, 62.9%)	64.3% (52.0%, 73.5%)
31-90 days	98 (3.8%)	2451 (96.2%)	388 (5.3%)	6934 (94.7%)	0.715 (0.570, 0.896)	0.446 (0.349, 0.571)	28.5% (10.4%, 43.0%)	55.4% (42.9%, 65.1%)
>90 days	9 (0.4%)	2451 (99.6%)	25 (0.4%)	6934 (99.6%)	1.018 (0.475, 2.185)	0.339 (0.147, 0.779)	-1.8% (-54.2%, 52.5%)	66.1% (22.1%, 85.3%)
BA.2.12.1[d]	176 (8.6%)	1880 (91.4%)	834 (14.4%)	4963 (85.6%)	0.557 (0.470, 0.661)	0.402 (0.331, 0.488)	44.3% (33.9%, 53.0%)	59.8% (51.2%, 66.9%)
14-30 days	41 (2.1%)	1880 (97.9%)	308 (5.8%)	4963 (94.2%)	0.351 (0.253, 0.489)	0.279 (0.198, 0.392)	64.9% (51.1%, 74.7%)	72.1% (60.8%, 80.2%)
31-90 days	127 (6.3%)	1880 (93.7%)	499 (9.1%)	4963 (90.9%)	0.672 (0.549, 0.822)	0.483 (0.385, 0.605)	32.8% (17.8%, 45.1%)	51.7% (39.5%, 61.5%)
>90 days	8 (0.4%)	1880 (99.6%)	27 (0.5%)	4963 (99.5%)	0.782 (0.355, 1.725)	0.320 (0.137, 0.747)	21.8% (-42.0%, 64.5%)	68.0% (25.3%, 86.3%)
BA.4	38 (11.0%)	307 (89.0%)	192 (19.5%)	792 (80.5%)	0.511 (0.352, 0.741)	0.370 (0.238, 0.575)	48.9% (25.9%, 64.8%)	63.0% (42.5%, 76.2%)
14-30 days	5 (1.6%)	307 (98.4%)	53 (6.3%)	792 (93.7%)	0.243 (0.096, 0.615)	0.192 (0.073, 0.504)	75.7% (38.5%, 90.4%)	80.8% (49.6%, 92.7%)
31-90 days	28 (8.4%)	307 (91.6%)	129 (14.0%)	792 (86.0%)	0.560 (0.364, 0.860)	0.431 (0.261, 0.712)	44.0% (14.0%, 63.6%)	56.9% (28.8%, 73.9%)
>90 days	5 (1.6%)	307 (98.4%)	10 (1.2%)	792 (98.8%)	1.290 (0.437, 3.804)	0.576 (0.171, 1.943)	-22.5% (-73.7%, 56.3%)	42.4% (-48.5%, 82.9%)
BA.5	148 (15.0%)	836 (85.0%)	602 (21.0%)	2265 (79.0%)	0.666 (0.547, 0.811)	0.515 (0.409, 0.650)	33.4% (18.9%, 45.3%)	48.5% (35.0%, 59.1%)
14-30 days	29 (3.4%)	836 (96.6%)	115 (4.8%)	2265 (95.2%)	0.683 (0.451, 1.035)	0.568 (0.369, 0.876)	31.7% (-3.4%, 54.9%)	43.2% (12.4%, 63.1%)
31-90 days	112 (11.8%)	836 (88.2%)	466 (17.1%)	2265 (82.9%)	0.651 (0.522, 0.812)	0.502 (0.389, 0.647)	34.9% (18.8%, 47.8%)	49.8% (35.3%, 61.1%)
>90 days	7 (0.8%)	836 (99.2%)	21 (0.9%)	2265 (99.1%)	0.903 (0.383, 2.132)	0.516 (0.202, 1.320)	9.7% (-53.1%, 61.7%)	48.4% (-24.2%, 79.8%)

[a] When the OR or its 95% CI was >1, the rVE or its 95% CI was transformed as ([1/OR] – 1) x 100.

[b] Adjusted for age, sex, race/ethnicity, month of specimen collection, history of SARS-CoV-2 infection, history of SARS-CoV-2 molecular test, number of outpatient and virtual visits, medical center area, and time between third dose and specimen collection date.

[c] BA.2 excluding BA.2.12.1.

[d] Medical center area removed from adjustment set due to lack of model convergence.

CI, confidence interval; OR, odds ratio; rVE, relative vaccine effectiveness.

Supplementary Table 4a. Vaccine effectiveness of 3 doses of mRNA-1273 against COVID-19 hospitalization associated with SARS-CoV-2 variants

Subvariant	Test Positive		Test Negative		Odds Ratio (95% CI)		VE (95% CI)	
	3-dose Vaccinated (%)	Unvaccinated (%)	3-dose Vaccinated (%)	Unvaccinated (%)	Unadjusted	Adjusted[a]	Unadjusted	Adjusted[a]
BA.1	58 (16.0%)	304 (84.0%)	921 (82.5%)	195 (17.5%)	0.040 (0.029, 0.056)	0.025 (0.017, 0.037)	96.0% (94.4%, 97.1%)	97.5% (96.3%, 98.3%)
BA.2	46 (55.4%)	37 (44.6%)	183 (76.6%)	56 (23.4%)	0.380 (0.225, 0.644)	0.180 (0.092, 0.355)	62.0% (35.6%, 77.5%)	82.0% (64.5%, 90.8%)
BA.4/BA.5	15 (50.0%)	15 (50.0%)	65 (70.7%)	27 (29.3%)	0.415 (0.178, 0.967)	0.276 (0.100, 0.761)	58.5% (3.3%, 82.2%)	72.4% (23.9%, 90.0%)

[a] Adjusted for age, sex, race/ethnicity, month of specimen collection, history of SARS-CoV-2 infection, history of SARS-CoV-2 molecular test, and number of outpatient and virtual visits. Medical center area dropped from adjustment set due to lack of model convergence.
CI, confidence interval; VE, vaccine effectiveness.

Supplementary Table 4b. Relative vaccine effectiveness of 3 versus 2 doses of mRNA-1273 against COVID-19 hospitalization associated with SARS-CoV-2 variants

Subvariant	Test Positive		Test Negative		Odds Ratio (95% CI)		rVE (95% CI)	
	3-dose Vaccinated (%)	2-dose Vaccinated (%)	3-dose Vaccinated (%)	2-dose Vaccinated (%)	Unadjusted	Adjusted[a]	Unadjusted	Adjusted[a]
BA.1	58 (36.0%)	103 (64.0%)	921 (76.8%)	278 (23.2%)	0.170 (0.120, 0.241)	0.112 (0.075, 0.167)	83.0% (75.9%, 88.0%)	88.8% (83.3%, 92.5%)
BA.2	46 (61.3%)	29 (38.7%)	183 (81.7%)	41 (18.3%)	0.355 (0.200, 0.632)	0.250 (0.119, 0.524)	64.5% (36.8%, 80.0%)	75.0% (47.6%, 88.1%)
BA.4/BA.5	15 (57.7%)	11 (42.3%)	65 (85.5%)	11 (14.5%)	0.231 (0.084, 0.632)	0.125 (0.032, 0.482)	76.9% (36.8%, 91.6%)	87.5% (51.8%, 96.8%)

[a] Adjusted for age, sex, race/ethnicity, month of specimen collection, history of SARS-CoV-2 infection, history of SARS-CoV-2 molecular test, number of outpatient and virtual visits, and time between second dose and specimen collection date. Medical center area dropped from adjustment set due to lack of model convergence.

CI, confidence interval; rVE, relative vaccine effectiveness.

Supplementary Table 5a. Vaccine effectiveness of 4 doses of mRNA-1273 against COVID-19 hospitalization associated with SARS-CoV-2 variants

	Test Positive		Test Negative		Odds Ratio (95% CI)		VE (95% CI)	
Subvariant	4-dose Vaccinated (%)	Unvaccinated (%)	4-dose Vaccinated (%)	Unvaccinated (%)	Unadjusted	Adjusted[a]	Unadjusted	Adjusted[a]
BA.2	6 (14.0%)	37 (86.0%)	73 (56.6%)	56 (43.4%)	0.124 (0.049, 0.315)	0.036 (0.011, 0.116)	87.6% (68.5%, 95.1%)	96.4% (88.4%, 98.9%)
BA.4/BA.5	5 (25.0%)	15 (75.0%)	34 (55.7%)	27 (44.3%)	0.265 (0.085, 0.820)	0.115 (0.028, 0.482)	73.5% (18.0%, 91.5%)	88.5% (51.8%, 97.2%)

[a] Adjusted for age, sex, race/ethnicity, month of specimen collection, history of SARS-CoV-2 infection, history of SARS-CoV-2 molecular test, and number of outpatient and virtual visits. Medical center area dropped from adjustment set due to lack of model convergence.

CI, confidence interval; VE, vaccine effectiveness.

Supplementary Table 5b. Relative vaccine effectiveness of 4 versus 3 doses of mRNA-1273 against COVID-19 hospitalization associated with SARS-CoV-2 variants

Subvariant	Test Positive		Test Negative		Odds Ratio (95% CI)		rVE (95% CI)[a]	
	4-dose Vaccinated (%)	3-dose Vaccinated (%)	4-dose Vaccinated (%)	3-dose Vaccinated (%)	Unadjusted	Adjusted[b]	Unadjusted	Adjusted[b]
BA.2	6 (11.5%)	46 (88.5%)	73 (28.5%)	183 (71.5%)	0.327 (0.134, 0.799)	0.145 (0.051, 0.413)	67.3% (20.1%, 86.6%)	85.5% (58.7%, 94.9%)
BA.4/BA.5	5 (25.0%)	15 (75.0%)	34 (34.3%)	65 (65.7%)	0.637 (0.213, 1.903)	0.278 (0.071, 1.080)	36.3% (-47.4%, 78.7%)	72.2% (-7.4%, 92.9%)

[a] When the OR or its 95% CI was >1, the rVE or its 95% CI was transformed as ([1/OR] – 1) x 100.

[b] Adjusted for age, sex, race/ethnicity, month of specimen collection, history of SARS-CoV-2 molecular test, number of outpatient and virtual visits, and time between third dose and specimen collection date. Medical center area and history of SARS-CoV-2 infection dropped from adjustment set due to lack of model convergence.

CI, confidence interval; OR, odds ratio; rVE, relative vaccine effectiveness.

Supplementary Table 6a. Vaccine effectiveness of 3 doses of mRNA-1273 against infection with SARS-CoV-2 variants by time since vaccination, using SGTF data to impute unidentified subvariants

Subvariant/Time since 3rd dose	Test Positive		Test Negative		Odds Ratio (95% CI)		VE (95% CI)[a]	
	3-dose Vaccinated (%)	Unvaccinated (%)	3-dose Vaccinated (%)	Unvaccinated (%)	Unadjusted	Adjusted[b]	Unadjusted	Adjusted[b]
BA.1	2838 (39.0%)	4435 (61.0%)	15080 (64.3%)	8366 (35.7%)	0.355 (0.336, 0.375)	0.306 (0.287, 0.326)	64.5% (62.5%, 66.4%)	69.4% (67.4%, 71.3%)
14-30 days	286 (6.1%)	4435 (93.9%)	2613 (23.8%)	8366 (76.2%)	0.207 (0.182, 0.235)	0.204 (0.179, 0.233)	79.3% (76.5%, 81.8%)	79.6% (76.7%, 82.1%)
31-90 days	1730 (28.1%)	4435 (71.9%)	9537 (53.3%)	8366 (46.7%)	0.342 (0.321, 0.364)	0.308 (0.286, 0.330)	65.8% (63.6%, 67.9%)	69.2% (67.0%, 71.4%)
91-150 days	715 (13.9%)	4435 (86.1%)	2646 (24.0%)	8366 (76.0%)	0.510 (0.466, 0.558)	0.404 (0.363, 0.449)	49.0% (44.2%, 53.4%)	59.6% (55.1%, 63.7%)
>150 days	107 (2.4%)	4435 (97.6%)	284 (3.3%)	8366 (96.7%)	0.711 (0.567, 0.890)	0.537 (0.422, 0.683)	28.9% (11.0%, 43.3%)	46.3% (31.7%, 57.8%)
BA.2	7270 (68.5%)	3345 (31.5%)	19979 (63.9%)	11275 (36.1%)	1.227 (1.170, 1.286)	1.083 (1.027, 1.142)	-18.5% (-22.2%, -14.5%)	-7.6% (-12.4%, -2.6%)
14-30 days	29 (0.9%)	3345 (99.1%)	256 (2.2%)	11275 (97.8%)	0.382 (0.260, 0.562)	0.355 (0.240, 0.526)	61.8% (43.8%, 74.0%)	64.5% (47.4%, 76.0%)
31-90 days	339 (9.2%)	3345 (90.8%)	1971 (14.9%)	11275 (85.1%)	0.580 (0.513, 0.655)	0.607 (0.535, 0.689)	42.0% (34.5%, 48.7%)	39.3% (31.1%, 46.5%)
91-150 days	2442 (42.2%)	3345 (57.8%)	8316 (42.4%)	11275 (57.6%)	0.990 (0.933, 1.050)	0.939 (0.880, 1.001)	1.0% (-4.8%, 6.7%)	6.1% (-0.1%, 12.0%)
>150 days	4460 (57.1%)	3345 (42.9%)	9436 (45.6%)	11275 (54.4%)	1.593 (1.512, 1.679)	1.356 (1.276, 1.440)	-37.2% (-40.4%, -33.8%)	-26.2% (-30.6%, -21.7%)
BA.4/BA.5	1874 (65.5%)	986 (34.5%)	5067 (62.5%)	3043 (37.5%)	1.141 (1.044, 1.248)	1.065 (0.964, 1.177)	-12.4% (-19.9%, -4.2%)	-6.1% (-15.0%, 3.6%)
14-30 days	4 (0.4%)	986 (99.6%)	68 (2.2%)	3043 (97.8%)	0.182 (0.066, 0.499)	0.182 (0.066, 0.503)	81.8% (50.1%, 93.4%)	81.8% (49.7%, 93.4%)
31-90 days	40 (3.9%)	986 (96.1%)	226 (6.9%)	3043 (93.1%)	0.546 (0.387, 0.770)	0.554 (0.390, 0.786)	45.4% (23.0%, 61.3%)	44.6% (21.4%, 61.0%)
91-150 days	277 (21.9%)	986 (78.1%)	1041 (25.5%)	3043 (74.5%)	0.821 (0.706, 0.955)	0.849 (0.725, 0.994)	17.9% (4.5%, 29.4%)	15.1% (0.6%, 27.5%)
>150 days	1553 (61.2%)	986 (38.8%)	3732 (55.1%)	3043 (44.9%)	1.284 (1.170, 1.410)	1.180 (1.063, 1.310)	-22.1% (-29.1%, -14.5%)	-15.3% (-23.7%, -5.9%)

[a] When the OR or its 95% CI was >1, the VE or its 95% CI was transformed as ([1/OR] – 1) x 100.

[b] Adjusted for age, sex, race/ethnicity, month of specimen collection, history of SARS-CoV-2 infection, history of SARS-CoV-2 molecular test, number of outpatient and virtual visits, and medical center area.
CI, confidence interval; OR, odds ratio; SGTF, S gene target failure; VE, vaccine effectiveness.

Supplementary Table 6b. Relative vaccine effectiveness of 3 versus 2 doses of mRNA-1273 against infection with SARS-CoV-2 variants by time since vaccination, using SGTF data to impute unidentified subvariants

Subvariant/Time since 3rd dose	Test Positive		Test Negative		Odds Ratio (95% CI)		rVE (95% CI)[a]	
	3-dose Vaccinated (%)	2-dose Vaccinated (%)	3-dose Vaccinated (%)	2-dose Vaccinated (%)	Unadjusted	Adjusted[b]	Unadjusted	Adjusted[b]
BA.1	2838 (46.3%)	3296 (53.7%)	15080 (64.7%)	8237 (35.3%)	0.470 (0.444, 0.498)	0.373 (0.348, 0.399)	53.0% (50.2%, 55.6%)	62.7% (60.1%, 65.2%)
14-30 days	286 (8.0%)	3296 (92.0%)	2613 (24.1%)	8237 (75.9%)	0.274 (0.241, 0.311)	0.260 (0.228, 0.297)	72.6% (68.9%, 75.9%)	74.0% (70.3%, 77.2%)
31-90 days	1730 (34.4%)	3296 (65.6%)	9537 (53.7%)	8237 (46.3%)	0.453 (0.425, 0.484)	0.387 (0.359, 0.417)	54.7% (51.6%, 57.5%)	61.3% (58.3%, 64.1%)
91-150 days	715 (17.8%)	3296 (82.2%)	2646 (24.3%)	8237 (75.7%)	0.675 (0.616, 0.740)	0.468 (0.419, 0.524)	32.5% (26.0%, 38.4%)	53.2% (47.6%, 58.1%)
>150 days	107 (3.1%)	3296 (96.9%)	284 (3.3%)	8237 (96.7%)	0.942 (0.751, 1.180)	0.607 (0.475, 0.775)	5.8% (-15.3%, 24.9%)	39.3% (22.5%, 52.5%)
BA.2	7270 (74.9%)	2434 (25.1%)	19979 (73.7%)	7142 (26.3%)	1.068 (1.012, 1.126)	0.912 (0.858, 0.970)	-6.3% (-11.2%, -1.2%)	8.8% (3.0%, 14.2%)
14-30 days	29 (1.2%)	2434 (98.8%)	256 (3.5%)	7142 (96.5%)	0.333 (0.226, 0.490)	0.320 (0.216, 0.475)	66.7% (51.0%, 77.4%)	68.0% (52.5%, 78.4%)
31-90 days	339 (12.2%)	2434 (87.8%)	1971 (21.6%)	7142 (78.4%)	0.505 (0.446, 0.571)	0.544 (0.477, 0.620)	49.5% (42.9%, 55.4%)	45.6% (38.0%, 52.3%)
91-150 days	2442 (50.1%)	2434 (49.9%)	8316 (53.8%)	7142 (46.2%)	0.862 (0.808, 0.919)	0.817 (0.761, 0.877)	13.8% (8.1%, 19.2%)	18.3% (12.3%, 23.9%)
>150 days	4460 (64.7%)	2434 (35.3%)	9436 (56.9%)	7142 (43.1%)	1.387 (1.309, 1.470)	1.159 (1.081, 1.242)	-27.9% (-32.0%, -23.6%)	-13.7% (-19.5%, -7.5%)
BA.4/BA.5[c]	1874 (75.7%)	602 (24.3%)	5067 (72.3%)	1938 (27.7%)	1.191 (1.071, 1.323)	1.046 (0.929, 1.177)	-16.0% (-24.4%, -6.6%)	-4.4% (-15.0%, 7.1%)
14-30 days	4 (0.7%)	602 (99.3%)	68 (3.4%)	1938 (96.6%)	0.189 (0.069, 0.521)	0.193 (0.070, 0.535)	81.1% (47.9%, 93.1%)	80.7% (46.5%, 93.0%)
31-90 days	40 (6.2%)	602 (93.8%)	226 (10.4%)	1938 (89.6%)	0.570 (0.402, 0.807)	0.594 (0.417, 0.847)	43.0% (19.3%, 59.8%)	40.6% (15.3%, 58.3%)
91-150 days	277 (31.5%)	602 (68.5%)	1041 (34.9%)	1938 (65.1%)	0.857 (0.729, 1.006)	0.871 (0.736, 1.030)	14.3% (-0.6%, 27.1%)	12.9% (-3.0%, 26.4%)
>150 days	1553 (72.1%)	602 (27.9%)	3732 (65.8%)	1938 (34.2%)	1.340 (1.201, 1.494)	1.174 (1.036, 1.330)	-25.4% (-33.1%, -16.8%)	-14.8% (-24.8%, -3.5%)

[a] When the OR or its 95% CI was >1, the rVE or its 95% CI was transformed as ([1/OR] – 1) x 100.

[b] Adjusted for age, sex, race/ethnicity, month of specimen collection, history of SARS-CoV-2 infection, history of SARS-CoV-2 molecular test, number of outpatient and virtual visits, medical center area, and time between second dose and specimen collection date.

[c] Medical center area dropped from adjustment set due to lack of model convergence.

CI, confidence interval; OR, odds ratio; SGTF, S gene target failure; rVE, relative vaccine effectiveness.

Supplementary Table 7a. Vaccine effectiveness of 4 doses of mRNA-1273 against infection with SARS-CoV-2 variants by time since vaccination, using SGTF data to impute unidentified subvariants

Subvariant/Time since 4th dose	Test Positive		Test Negative		Odds Ratio (95% CI)		VE (95% CI)[a]	
	4-dose Vaccinated (%)	Unvaccinated (%)	4-dose Vaccinated (%)	Unvaccinated (%)	Unadjusted	Adjusted[b]	Unadjusted	Adjusted[b]
BA.2[c]	606 (15.3%)	3345 (84.7%)	2525 (18.3%)	11275 (81.7%)	0.809 (0.734, 0.891)	0.575 (0.507, 0.652)	19.1% (10.9%, 26.6%)	42.5% (34.8%, 49.3%)
14-30 days	184 (5.2%)	3345 (94.8%)	1013 (8.2%)	11275 (91.8%)	0.612 (0.521, 0.720)	0.441 (0.368, 0.529)	38.8% (28.0%, 47.9%)	55.9% (47.1%, 63.2%)
31-90 days	393 (10.5%)	3345 (89.5%)	1428 (11.2%)	11275 (88.8%)	0.928 (0.824, 1.044)	0.646 (0.559, 0.747)	7.2% (-4.2%, 17.6%)	35.4% (25.3%, 44.1%)
>90 days	29 (0.9%)	3345 (99.1%)	84 (0.7%)	11275 (99.3%)	1.164 (0.762, 1.778)	0.927 (0.600, 1.434)	-14.1% (-43.8%, 23.8%)	7.3% (-30.2%, 40.0%)
BA.4/BA.5[c]	299 (23.3%)	986 (76.7%)	1218 (28.6%)	3043 (71.4%)	0.758 (0.655, 0.876)	0.579 (0.475, 0.706)	24.2% (12.4%, 34.5%)	42.1% (29.4%, 52.5%)
14-30 days	50 (4.8%)	986 (95.2%)	272 (8.2%)	3043 (91.8%)	0.567 (0.416, 0.774)	0.461 (0.329, 0.646)	43.3% (22.6%, 58.4%)	53.9% (35.4%, 67.1%)
31-90 days	234 (19.2%)	986 (80.8%)	897 (22.8%)	3043 (77.2%)	0.805 (0.686, 0.946)	0.607 (0.490, 0.751)	19.5% (5.4%, 31.4%)	39.3% (24.9%, 51.0%)
>90 days	15 (1.5%)	986 (98.5%)	49 (1.6%)	3043 (98.4%)	0.945 (0.527, 1.692)	0.757 (0.414, 1.383)	5.5% (-40.9%, 47.3%)	24.3% (-27.7%, 58.6%)

[a] When the OR or its 95% CI was >1, the VE or its 95% CI was transformed as ([1/OR] – 1) x 100.

[b] Adjusted for age, sex, race/ethnicity, month of specimen collection, history of SARS-CoV-2 infection, history of SARS-CoV-2 molecular test, number of outpatient and virtual visits, and medical center area.
[c] Medical center area dropped from adjustment set due to lack of model convergence.
CI, confidence interval; OR, odds ratio; SGTF, S gene target failure; VE, vaccine effectiveness.

Supplementary Table 7b. Relative vaccine effectiveness of 4 versus 3 doses of mRNA-1273 against infection with SARS-CoV-2 variants by time since vaccination, using SGTF data to impute unidentified subvariants

Subvariant/Time since 4th dose	Test Positive		Test Negative		Odds Ratio (95% CI)		rVE (95% CI)[a]	
	4-dose Vaccinated (%)	3-dose Vaccinated (%)	4-dose Vaccinated (%)	3-dose Vaccinated (%)	Unadjusted	Adjusted[b]	Unadjusted	Adjusted[b]
BA.2	606 (7.7%)	7270 (92.3%)	2525 (11.2%)	19979 (88.8%)	0.660 (0.601, 0.724)	0.457 (0.411, 0.508)	34.0% (27.6%, 39.9%)	54.3% (49.2%, 58.9%)
14-30 days	184 (2.5%)	7270 (97.5%)	1013 (4.8%)	19979 (95.2%)	0.499 (0.426, 0.585)	0.379 (0.321, 0.448)	50.1% (41.5%, 57.4%)	62.1% (55.2%, 67.9%)
31-90 days	393 (5.1%)	7270 (94.9%)	1428 (6.7%)	19979 (93.3%)	0.756 (0.674, 0.848)	0.517 (0.455, 0.587)	24.4% (15.2%, 32.6%)	48.3% (41.3%, 54.5%)
>90 days	29 (0.4%)	7270 (99.6%)	84 (0.4%)	19979 (99.6%)	0.949 (0.622, 1.448)	0.379 (0.241, 0.596)	5.1% (-31.0%, 37.8%)	62.1% (40.4%, 75.9%)
BA.4/BA.5[c]	299 (13.8%)	1874 (86.2%)	1218 (19.4%)	5067 (80.6%)	0.664 (0.579, 0.762)	0.537 (0.458, 0.631)	33.6% (23.8%, 42.1%)	46.3% (36.9%, 54.2%)
14-30 days	50 (2.6%)	1874 (97.4%)	272 (5.1%)	5067 (94.9%)	0.497 (0.366, 0.675)	0.445 (0.324, 0.611)	50.3% (32.5%, 63.4%)	55.5% (38.9%, 67.6%)
31-90 days	234 (11.1%)	1874 (88.9%)	897 (15.0%)	5067 (85.0%)	0.705 (0.605, 0.822)	0.568 (0.476, 0.678)	29.5% (17.8%, 39.5%)	43.2% (32.2%, 52.4%)
>90 days	15 (0.8%)	1874 (99.2%)	49 (1.0%)	5067 (99.0%)	0.828 (0.463, 1.479)	0.489 (0.264, 0.907)	17.2% (-32.4%, 53.7%)	51.1% (9.3%, 73.6%)

[a] When the OR or its 95% CI was >1, the rVE or its 95% CI was transformed as ([1/OR] – 1) x 100.

[b] Adjusted for age, sex, race/ethnicity, month of specimen collection, history of SARS-CoV-2 infection, history of SARS-CoV-2 molecular test, number of outpatient and virtual visits, medical center area, and time between third dose and specimen collection date.

[c] Medical center area dropped from adjustment set due to lack of model convergence.

CI, confidence interval; OR, odds ratio; SGTF, S gene target failure; rVE, relative vaccine effectiveness.

Supplementary Table 8a. Vaccine effectiveness of 3 doses of mRNA-1273 against COVID-19 hospitalization associated with SARS-CoV-2 variants, using SGTF data to impute unidentified subvariants

Subvariant	Test Positive		Test Negative		Odds Ratio (95% CI)		VE (95% CI)[a]	
	3-dose Vaccinated (%)	Unvaccinated (%)	3-dose Vaccinated (%)	Unvaccinated (%)	Unadjusted	Adjusted[b]	Unadjusted	Adjusted[b]
BA.1	61 (16.4%)	310 (83.6%)	941 (82.3%)	202 (17.7%)	0.042 (0.031, 0.058)	0.027 (0.019, 0.040)	95.8% (94.2%, 96.9%)	97.3% (96.0%, 98.1%)
BA.2	49 (56.3%)	38 (43.7%)	197 (77.9%)	56 (22.1%)	0.367 (0.219, 0.615)	0.174 (0.089, 0.339)	63.3% (38.5%, 78.1%)	82.6% (66.1%, 91.1%)
BA.4/BA.5	16 (51.6%)	15 (48.4%)	67 (69.8%)	29 (30.2%)	0.462 (0.202, 1.057)	0.314 (0.117, 0.844)	53.8% (-5.4%, 79.8%)	68.6% (15.6%, 88.3%)

[a] When the OR or its 95% CI was >1, the VE or its 95% CI was transformed as ([1/OR] – 1) x 100.

[b] Adjusted for age, sex, race/ethnicity, month of specimen collection, history of SARS-CoV-2 infection, history of SARS-CoV-2 molecular test, and number of outpatient and virtual visits. Medical center area dropped from adjustment set due to lack of model convergence.

CI, confidence interval; OR, odds ratio; SGTF, S gene target failure; VE, vaccine effectiveness.

Supplementary Table 8b. Relative vaccine effectiveness of 3 versus 2 doses of mRNA-1273 against COVID-19 hospitalization associated with SARS-CoV-2 variants, using SGTF data to impute unidentified subvariants

Subvariant	Test Positive		Test Negative		Odds Ratio (95% CI)		rVE (95% CI)	
	3-dose Vaccinated (%)	2-dose Vaccinated (%)	3-dose Vaccinated (%)	2-dose Vaccinated (%)	Unadjusted	Adjusted[a]	Unadjusted	Adjusted[a]
BA.1	61 (36.3%)	107 (63.7%)	941 (76.5%)	289 (23.5%)	0.175 (0.124, 0.246)	0.114 (0.077, 0.169)	82.5% (75.4%, 87.6%)	88.6% (83.1%, 92.3%)
BA.2	49 (62.8%)	29 (37.2%)	197 (81.4%)	45 (18.6%)	0.386 (0.220, 0.677)	0.268 (0.130, 0.551)	61.4% (32.3%, 78.0%)	73.2% (44.9%, 87.0%)
BA.4/BA.5	16 (57.1%)	12 (42.9%)	67 (85.9%)	11 (14.1%)	0.219 (0.082, 0.585)	0.118 (0.032, 0.432)	78.1% (41.5%, 91.8%)	88.2% (56.8%, 96.8%)

[a] Adjusted for age, sex, race/ethnicity, month of specimen collection, history of SARS-CoV-2 infection, history of SARS-CoV-2 molecular test, number of outpatient and virtual visits, and time between second dose and specimen collection date. Medical center area dropped from adjustment set due to lack of model convergence.

CI, confidence interval; SGTF, S gene target failure; rVE, relative vaccine effectiveness.

Supplementary Table 9a. Vaccine effectiveness of 4 doses of mRNA-1273 against COVID-19 hospitalization associated with SARS-CoV-2 variants, using SGTF data to impute unidentified subvariants

Subvariant	Test Positive		Test Negative		Odds Ratio (95% CI)		VE (95% CI)	
	4-dose Vaccinated (%)	Unvaccinated (%)	4-dose Vaccinated (%)	Unvaccinated (%)	Unadjusted	Adjusted[a]	Unadjusted	Adjusted[a]
BA.2	9 (19.1%)	38 (80.9%)	76 (57.6%)	56 (42.4%)	0.175 (0.078, 0.390)	0.059 (0.021, 0.162)	82.5% (61.0%, 92.2%)	94.1% (83.8%, 97.9%)
BA.4/BA.5	5 (25.0%)	15 (75.0%)	36 (55.4%)	29 (44.6%)	0.269 (0.087, 0.826)	0.110 (0.026, 0.458)	73.1% (17.4%, 91.3%)	89.0% (54.2%, 97.4%)

[a] Adjusted for age, sex, race/ethnicity, month of specimen collection, history of SARS-CoV-2 infection, history of SARS-CoV-2 molecular test, and number of outpatient and virtual visits. Medical center area dropped from adjustment set due to lack of model convergence.

CI, confidence interval; SGTF, S gene target failure; VE, vaccine effectiveness.

Supplementary Table 9b. Relative vaccine effectiveness of 4 versus 3 doses of mRNA-1273 against COVID-19 hospitalization associated with SARS-CoV-2 variants, using SGTF data to impute unidentified subvariants

Subvariant	Test Positive		Test Negative		Odds Ratio (95% CI)		rVE (95% CI)[a]	
	4-dose Vaccinated (%)	3-dose Vaccinated (%)	4-dose Vaccinated (%)	3-dose Vaccinated (%)	Unadjusted	Adjusted[b]	Unadjusted	Adjusted[b]
BA.2	9 (15.5%)	49 (84.5%)	76 (27.8%)	197 (72.2%)	0.476 (0.223, 1.016)	0.246 (0.102, 0.595)	52.4% (-1.6%, 77.7%)	75.4% (40.5%, 89.8%)
BA.4/BA.5	5 (23.8%)	16 (76.2%)	36 (35.0%)	67 (65.0%)	0.582 (0.197, 1.717)	0.243 (0.063, 0.938)	41.8% (-41.8%, 80.3%)	75.7% (6.2%, 93.7%)

[a] When the OR or its 95% CI was >1, the rVE or its 95% CI was transformed as ([1/OR] – 1) x 100.

[b] Adjusted for age, sex, race/ethnicity, month of specimen collection, history of SARS-CoV-2 molecular test, number of outpatient and virtual visits, and time between third dose and specimen collection date. Medical center area and history of SARS-CoV-2 infection dropped from adjustment set due to lack of model convergence.

CI, confidence interval; OR, odds ratio; SGTF, S gene target failure; rVE, relative vaccine effectiveness.

Supplementary Table 10a. Vaccine effectiveness of 3 doses of mRNA-1273 against infection with SARS-CoV-2 variants by time since vaccination, excluding immunocompromised patients

Subvariant/Time since 3rd dose	Test Positive		Test Negative		Odds Ratio (95% CI)		VE (95% CI)[a]	
	3-dose Vaccinated (%)	Unvaccinated (%)	3-dose Vaccinated (%)	Unvaccinated (%)	Unadjusted	Adjusted[b]	Unadjusted	Adjusted[b]
BA.1	1269 (34.7%)	2392 (65.3%)	7893 (65.5%)	4162 (34.5%)	0.280 (0.259, 0.302)	0.230 (0.210, 0.252)	72.0% (69.8%, 74.1%)	77.0% (74.8%, 79.0%)
14-30 days	118 (4.7%)	2392 (95.3%)	1419 (25.4%)	4162 (74.6%)	0.145 (0.119, 0.176)	0.138 (0.113, 0.169)	85.5% (82.4%, 88.1%)	86.2% (83.1%, 88.7%)
31-90 days	820 (25.5%)	2392 (74.5%)	5183 (55.5%)	4162 (44.5%)	0.275 (0.252, 0.301)	0.236 (0.213, 0.261)	72.5% (69.9%, 74.8%)	76.4% (73.9%, 78.7%)
91-150 days	301 (11.2%)	2392 (88.8%)	1201 (22.4%)	4162 (77.6%)	0.436 (0.381, 0.500)	0.324 (0.276, 0.379)	56.4% (50.0%, 61.9%)	67.6% (62.1%, 72.4%)
>150 days	30 (1.2%)	2392 (98.8%)	90 (2.1%)	4162 (97.9%)	0.580 (0.383, 0.879)	0.397 (0.255, 0.617)	42.0% (12.1%, 61.7%)	60.3% (38.3%, 74.5%)
BA.2[c,d]	2355 (66.9%)	1164 (33.1%)	6623 (64.4%)	3666 (35.6%)	1.120 (1.033, 1.214)	1.007 (0.920, 1.102)	-10.7% (-17.7%, -3.2%)	-0.7% (-9.2%, 8.0%)
14-30 days	12 (1.0%)	1164 (99.0%)	88 (2.3%)	3666 (97.7%)	0.430 (0.234, 0.788)	0.403 (0.217, 0.750)	57.0% (21.2%, 76.6%)	59.7% (25.0%, 78.3%)
31-90 days	136 (10.5%)	1164 (89.5%)	771 (17.4%)	3666 (82.6%)	0.556 (0.458, 0.674)	0.570 (0.466, 0.699)	44.4% (32.6%, 54.2%)	43.0% (30.1%, 53.4%)
91-150 days	873 (42.9%)	1164 (57.1%)	3006 (45.1%)	3666 (54.9%)	0.915 (0.828, 1.011)	0.882 (0.792, 0.982)	8.5% (-1.1%, 17.2%)	11.8% (1.8%, 20.8%)
>150 days	1334 (53.4%)	1164 (46.6%)	2758 (42.9%)	3666 (57.1%)	1.523 (1.388, 1.672)	1.317 (1.184, 1.463)	-34.4% (-40.2%, -28.0%)	-24.0% (-31.7%, -15.6%)
BA.2.12.1[d]	1820 (70.1%)	776 (29.9%)	4746 (63.5%)	2728 (36.5%)	1.348 (1.224, 1.484)	1.132 (1.018, 1.258)	-25.8% (-32.6%, -18.3%)	-11.7% (-20.5%, -1.8%)
14-30 days	3 (0.4%)	776 (99.6%)	54 (1.9%)	2728 (98.1%)	0.195 (0.061, 0.626)	0.183 (0.056, 0.592)	80.5% (37.4%, 93.9%)	81.7% (40.8%, 94.4%)
31-90 days	51 (6.2%)	776 (93.8%)	298 (9.8%)	2728 (90.2%)	0.602 (0.442, 0.818)	0.636 (0.463, 0.872)	39.8% (18.2%, 55.8%)	36.4% (12.8%, 53.7%)
91-150 days	485 (38.5%)	776 (61.5%)	1706 (38.5%)	2728 (61.5%)	0.999 (0.879, 1.137)	0.896 (0.781, 1.028)	0.1% (-12.0%, 12.1%)	10.4% (-2.7%, 21.9%)
>150 days	1281 (62.3%)	776 (37.7%)	2688 (49.6%)	2728 (50.4%)	1.675 (1.510, 1.859)	1.369 (1.219, 1.537)	-40.3% (-46.2%, -33.8%)	-26.9% (-34.9%, -18.0%)
BA.4	297 (66.3%)	151 (33.7%)	752 (60.6%)	489 (39.4%)	1.279 (1.020, 1.604)	1.132 (0.874, 1.466)	-21.8% (-37.7%, -1.9%)	-11.6% (-31.8%, 12.6%)
14-30 days	1 (0.7%)	151 (99.3%)	10 (2.0%)	489 (98.0%)	0.324 (0.041, 2.551)	0.351 (0.043, 2.857)	67.6% (-60.8%, 95.9%)	64.9% (-65.0%, 95.7%)
31-90 days	10 (6.2%)	151 (93.8%)	28 (5.4%)	489 (94.6%)	1.157 (0.549, 2.436)	1.037 (0.475, 2.263)	-13.5% (-58.9%, 45.1%)	-3.6% (-55.8%, 52.5%)
91-150 days	43 (22.2%)	151 (77.8%)	174 (26.2%)	489 (73.8%)	0.800 (0.547, 1.171)	0.789 (0.525, 1.185)	20.0% (-14.6%, 45.3%)	21.1% (-15.6%, 47.5%)
>150 days	243 (61.7%)	151 (38.3%)	540 (52.5%)	489 (47.5%)	1.457 (1.150, 1.847)	1.261 (0.962, 1.653)	-31.4% (-45.9%, -13.0%)	-20.7% (-39.5%, 3.8%)
BA.5[d]	807 (65.6%)	424 (34.4%)	2181 (62.4%)	1316 (37.6%)	1.148 (1.002, 1.316)	1.069 (0.920, 1.241)	-12.9% (-24.0%, -0.2%)	-6.4% (-19.4%, 8.0%)
14-30 days	1 (0.2%)	424 (99.8%)	30 (2.2%)	1316 (97.8%)	0.104 (0.014, 0.761)	0.096 (0.013, 0.704)	89.6% (23.9%, 98.6%)	90.4% (29.6%, 98.7%)
31-90 days	14 (3.2%)	424 (96.8%)	109 (7.6%)	1316 (92.4%)	0.399 (0.226, 0.703)	0.399 (0.224, 0.708)	60.1% (29.7%, 77.4%)	60.1% (29.2%, 77.6%)
91-150 days	103 (19.5%)	424 (80.5%)	410 (23.8%)	1316 (76.2%)	0.780 (0.612, 0.993)	0.802 (0.624, 1.030)	22.0% (0.7%, 38.8%)	19.8% (-2.9%, 37.6%)
>150 days	689 (61.9%)	424 (38.1%)	1632 (55.4%)	1316 (44.6%)	1.310 (1.138, 1.509)	1.207 (1.032, 1.411)	-23.7% (-33.7%, -12.1%)	-17.1% (-29.1%, -3.1%)

[a] When the OR or its 95% CI was >1, the VE or its 95% CI was transformed as ([1/OR] – 1) x 100.

[b] Adjusted for age, sex, race/ethnicity, month of specimen collection, history of SARS-CoV-2 infection, history of SARS-CoV-2 molecular test, number of outpatient and virtual visits, and medical center area.

[c] BA.2 excluding BA.2.12.1.

[d] Medical center area removed from adjustment set due to lack of model convergence.

CI, confidence interval; OR, odds ratio; VE, vaccine effectiveness.

Supplementary Table 10b. Relative vaccine effectiveness of 3 versus 2 doses of mRNA-1273 against infection with SARS-CoV-2 variants by time since vaccination, excluding immunocompromised patients

Subvariant/Time since 3rd dose	Test Positive		Test Negative		Odds Ratio (95% CI)		rVE (95% CI)[a]	
	3-dose Vaccinated (%)	2-dose Vaccinated (%)	3-dose Vaccinated (%)	2-dose Vaccinated (%)	Unadjusted	Adjusted[b]	Unadjusted	Adjusted[b]
BA.1[c]	1269 (42.0%)	1749 (58.0%)	7893 (64.7%)	4298 (35.3%)	0.395 (0.364, 0.429)	0.292 (0.266, 0.322)	60.5% (57.1%, 63.6%)	70.8% (67.8%, 73.4%)
14-30 days	118 (6.3%)	1749 (93.7%)	1419 (24.8%)	4298 (75.2%)	0.204 (0.168, 0.249)	0.185 (0.151, 0.226)	79.6% (75.1%, 83.2%)	81.5% (77.4%, 84.9%)
31-90 days	820 (31.9%)	1749 (68.1%)	5183 (54.7%)	4298 (45.3%)	0.389 (0.355, 0.426)	0.311 (0.280, 0.346)	61.1% (57.4%, 64.5%)	68.9% (65.4%, 72.0%)
91-150 days	301 (14.7%)	1749 (85.3%)	1201 (21.8%)	4298 (78.2%)	0.616 (0.536, 0.707)	0.371 (0.314, 0.439)	38.4% (29.3%, 46.4%)	62.9% (56.1%, 68.6%)
>150 days	30 (1.7%)	1749 (98.3%)	90 (2.1%)	4298 (97.9%)	0.819 (0.540, 1.243)	0.425 (0.273, 0.664)	18.1% (-19.5%, 46.0%)	57.5% (33.6%, 72.7%)
BA.2[c,d]	2355 (74.0%)	827 (26.0%)	6623 (73.8%)	2349 (26.2%)	1.010 (0.921, 1.107)	0.843 (0.759, 0.937)	-1.0% (-9.7%, 7.9%)	15.7% (6.3%, 24.1%)
14-30 days	12 (1.4%)	827 (98.6%)	88 (3.6%)	2349 (96.4%)	0.387 (0.211, 0.712)	0.377 (0.202, 0.703)	61.3% (28.8%, 78.9%)	62.3% (29.7%, 79.8%)
31-90 days	136 (14.1%)	827 (85.9%)	771 (24.7%)	2349 (75.3%)	0.501 (0.411, 0.611)	0.497 (0.402, 0.613)	49.9% (38.9%, 58.9%)	50.3% (38.7%, 59.8%)
91-150 days	873 (51.4%)	827 (48.6%)	3006 (56.1%)	2349 (43.9%)	0.825 (0.739, 0.920)	0.759 (0.673, 0.856)	17.5% (8.0%, 26.1%)	24.1% (14.4%, 32.7%)
>150 days	1334 (61.7%)	827 (38.3%)	2758 (54.0%)	2349 (46.0%)	1.374 (1.240, 1.522)	1.134 (1.002, 1.282)	-27.2% (-34.3%, -19.3%)	-11.8% (-22.0%, -0.2%)
BA.2.12.1[c]	1820 (75.0%)	606 (25.0%)	4746 (73.4%)	1723 (26.6%)	1.090 (0.980, 1.214)	0.917 (0.811, 1.036)	-8.3% (-17.6%, 2.0%)	8.3% (-3.4%, 18.9%)
14-30 days	3 (0.5%)	606 (99.5%)	54 (3.0%)	1723 (97.0%)	0.158 (0.049, 0.507)	0.158 (0.049, 0.512)	84.2% (49.3%, 95.1%)	84.2% (48.8%, 95.1%)
31-90 days	51 (7.8%)	606 (92.2%)	298 (14.7%)	1723 (85.3%)	0.487 (0.356, 0.664)	0.561 (0.407, 0.774)	51.3% (33.6%, 64.4%)	43.9% (22.6%, 59.3%)
91-150 days	485 (44.5%)	606 (55.5%)	1706 (49.8%)	1723 (50.2%)	0.808 (0.705, 0.927)	0.758 (0.654, 0.880)	19.2% (7.3%, 29.5%)	24.2% (12.0%, 34.6%)
>150 days	1281 (67.9%)	606 (32.1%)	2688 (60.9%)	1723 (39.1%)	1.355 (1.209, 1.519)	1.121 (0.980, 1.282)	-26.2% (-34.1%, -17.3%)	-10.8% (-22.0%, 2.0%)
BA.4	297 (74.1%)	104 (25.9%)	752 (70.3%)	318 (29.7%)	1.208 (0.932, 1.565)	1.017 (0.756, 1.369)	-17.2% (-36.1%, 6.8%)	-1.7% (-26.9%, 24.4%)
14-30 days	1 (1.0%)	104 (99.0%)	10 (3.0%)	318 (97.0%)	0.306 (0.039, 2.418)	0.311 (0.038, 2.540)	69.4% (-58.6%, 96.1%)	68.9% (-60.6%, 96.2%)
31-90 days	10 (8.8%)	104 (91.2%)	28 (8.1%)	318 (91.9%)	1.092 (0.513, 2.324)	1.078 (0.493, 2.358)	-8.4% (-57.0%, 48.7%)	-7.2% (-57.6%, 50.7%)
91-150 days	43 (29.3%)	104 (70.7%)	174 (35.4%)	318 (64.6%)	0.756 (0.506, 1.128)	0.693 (0.453, 1.062)	24.4% (-11.3%, 49.4%)	30.7% (-5.8%, 54.7%)
>150 days	243 (70.0%)	104 (30.0%)	540 (62.9%)	318 (37.1%)	1.376 (1.052, 1.799)	1.165 (0.850, 1.597)	-27.3% (-44.4%, -5.0%)	-14.2% (-37.4%, 15.0%)
BA.5	807 (75.0%)	269 (25.0%)	2181 (72.6%)	822 (27.4%)	1.131 (0.964, 1.326)	1.030 (0.858, 1.237)	-11.6% (-24.6%, 3.6%)	-2.9% (-19.1%, 14.2%)
14-30 days	1 (0.4%)	269 (99.6%)	30 (3.5%)	822 (96.5%)	0.102 (0.014, 0.751)	0.090 (0.012, 0.673)	89.8% (24.9%, 98.6%)	91.0% (32.7%, 98.8%)
31-90 days	14 (4.9%)	269 (95.1%)	109 (11.7%)	822 (88.3%)	0.392 (0.221, 0.696)	0.441 (0.245, 0.792)	60.8% (30.4%, 77.9%)	55.9% (20.8%, 75.5%)
91-150 days	103 (27.7%)	269 (72.3%)	410 (33.3%)	822 (66.7%)	0.768 (0.594, 0.992)	0.830 (0.633, 1.089)	23.2% (0.8%, 40.6%)	17.0% (-8.2%, 36.7%)
>150 days	689 (71.9%)	269 (28.1%)	1632 (66.5%)	822 (33.5%)	1.290 (1.095, 1.520)	1.186 (0.978, 1.438)	-22.5% (-34.2%, -8.7%)	-15.7% (-30.5%, 2.2%)

[a] When the OR or its 95% CI was >1, the rVE or its 95% CI was transformed as ([1/OR] – 1) x 100.

[b] Adjusted for age, sex, race/ethnicity, month of specimen collection, history of SARS-CoV-2 infection, history of SARS-CoV-2 molecular test, number of outpatient and virtual visits, medical center area, and time between second dose and specimen collection date.

[c] Medical center area removed from adjustment set due to lack of model convergence.

[d] BA.2 excluding BA.2.12.1.

CI, confidence interval; OR, odds ratio; rVE, relative vaccine effectiveness.

Supplementary Table 11a. Vaccine effectiveness of 4 doses of mRNA-1273 against infection with SARS-CoV-2 variants by time since vaccination, excluding immunocompromised patients

Subvariant/Time since 4th dose	Test Positive		Test Negative		Odds Ratio (95% CI)		VE (95% CI)[a]	
	4-dose Vaccinated (%)	Unvaccinated (%)	4-dose Vaccinated (%)	Unvaccinated (%)	Unadjusted	Adjusted[b]	Unadjusted	Adjusted[b]
BA.2[c]	140 (10.7%)	1164 (89.3%)	656 (15.2%)	3666 (84.8%)	0.672 (0.554, 0.816)	0.401 (0.314, 0.512)	32.8% (18.4%, 44.6%)	59.9% (48.8%, 68.6%)
14-30 days	52 (4.3%)	1164 (95.7%)	298 (7.5%)	3666 (92.5%)	0.550 (0.406, 0.743)	0.340 (0.242, 0.477)	45.0% (25.7%, 59.4%)	66.0% (52.3%, 75.8%)
31-90 days	84 (6.7%)	1164 (93.3%)	340 (8.5%)	3666 (91.5%)	0.778 (0.607, 0.997)	0.449 (0.334, 0.603)	22.2% (0.3%, 39.3%)	55.1% (39.7%, 66.6%)
>90 days	4 (0.3%)	1164 (99.7%)	18 (0.5%)	3666 (99.5%)	0.700 (0.236, 2.072)	0.490 (0.160, 1.505)	30.0% (-51.7%, 76.4%)	51.0% (-33.6%, 84.0%)
BA.2.12.1[d]	159 (17.0%)	776 (83.0%)	751 (21.6%)	2728 (78.4%)	0.744 (0.616, 0.899)	0.545 (0.427, 0.696)	25.6% (10.1%, 38.4%)	45.5% (30.4%, 57.3%)
14-30 days	38 (4.7%)	776 (95.3%)	281 (9.3%)	2728 (90.7%)	0.475 (0.336, 0.673)	0.357 (0.244, 0.524)	52.5% (32.7%, 66.4%)	64.3% (47.6%, 75.6%)
31-90 days	115 (12.9%)	776 (87.1%)	452 (14.2%)	2728 (85.8%)	0.894 (0.718, 1.114)	0.645 (0.490, 0.849)	10.6% (-10.2%, 28.2%)	35.5% (15.1%, 51.0%)
>90 days	6 (0.8%)	776 (99.2%)	18 (0.7%)	2728 (99.3%)	1.172 (0.464, 2.962)	0.906 (0.351, 2.341)	-14.7% (-66.2%, 53.6%)	9.4% (-57.3%, 64.9%)
BA.4[d]	32 (17.5%)	151 (82.5%)	173 (26.1%)	489 (73.9%)	0.599 (0.394, 0.911)	0.433 (0.254, 0.738)	40.1% (8.9%, 60.6%)	56.7% (26.2%, 74.6%)
14-30 days	4 (2.6%)	151 (97.4%)	50 (9.3%)	489 (90.7%)	0.259 (0.092, 0.729)	0.203 (0.068, 0.604)	74.1% (27.1%, 90.8%)	79.7% (39.6%, 93.2%)
31-90 days	25 (14.2%)	151 (85.8%)	117 (19.3%)	489 (80.7%)	0.692 (0.433, 1.106)	0.495 (0.273, 0.898)	30.8% (-9.6%, 56.7%)	50.5% (10.2%, 72.7%)
>90 days	3 (1.9%)	151 (98.1%)	6 (1.2%)	489 (98.8%)	1.619 (0.400, 6.552)	0.939 (0.219, 4.036)	-38.2% (-84.7%, 60.0%)	6.1% (-75.2%, 78.1%)
BA.5[d]	135 (24.2%)	424 (75.8%)	537 (29.0%)	1316 (71.0%)	0.780 (0.627, 0.971)	0.649 (0.481, 0.877)	22.0% (2.9%, 37.3%)	35.1% (12.3%, 51.9%)
14-30 days	27 (6.0%)	424 (94.0%)	108 (7.6%)	1316 (92.4%)	0.776 (0.502, 1.200)	0.670 (0.414, 1.085)	22.4% (-16.6%, 49.8%)	33.0% (-7.9%, 58.6%)
31-90 days	104 (19.7%)	424 (80.3%)	416 (24.0%)	1316 (76.0%)	0.776 (0.610, 0.988)	0.641 (0.465, 0.884)	22.4% (1.2%, 39.0%)	35.9% (11.6%, 53.5%)
>90 days	4 (0.9%)	424 (99.1%)	13 (1.0%)	1316 (99.0%)	0.955 (0.310, 2.945)	0.727 (0.228, 2.320)	4.5% (-66.0%, 69.0%)	27.3% (-56.9%, 77.2%)

[a] When the OR or its 95% CI was >1, the VE or its 95% CI was transformed as ([1/OR] – 1) x 100.

[b] Adjusted for age, sex, race/ethnicity, month of specimen collection, history of SARS-CoV-2 infection, history of SARS-CoV-2 molecular test, number of outpatient and virtual visits, and medical center area.
[c] BA.2 excluding BA.2.12.1.
[d] Medical center area removed from adjustment set due to lack of model convergence.
CI, confidence interval; OR, odds ratio; VE, vaccine effectiveness.

Supplementary Table 11b. Relative vaccine effectiveness of 4 versus 3 doses of mRNA-1273 against infection with SARS-CoV-2 variants by time since vaccination, excluding immunocompromised patients

Subvariant/Time since 4th dose	Test Positive		Test Negative		Odds Ratio (95% CI)		rVE (95% CI)[a]	
	4-dose Vaccinated (%)	3-dose Vaccinated (%)	4-dose Vaccinated (%)	3-dose Vaccinated (%)	Unadjusted	Adjusted[b]	Unadjusted	Adjusted[b]
BA.2[c,d]	140 (5.6%)	2355 (94.4%)	656 (9.0%)	6623 (91.0%)	0.600 (0.497, 0.725)	0.391 (0.318, 0.481)	40.0% (27.5%, 50.3%)	60.9% (51.9%, 68.2%)
14-30 days	52 (2.2%)	2355 (97.8%)	298 (4.3%)	6623 (95.7%)	0.491 (0.364, 0.661)	0.356 (0.261, 0.485)	50.9% (33.9%, 63.6%)	64.4% (51.5%, 73.9%)
31-90 days	84 (3.4%)	2355 (96.6%)	340 (4.9%)	6623 (95.1%)	0.695 (0.545, 0.886)	0.439 (0.337, 0.570)	30.5% (11.4%, 45.5%)	56.1% (43.0%, 66.3%)
>90 days	4 (0.2%)	2355 (99.8%)	18 (0.3%)	6623 (99.7%)	0.625 (0.211, 1.848)	0.154 (0.048, 0.499)	37.5% (-45.9%, 78.9%)	84.6% (50.1%, 95.2%)
BA.2.12.1[d]	159 (8.0%)	1820 (92.0%)	751 (13.7%)	4746 (86.3%)	0.552 (0.461, 0.661)	0.397 (0.324, 0.487)	44.8% (33.9%, 53.9%)	60.3% (51.3%, 67.6%)
14-30 days	38 (2.0%)	1820 (98.0%)	281 (5.6%)	4746 (94.4%)	0.353 (0.250, 0.497)	0.276 (0.194, 0.394)	64.7% (50.3%, 75.0%)	72.4% (60.6%, 80.6%)
31-90 days	115 (5.9%)	1820 (94.1%)	452 (8.7%)	4746 (91.3%)	0.663 (0.537, 0.820)	0.478 (0.378, 0.605)	33.7% (18.0%, 46.3%)	52.2% (39.5%, 62.2%)
>90 days	6 (0.3%)	1820 (99.7%)	18 (0.4%)	4746 (99.6%)	0.869 (0.344, 2.193)	0.286 (0.106, 0.773)	13.1% (-54.4%, 65.6%)	71.4% (22.7%, 89.4%)
BA.4	32 (9.7%)	297 (90.3%)	173 (18.7%)	752 (81.3%)	0.468 (0.314, 0.699)	0.340 (0.213, 0.544)	53.2% (30.1%, 68.6%)	66.0% (45.6%, 78.7%)
14-30 days	4 (1.3%)	297 (98.7%)	50 (6.2%)	752 (93.8%)	0.203 (0.073, 0.566)	0.151 (0.052, 0.441)	79.7% (43.4%, 92.7%)	84.9% (55.9%, 94.8%)
31-90 days	25 (7.8%)	297 (92.2%)	117 (13.5%)	752 (86.5%)	0.541 (0.344, 0.850)	0.426 (0.252, 0.718)	45.9% (15.0%, 65.6%)	57.4% (28.2%, 74.8%)
>90 days	3 (1.0%)	297 (99.0%)	6 (0.8%)	752 (99.2%)	1.266 (0.315, 5.095)	0.427 (0.087, 2.087)	-21.0% (-80.4%, 68.5%)	57.3% (-52.1%, 91.3%)
BA.5	135 (14.3%)	807 (85.7%)	537 (19.8%)	2181 (80.2%)	0.680 (0.553, 0.834)	0.515 (0.405, 0.656)	32.0% (16.6%, 44.7%)	48.5% (34.4%, 59.5%)
14-30 days	27 (3.2%)	807 (96.8%)	108 (4.7%)	2181 (95.3%)	0.676 (0.440, 1.038)	0.555 (0.354, 0.870)	32.4% (-3.7%, 56.0%)	44.5% (13.0%, 64.6%)
31-90 days	104 (11.4%)	807 (88.6%)	416 (16.0%)	2181 (84.0%)	0.676 (0.537, 0.850)	0.510 (0.391, 0.665)	32.4% (15.0%, 46.3%)	49.0% (33.5%, 60.9%)
>90 days	4 (0.5%)	807 (99.5%)	13 (0.6%)	2181 (99.4%)	0.832 (0.270, 2.558)	0.342 (0.100, 1.163)	16.8% (-60.9%, 73.0%)	65.8% (-14.0%, 90.0%)

[a] When the OR or its 95% CI was >1, the rVE or its 95% CI was transformed as ([1/OR] – 1) x 100.

[b] Adjusted for age, sex, race/ethnicity, month of specimen collection, history of SARS-CoV-2 infection, history of SARS-CoV-2 molecular test, number of outpatient and virtual visits, medical center area, and time between third dose and specimen collection date.

[c] BA.2 excluding BA.2.12.1.

[d] Medical center area removed from adjustment set due to lack of model convergence.

CI, confidence interval; OR, odds ratio; rVE, relative vaccine effectiveness.

Supplementary Table 12a. Vaccine effectiveness of 3 doses of mRNA-1273 against COVID-19 hospitalization associated with SARS-CoV-2 variants, excluding immunocompromised patients

Subvariant	Test Positive		Test Negative		Odds Ratio (95% CI)		VE (95% CI)	
	3-dose Vaccinated (%)	Unvaccinated (%)	3-dose Vaccinated (%)	Unvaccinated (%)	Unadjusted	Adjusted[a]	Unadjusted	Adjusted[a]
BA.1	25 (8.5%)	270 (91.5%)	847 (82.4%)	181 (17.6%)	0.020 (0.013, 0.031)	0.013 (0.008, 0.021)	98.0% (96.9%, 98.7%)	98.7% (97.9%, 99.2%)
BA.2	41 (57.7%)	30 (42.3%)	173 (77.2%)	51 (22.8%)	0.403 (0.229, 0.709)	0.174 (0.082, 0.369)	59.7% (29.1%, 77.1%)	82.6% (63.1%, 91.8%)
BA.4/BA.5	9 (37.5%)	15 (62.5%)	62 (69.7%)	27 (30.3%)	0.261 (0.102, 0.670)	0.115 (0.034, 0.390)	73.9% (33.0%, 89.8%)	88.5% (61.0%, 96.6%)

[a] Adjusted for age, sex, race/ethnicity, month of specimen collection, history of SARS-CoV-2 infection, history of SARS-CoV-2 molecular test, and number of outpatient and virtual visits. Medical center area dropped from adjustment set due to lack of model convergence.

CI, confidence interval; VE, vaccine effectiveness.

Supplementary Table 12b. Relative vaccine effectiveness of 3 versus 2 doses of mRNA-1273 against COVID-19 hospitalization associated with SARS-CoV-2 variants, excluding immunocompromised patients

Subvariant	Test Positive		Test Negative		Odds Ratio (95% CI)		rVE (95% CI)	
	3-dose Vaccinated (%)	2-dose Vaccinated (%)	3-dose Vaccinated (%)	2-dose Vaccinated (%)	Unadjusted	Adjusted[a]	Unadjusted	Adjusted[a]
BA.1	25 (22.9%)	84 (77.1%)	847 (76.6%)	259 (23.4%)	0.091 (0.057, 0.145)	0.062 (0.037, 0.103)	90.9% (85.5%, 94.3%)	93.8% (89.7%, 96.3%)
BA.2	41 (62.1%)	25 (37.9%)	173 (83.2%)	35 (16.8%)	0.332 (0.179, 0.614)	0.201 (0.089, 0.453)	66.8% (38.6%, 82.1%)	79.9% (54.7%, 91.1%)
BA.4/BA.5	9 (47.4%)	10 (52.6%)	62 (84.9%)	11 (15.1%)	0.160 (0.053, 0.482)	0.059 (0.011, 0.310)	84.0% (51.8%, 94.7%)	94.1% (69.0%, 98.9%)

[a] Adjusted for age, sex, race/ethnicity, month of specimen collection, history of SARS-CoV-2 infection, history of SARS-CoV-2 molecular test, number of outpatient and virtual visits, and time between second dose and specimen collection date. Medical center area dropped from adjustment set due to lack of model convergence.

CI, confidence interval; rVE, relative vaccine effectiveness.

Supplementary Table 13a. Vaccine effectiveness of 4 doses of mRNA-1273 against COVID-19 hospitalization associated with SARS-CoV-2 variants, excluding immunocompromised patients

Subvariant	Test Positive		Test Negative		Odds Ratio (95% CI)		VE (95% CI)[a]	
	4-dose Vaccinated (%)	Unvaccinated (%)	4-dose Vaccinated (%)	Unvaccinated (%)	Unadjusted	Adjusted[b]	Unadjusted	Adjusted[b]
BA.2[c]	4 (11.8%)	30 (88.2%)	64 (55.7%)	51 (44.3%)	0.106 (0.035, 0.321)	0.033 (0.009, 0.129)	89.4% (67.9%, 96.5%)	96.7% (87.1%, 99.1%)
BA.4/BA.5	5 (25.0%)	15 (75.0%)	28 (50.9%)	27 (49.1%)	0.321 (0.103, 1.007)	0.138 (0.033, 0.580)	67.9% (-0.7%, 89.7%)	86.2% (42.0%, 96.7%)

[a] When the OR or its 95% CI was >1, the VE or its 95% CI was transformed as ([1/OR] – 1) x 100.

[b] Adjusted for age, sex, race/ethnicity, month of specimen collection, history of SARS-CoV-2 infection, history of SARS-CoV-2 molecular test, and number of outpatient and virtual visits. Medical center area dropped from adjustment set due to lack of model convergence.

[c] History of SARS-CoV-2 infection dropped from adjustment set due to lack of model convergence.

CI, confidence interval; OR, odds ratio; VE, vaccine effectiveness.

Supplementary Table 13b. Relative vaccine effectiveness of 4 versus 3 doses of mRNA-1273 against COVID-19 hospitalization associated with SARS-CoV-2 variants, excluding immunocompromised patients

Subvariant	Test Positive		Test Negative		Odds Ratio (95% CI)		rVE (95% CI)[a]	
	4-dose Vaccinated (%)	3-dose Vaccinated (%)	4-dose Vaccinated (%)	3-dose Vaccinated (%)	Unadjusted	Adjusted[b]	Unadjusted	Adjusted[b]
BA.2	4 (8.9%)	41 (91.1%)	64 (27.0%)	173 (73.0%)	0.264 (0.091, 0.766)	0.140 (0.042, 0.467)	73.6% (23.4%, 90.9%)	86.0% (53.3%, 95.8%)
BA.4/BA.5[c]	5 (35.7%)	9 (64.3%)	28 (31.1%)	62 (68.9%)	1.230 (0.378, 4.007)	0.490 (0.115, 2.089)	-18.7% (-75.0%, 62.2%)	51.0% (-52.1%, 88.5%)

[a] When the OR or its 95% CI was >1, the rVE or its 95% CI was transformed as ([1/OR] – 1) x 100.

[b] Adjusted for age, sex, race/ethnicity, month of specimen collection, history of SARS-CoV-2 molecular test, number of outpatient and virtual visits, and time between third dose and specimen collection date. Medical center area and history of SARS-CoV-2 infection dropped from adjustment set due to lack of model convergence.

[c] Month of specimen collection dropped from adjustment set due to lack of model convergence.

CI, confidence interval; OR, odds ratio; rVE, relative vaccine effectiveness.

Supplementary Table 14. Frequency of SARS-CoV-2 positive cases among individuals with known history of SARS-CoV-2 infection, by vaccination status and variant

Frequency	BA.1	BA.2*	BA.2.12.1	BA.4	BA.5
**2-dose**	167	115	75	26	45
**3-dose**	86	211	184	38	97
**4-dose**	0	7	8	3	8
**Unvaccinated**	345	262	222	44	142

*BA.2 excluding BA.2.12.1

Note that 1-dose vaccinees and other variants (BA.3, Delta, recombinant, and unidentified lineages) were not included in the main analysis due to small sample size

Supplementary Table 15a. Vaccine effectiveness of 3 doses of mRNA-1273 against infection with SARS-CoV-2 variants by time since vaccination among those with known history of SARS-CoV-2 infection

	Test Positive		Test Negative		VE (95% CI)[a]	
Subvariant/Time since 3rd dose	3-dose Vaccinated (%)	Unvaccinated (%)	3-dose Vaccinated (%)	Unvaccinated (%)	Unadjusted	Adjusted[b]
BA.1	86 (20.0%)	345 (80.0%)	1002 (45.3%)	1212 (54.7%)	69.8% (61.3%, 76.5%)	71.7% (62.6%, 78.6%)
14-30 days	9 (2.5%)	345 (97.5%)	216 (15.1%)	1212 (84.9%)	85.4% (71.2%, 92.6%)	86.2% (72.5%, 93.1%)
31-90 days	57 (14.2%)	345 (85.8%)	670 (35.6%)	1212 (64.4%)	70.1% (59.8%, 77.8%)	71.4% (60.5%, 79.2%)
91-150 days	18 (5.0%)	345 (95.0%)	106 (8.0%)	1212 (92.0%)	40.3% (0.3%, 64.3%)	40.0% (-4.8%, 65.7%)
>150 days	2 (0.6%)	345 (99.4%)	10 (0.8%)	1212 (99.2%)	29.7% (-69.0%, 84.7%)	34.7% (-68.6%, 86.6%)
BA.2[c]	211 (44.6%)	262 (55.4%)	1199 (50.2%)	1189 (49.8%)	20.1% (2.6%, 34.5%)	24.3% (5.3%, 39.4%)
14-30 days	1 (0.4%)	262 (99.6%)	25 (2.1%)	1189 (97.9%)	81.8% (-25.7%, 97.6%)	83.3% (-21.1%, 97.8%)
31-90 days	21 (7.4%)	262 (92.6%)	194 (14.0%)	1189 (86.0%)	50.9% (21.4%, 69.3%)	37.4% (-1.9%, 61.6%)
91-150 days	84 (24.3%)	262 (75.7%)	590 (33.2%)	1189 (66.8%)	35.4% (15.8%, 50.4%)	35.6% (14.5%, 51.4%)
>150 days	105 (28.6%)	262 (71.4%)	390 (24.7%)	1189 (75.3%)	-18.2% (-36.5%, 5.2%)	-4.6% (-28.8%, 21.7%)
BA.2.12.1	184 (45.3%)	222 (54.7%)	933 (49.5%)	951 (50.5%)	15.5% (-4.6%, 31.9%)	20.8% (-0.4%, 37.5%)
14-30 days	0 (0.0%)	222 (100.0%)	10 (1.0%)	951 (99.0%)	N/A	N/A
31-90 days	9 (3.9%)	222 (96.1%)	100 (9.5%)	951 (90.5%)	61.4% (22.6%, 80.8%)	51.0% (-0.1%, 76.0%)
91-150 days	58 (20.7%)	222 (79.3%)	402 (29.7%)	951 (70.3%)	38.2% (15.6%, 54.7%)	38.6% (14.8%, 55.7%)
>150 days	117 (34.5%)	222 (65.5%)	421 (30.7%)	951 (69.3%)	-16.0% (-34.7%, 7.4%)	-4.5% (-27.9%, 20.9%)
BA.4	38 (46.3%)	44 (53.7%)	149 (50.0%)	149 (50.0%)	13.6% (-29.1%, 47.1%)	17.6% (-30.1%, 52.6%)
14-30 days	0 (0.0%)	44 (100.0%)	0 (0.0%)	149 (100.0%)	N/A	N/A
31-90 days	0 (0.0%)	44 (100.0%)	7 (4.5%)	149 (95.5%)	N/A	N/A
91-150 days	5 (10.2%)	44 (89.8%)	44 (22.8%)	149 (77.2%)	61.5% (-2.9%, 85.6%)	55.4% (-20.2%, 84.1%)
>150 days	33 (42.9%)	44 (57.1%)	98 (39.7%)	149 (60.3%)	-12.3% (-47.8%, 32.1%)	-2.7% (-45.8%, 42.8%)
BA.5	97 (40.6%)	142 (59.4%)	457 (51.6%)	429 (48.4%)	35.9% (14.3%, 52.0%)	37.9% (14.8%, 54.7%)
14-30 days	0 (0.0%)	142 (100.0%)	9 (2.1%)	429 (97.9%)	N/A	N/A
31-90 days	2 (1.4%)	142 (98.6%)	39 (8.3%)	429 (91.7%)	84.5% (35.0%, 96.3%)	84.1% (32.8%, 96.2%)
91-150 days	21 (12.9%)	142 (87.1%)	128 (23.0%)	429 (77.0%)	50.4% (18.4%, 69.9%)	48.6% (14.1%, 69.3%)
>150 days	74 (34.3%)	142 (65.7%)	281 (39.6%)	429 (60.4%)	20.4% (-8.6%, 42.2%)	23.4% (-7.9%, 46.0%)

[a] When the OR or its 95% CI was >1, the VE or its 95% CI was transformed as ([1/OR] – 1) x 100

[b] Adjusted for age, sex, race/ethnicity, month of specimen collection, history of SARS-CoV-2 molecular test, number of outpatient and virtual visits, and time between prior SARS-CoV-2 infection and specimen collection date. Medical center area removed from adjustment set due to lack of model convergence.
[c] BA.2 excluding BA.2.12.1.
CI, confidence interval; OR, odds ratio; VE, vaccine effectiveness.

Supplementary Table 15b. Relative vaccine effectiveness of 3 vs. 2 doses of mRNA-1273 against infection with SARS-CoV-2 variants by time since vaccination among those with known history of SARS-CoV-2 infection

	Test Positive		Test Negative		rVE (95% CI)[a]	
Subvariant/Time since 3rd dose	3-dose Vaccinated (%)	2-dose Vaccinated (%)	3-dose Vaccinated (%)	2-dose Vaccinated (%)	Unadjusted	Adjusted[b]
BA.1	86 (34.0%)	167 (66.0%)	1002 (51.7%)	937 (48.3%)	51.8% (36.6%, 63.4%)	66.0% (53.7%, 75.1%)
14-30 days	9 (5.1%)	167 (94.9%)	216 (18.7%)	937 (81.3%)	76.6% (53.5%, 88.2%)	80.8% (61.4%, 90.4%)
31-90 days	57 (25.4%)	167 (74.6%)	670 (41.7%)	937 (58.3%)	52.3% (34.5%, 65.2%)	64.6% (49.9%, 75.0%)
91-150 days	18 (9.7%)	167 (90.3%)	106 (10.2%)	937 (89.8%)	4.7% (-38.0%, 43.7%)	42.1% (-5.3%, 68.3%)
>150 days	2 (1.2%)	167 (98.8%)	10 (1.1%)	937 (98.9%)	-10.9% (-80.6%, 75.6%)	39.9% (-66.2%, 87.8%)
BA.2[c]	211 (64.7%)	115 (35.3%)	1199 (65.7%)	626 (34.3%)	4.2% (-18.4%, 25.2%)	8.6% (-17.5%, 31.1%)
14-30 days	1 (0.9%)	115 (99.1%)	25 (3.8%)	626 (96.2%)	78.2% (-38.4%, 97.1%)	80.2% (-35.1%, 97.5%)
31-90 days	21 (15.4%)	115 (84.6%)	194 (23.7%)	626 (76.3%)	41.1% (3.6%, 64.0%)	24.5% (-21.3%, 55.1%)
91-150 days	84 (42.2%)	115 (57.8%)	590 (48.5%)	626 (51.5%)	22.5% (-4.7%, 42.8%)	21.0% (-9.3%, 43.3%)
>150 days	105 (47.7%)	115 (52.3%)	390 (38.4%)	626 (61.6%)	-31.8% (-49.1%, -8.5%)	-23.7% (-46.4%, 7.9%)
BA.2.12.1	184 (71.0%)	75 (29.0%)	933 (65.5%)	491 (34.5%)	-22.5% (-42.0%, 3.4%)	-14.6% (-38.4%, 15.5%)
14-30 days	0 (0.0%)	75 (100.0%)	10 (2.0%)	491 (98.0%)	N/A	N/A
31-90 days	9 (10.7%)	75 (89.3%)	100 (16.9%)	491 (83.1%)	41.1% (-17.7%, 71.4%)	21.1% (-40.3%, 62.8%)
91-150 days	58 (43.6%)	75 (56.4%)	402 (45.0%)	491 (55.0%)	5.5% (-26.7%, 34.6%)	7.6% (-27.4%, 38.1%)
>150 days	117 (60.9%)	75 (39.1%)	421 (46.2%)	491 (53.8%)	-45.0% (-60.0%, -24.5%)	-36.5% (-56.3%, -7.9%)
BA.4[d]	38 (59.4%)	26 (40.6%)	149 (60.8%)	96 (39.2%)	5.8% (-39.4%, 46.3%)	18.7% (-36.5%, 58.0%)
14-30 days	0 (0.0%)	26 (100.0%)	0 (0.0%)	96 (100.0%)	N/A	N/A
31-90 days	0 (0.0%)	26 (100.0%)	7 (6.8%)	96 (93.2%)	N/A	N/A
91-150 days	5 (16.1%)	26 (83.9%)	44 (31.4%)	96 (68.6%)	58.0% (-14.2%, 84.9%)	64.4% (-8.7%, 88.4%)
>150 days	33 (55.9%)	26 (44.1%)	98 (50.5%)	96 (49.5%)	-19.6% (-55.2%, 30.8%)	-4.6% (-52.0%, 47.3%)
BA.5	97 (68.3%)	45 (31.7%)	457 (66.5%)	230 (33.5%)	-7.8% (-37.4%, 26.4%)	-0.4% (-37.1%, 36.6%)
14-30 days	0 (0.0%)	45 (100.0%)	9 (3.8%)	230 (96.2%)	N/A	N/A
31-90 days	2 (4.3%)	45 (95.7%)	39 (14.5%)	230 (85.5%)	73.8% (-11.1%, 93.9%)	74.9% (-10.0%, 94.3%)
91-150 days	21 (31.8%)	45 (68.2%)	128 (35.8%)	230 (64.2%)	16.1% (-32.0%, 52.2%)	12.5% (-38.1%, 52.6%)
>150 days	74 (62.2%)	45 (37.8%)	281 (55.0%)	230 (45.0%)	-25.7% (-50.7%, 10.6%)	-22.2% (-52.9%, 22.1%)

[a] When the OR or its 95% CI was >1, the rVE or its 95% CI was transformed as ([1/OR] – 1) x 100

[b] Adjusted for age, sex, race/ethnicity, month of specimen collection, history of SARS-CoV-2 molecular test, number of outpatient and virtual visits, time between second dose and specimen collection date, and time between prior SARS-CoV-2 infection and specimen collection date. Medical center area removed from adjustment set due to lack of model convergence.
[c] BA.2 excluding BA.2.12.1.
[d] History of SARS-CoV-2 molecular test removed from adjustment set due to lack of model convergence.
CI, confidence interval; OR, odds ratio; rVE, relative vaccine effectiveness.

Study Protocol

Real-World Study of the Effectiveness of Moderna COVID-19 Vaccine

mRNA-1273-P901 RWS

FINAL

August 19, 2022

Kaiser Permanente Southern California
Hung Fu Tseng, PhD, MPH
Research Scientist III

Katia Bruxvoort, PhD, MPH
Adjunct Investigator

Ana Florea, PhD, MPH
Post-Doctoral Research Fellow

Jennifer Ku, PhD, MPH
Post-Doctoral Research Fellow

Moderna, Inc.
Scott Chavers, PhD, MPH
Lead Epidemiologist

Approvals

Approvers, Organization & Role	Approval Status & Signature	Date
Hung-Fu Tseng<br>KPSC Research and Evaluation<br>Principal Investigator		
Jacqueline Miller<br>Therapeutic Area Head, Infectious Disease Development<br>Moderna Inc.		

Version History

Version	Date	Primary Author/Reviewer	Description
0.1	January 22, 2021	Katia Bruxvoort	1st Draft Submitted to Moderna for review
0.2	February 9, 2021	Katia Bruxvoort	2nd Draft Submitted to Moderna for review
1.0	February 17, 2021	Katia Bruxvoort	Final Draft Submitted to Moderna for review
1.1	May 21, 2021	Katia Bruxvoort	Draft Submitted to Moderna for review – Update: Addition of adolescents (12-17 years); addition of timing of select secondary analyses; clarifications in response to questions from EMA and CBER.
2.0	June 4, 2021	Katia Bruxvoort	Final Draft Submitted to Moderna for review
2.1	August 4, 2021	Katia Bruxvoort	Draft Submitted to Moderna for review – Update: Addition of vaccine effectiveness against SARS-CoV-2 variants.
3.0	September 7, 2021	Katia Bruxvoort	Final Draft Submitted to Moderna for review
3.1	December 10, 2021	Ana Florea<br>Jennifer Ku	Draft Submitted to Moderna for review – Update: Addition of vaccine effectiveness of 3 doses in immunocompromised and booster dose in non-immunocompromised individuals.
4.0	December 17, 2021	Ana Florea<br>Jennifer Ku	Final Draft Submitted to Moderna for review
4.1	July 22, 2022	Ana Florea<br>Jennifer Ku	Draft Submitted to Moderna for review – Update: modification of 4th Interim Analysis plan, secondary objectives 18 and 19 to estimate the relative vaccine effectiveness of

			3 doses vs. 2 doses, relative vaccine effectiveness for forthcoming analyses in adults (≥18 years); addition of children (6 months – 11 years), additional doses, rapid antigen test data, new vaccine formulation(s).
5.0	August 19, 2022	Ana Florea<br>Jennifer Ku	Final Draft Submitted to Moderna for review

Table of Contents

List of Tables ... 36
List of Figures ... 37
List of Abbreviations ... 38
1 Synopsis ... 39
2 Background ... 41
3 Research Objectives ... 42
3.1 Primary Objectives ... 42
3.2 Secondary Objectives ... 42
4 Study Setting ... 44
4.1 COVID-19 Vaccination at KPSC ... 45
4.2 COVID-19 at KPSC ... 46
4.3 SARS-CoV-2 Whole Genome Sequencing ... 48
5 Study Design Overview ... 49
6 Study Population ... 50
6.1 Eligibility Criteria ... 50
6.2 Index Dates ... 50
7 Measures ... 52
7.1 Exposure Definition ... 52
7.2 Outcome Definitions ... 52
7.3 Other Variable Definitions ... 53
8 Statistical Analysis for Study Objectives 1-15 ... 56
8.1 Analysis Plan ... 56
8.2 Descriptive Analysis ... 58
8.3 Primary Analyses ... 58
8.4 Secondary Analyses (Secondary Objectives 1-15) ... 58
9 Sample Size and Power Estimation ... 61
10 Statistical Analysis Plan for Vaccine Effectiveness Against SARS-CoV-2 Variants (Secondary Objectives 16-17) ... 63
10.1 Test-negative design (TND) ... 63
10.2 Other considerations ... 65
11 Statistical Analysis Plan for Vaccine Effectiveness of Booster and Third Dose (Secondary Objectives 18-19) ... 66
11.1 Booster Vaccination in Non-Immunocompromised Individuals ... 66

11.2 Third Primary Dose in Immunocompromised Individuals 67
12 Timelines 68
13 Data Management 69
14 Limitations 70
15 Human Subjects Protection 71
16 References 72
17 Appendix 1 - Protocol Amendment 4 (version 5.0) 74
17.1 Updates to COVID-19 Vaccination 74
17.1.1 Primary Series 74
17.1.2 Booster Dose 74
17.2 Modification to 4th Interim Analysis 74
17.3 Study Design Modifications 75
17.3.1 SARS-CoV-2 Infection Definition 75
17.3.2 Relative Vaccine Effectiveness 75
17.3.3 Vaccine Effectiveness Among Children Ages 6 months Through 17 Years 75
17.3.4 Vaccine Effectiveness Against SARS-CoV-2 Variants 76
17.3.5 Booster doses in Immunocompromised and Non-Immunocompromised Individuals 76
17.3.6 New or Updated mRNA-1273 Formulations 76

List of Tables

Table 1. Demographic characteristics of Kaiser Permanente Southern California members on January 1, 2021 45
Table 2. SARS-CoV-2 molecular tests and COVID-19 diagnoses among members of Kaiser Permanente Southern California during 01/01/2020-12/31/2020 46
Table 3. List of possible covariates 53
Table 4. Power calculations for 1st interim analysis of Primary Objective 1 for various sample sizes and vaccine effectiveness estimates 61
Table 5. Power calculations for 1st interim analysis of Primary Objective 2 for various sample sizes and vaccine effectiveness estimates 62
Table 6. Estimated Project Timelines 68

List of Figures

Figure 1. Analysis Plan for Study Objectives 1-15..........57

List of Abbreviations

ACIP	Advisory Committee on Immunization Practices
AIDS	Acquired Immunodeficiency Syndrome
ASD	Absolute Standardized Difference
BMI	Body Mass Index
CAIR	California Immunization Registry
CDC	Centers for Disease Control and Prevention
CI	Confidence Interval
COPD	Chronic Obstructive Pulmonary Disease
COVID-19	Coronavirus Disease 2019
Ct	Cycle Threshold
EHR	Electronic Health Record
EUA	Emergency Use Authorization
HIPAA	Health Insurance Portability and Accountability Act of 1996 (U.S.)
HR	Hazard Ratio
HIV	Human Immunodeficiency Virus
ICD-10	International Classification of Diseases, 10th Revision
IRB	Institutional Review Board
IPTW	Inverse Probability of Treatment Weighting
KPSC	Kaiser Permanente of Southern California
NLP	Natural Language Processing
MCE	Multi-County Entity
mRNA	Messenger RNA
PHI	Protected Health Information
rVE	Relative Vaccine Effectiveness
SARS-CoV-2	Severe Acute Respiratory Syndrome Coronavirus 2
SGTF	S-Gene Target Failure
TND	Test-Negative Design
U.S.	United States
VE	Vaccine Effectiveness
VOC	Variant of Concern
VOI	Variant of Interest
WGS	Whole Genome Sequencing

1 Synopsis

Severe acute respiratory syndrome coronavirus 2 (SARS-CoV-2), the virus causing the coronavirus disease 2019 (COVID-19) pandemic, has caused high global morbidity and mortality,[1] with multiple SARS-CoV-2 variants emerging over the course of the pandemic. [2-4] COVID-19 vaccines have been developed at unprecedented speed, including the Moderna mRNA-1273 SARS-CoV-2 vaccine (Moderna COVID-19 vaccine, hereafter), which received Emergency Use Authorization (EUA) in the United States (U.S.) on December 18, 2020.[5] Moderna COVID-19 vaccine is a lipid nanoparticle encapsulated messenger RNA (mRNA)-based vaccine encoding the prefusion-stabilized spike glycoprotein of SARS-CoV-2. In a phase 3 randomized, observer-blinded, placebo-controlled trial enrolling approximately 30,000 participants, vaccine efficacy of Moderna COVID-19 vaccine was 94.1% and was generally consistent across age, sex, race/ethnicity, and risk status. No safety concerns were identified.[6] However, post-authorization studies are critically needed to determine Moderna COVID-19 vaccine effectiveness and durability in real-world settings among diverse populations.

In this protocol, we describe an observational cohort study at Kaiser Permanente Southern California (KPSC), a large, integrated health care system. The primary objectives are to evaluate the vaccine effectiveness (VE) of 2 doses of Moderna COVID-19 vaccine in preventing SARS-CoV-2 infection and severe COVID-19 disease. SARS-CoV-2 infection will be defined as a positive molecular diagnostic test among symptomatic or asymptomatic individuals or a COVID-19 diagnosis code. Severe COVID-19 disease will be defined as COVID-19 hospitalization or mortality. For the primary objectives, KPSC members meeting inclusion criteria will be considered exposed if they received 2 doses of Moderna COVID-19 vaccine during 2021, and the unexposed comparison group will be a similar unvaccinated population. Individuals will be followed through electronic health records for COVID-19 outcomes.

For first and second interim analyses, unvaccinated individuals will be randomly selected and n:1 matched to vaccinated individuals by age, sex, and race/ethnicity (if sample size allows), and will be assigned an index date based on the vaccination date of their matched vaccinated individual. For subsequent interim analyses and the final analysis, the number of eligible unvaccinated individuals in the KPSC population may be insufficient for individual matching; if so, frequency matching will be employed. In all analyses, efforts will be dedicated to identifying and adjusting for potential confounders. Cox proportional hazards regression will be used to estimate unadjusted and adjusted hazards ratios (HRs). VE (%) will be estimated as (1 – adjusted HR) × 100. Propensity score analyses with inverse probability of treatment weighting (IPTW) will be considered for final analyses.

Secondary analyses will be conducted to examine VE of 2 doses of Moderna COVID-19 vaccine in preventing SARS-CoV-2 infection stratified by age, sex, and race/ethnicity, as well as among individuals with chronic conditions, immunocompromised individuals, individuals with autoimmune conditions, frail individuals, pregnant women, and individuals with a history of SARS-CoV-2 infection. In addition, we will examine the VE of 2 doses of Moderna COVID-19 vaccine in preventing SARS-CoV-2 infection when given concomitantly with another vaccine. We will examine the VE of 2 doses of Moderna COVID-19 vaccine in preventing asymptomatic SARS-CoV-2 infection and the VE of 2 doses of Moderna COVID-19 vaccine in preventing symptomatic SARS-CoV-2 infection. Also, we will examine durability of 2 doses of Moderna COVID-19 vaccine in preventing SARS-CoV-2 infection and severe COVID-19, as well as the VE of 1 dose of Moderna COVID-19 vaccine in preventing SARS-CoV-2 infection and severe COVID-19. Using a test-negative design, we will examine the VE of 2 doses of Moderna COVID-19 vaccine against SARS-CoV-2 variants, and the VE of 1 dose of Moderna COVID-19 vaccine against SARS-CoV-2 variants. Additionally, using a matched cohort design, we will

examine the VE of a booster dose of Moderna vaccine against SARS-CoV-2 infection and severe COVID-19 in non-immunocompromised individuals. We will also use a matched cohort design to examine the VE of 3 doses (primary series) of Moderna COVID-19 vaccine against SARS-CoV-2 infection and severe COVID-19 in immunocompromised individuals. Some secondary analyses may be underpowered; their meaningfulness will depend on uptake of Moderna COVID-19 vaccine in these patient populations.

Analyses will be conducted to estimate relative vaccine effectiveness (rVE), effectiveness of additional booster doses, effectiveness in children 6 months to 17 years of age, and effectiveness of new vaccine formulations (Section 17 Appendix 1).

2 Background

Severe acute respiratory syndrome coronavirus 2 (SARS-CoV-2), the virus causing the coronavirus disease 2019 (COVID-19) pandemic, has led to more than 93 million cases and 2 million deaths globally as of January 17, 2021. [1] In response to the rapidly increasing burden, COVID-19 vaccine development has occurred at unprecedented speed, with several vaccines receiving authorizations or approvals worldwide in the first year after SARS-CoV-2 emergence. The Moderna mRNA-1273 SARS-CoV-2 vaccine (Moderna COVID-19 vaccine, hereafter) is a lipid nanoparticle encapsulated messenger RNA (mRNA)-based vaccine encoding the prefusion-stabilized spike glycoprotein of SARS-CoV-2. The spike glycoprotein recognizes and binds to angiotensin-converting enzyme 2 receptors on host cells, facilitating viral entry. [7] The Moderna COVID-19 vaccine mimics natural infection by carrying genetic instructions to host cells to make the spike glycoprotein antigen, inducing T-cell and antibody responses. In early clinical trials, the Moderna COVID-19 vaccine demonstrated anti-SARS-CoV-2 immunogenicity and had an acceptable safety profile, with no trial-limiting severe adverse events. [8, 9]

A phase 3 randomized, observer-blinded, placebo-controlled trial of Moderna COVID-19 vaccine enrolled participants aged ≥18 years from July to October 2020 at 99 sites in the United States (U.S.). The trial enrolled approximately 30,000 participants who were randomized 1:1 to receive two intramuscular injections of Moderna COVID-19 vaccine or placebo 28 days apart. The incidence of symptomatic COVID-19 illness was 56.5 per 1000 person-years in the placebo group and 3.3 per 1000 person-years in the Moderna COVID-19 vaccine group. [6] Vaccine efficacy was 94.1% and was generally consistent across age, sex, race/ethnicity, and risk status.

Multiple SARS-CoV-2 variants of concern (VOCs) and variants of interest (VOIs) have also been identified over the course of the pandemic, some of which have been associated with greater disease severity and/or transmissibility. [2-4]

Following release of the phase 3 results in adults, the Moderna COVID-19 vaccine received Emergency Use Authorization (EUA) as a 2-dose primary series (100 µg dose) in the U.S. on December 18, 2020. On January 31, 2022, the FDA approved the Moderna COVID-19 vaccine after review of the Biologics License Application; the approved vaccine is marketed as Spikevax. [10, 11] The FDA authorized the use of the Moderna COVID-19 vaccine in individuals 6 months through 17 years of age on June 17, 2022. [12] COVID-19 vaccination was implemented in a phased approach, per Advisory Committee on Immunization Practices (ACIP) recommendations and state guidelines. In this protocol, we detail an observational cohort study to evaluate real-world vaccine effectiveness and durability of Moderna COVID-19 vaccine among a diverse population at Kaiser Permanente Southern California (KPSC).

3 Research Objectives

We propose to evaluate the vaccine effectiveness (VE) of Moderna COVID-19 vaccine in preventing SARS-CoV-2 infection (symptomatic and asymptomatic) and severe COVID-19 disease (hospitalizations and mortality) at KPSC with the primary objectives below. Secondary objectives will be assessed but may be underpowered.

3.1 Primary Objectives

1. To evaluate the effectiveness of 2 doses of Moderna COVID-19 vaccine in preventing SARS-CoV-2 infection
2. To evaluate the effectiveness of 2 doses of Moderna COVID-19 vaccine in preventing severe COVID-19 disease

3.2 Secondary Objectives

1. To evaluate the effectiveness of 2 doses of Moderna COVID-19 vaccine in preventing SARS-CoV-2 infection by age and by sex
2. To evaluate the effectiveness of 2 doses of Moderna COVID-19 vaccine in preventing SARS-CoV-2 infection by race/ethnicity groups
3. To evaluate the effectiveness of 2 doses of Moderna COVID-19 vaccine in preventing SARS-CoV-2 infection in individuals with chronic diseases (e.g., chronic kidney disease, lung disease including chronic obstructive pulmonary disease [COPD] and asthma, diabetes)
4. To evaluate the effectiveness of 2 doses of Moderna COVID-19 vaccine in preventing SARS-CoV-2 infection in individuals who are immunocompromised (e.g., HIV, cancer, transplant, immunosuppressive medications)
5. To evaluate the effectiveness of 2 doses of Moderna COVID-19 vaccine in preventing SARS-CoV-2 infection in individuals with autoimmune conditions (e.g., rheumatoid arthritis, inflammatory bowel disease, psoriasis, psoriatic arthritis, multiple sclerosis, systemic lupus erythematosus)
6. To evaluate the effectiveness of 2 doses of Moderna COVID-19 vaccine in preventing SARS-CoV-2 infection in frail individuals
7. To evaluate the effectiveness of 2 doses of Moderna COVID-19 vaccine administered during pregnancy in preventing SARS-CoV-2 infection
8. To evaluate the effectiveness of 2 doses of Moderna COVID-19 vaccine in preventing SARS-CoV-2 infection among individuals with a history of SARS-CoV-2 infection
9. To evaluate the effectiveness of 2 doses of Moderna COVID-19 vaccine in preventing SARS-CoV-2 infection when given concomitantly with another vaccine
10. To evaluate the effectiveness of 2 doses of Moderna COVID-19 vaccine in preventing asymptomatic SARS-CoV-2 infection
11. To evaluate the effectiveness of 2 doses of Moderna COVID-19 vaccine in preventing symptomatic SARS-CoV-2 infection

12. To evaluate the durability of 2 doses of Moderna COVID-19 vaccine in preventing SARS-CoV-2 infection
13. To evaluate the durability of 2 doses of Moderna COVID-19 vaccine in preventing severe COVID-19 disease
14. To evaluate the effectiveness of 1 dose of Moderna COVID-19 vaccine in preventing SARS-CoV-2 infection
15. To evaluate the effectiveness of 1 dose of Moderna COVID-19 vaccine in preventing severe COVID-19 disease
16. To assess the effectiveness of two doses of Moderna COVID-19 vaccine against SARS-CoV-2 variants (test-negative design)
17. To assess the effectiveness of one dose of Moderna COVID-19 vaccine against SARS-CoV-2 variants (test-negative design)
18. To assess the effectiveness of a booster dose of Moderna COVID-19 vaccine in preventing SARS-CoV-2 infection and severe COVID-19 disease in non-immunocompromised individuals
19. To assess the effectiveness of 3 doses (primary series) of Moderna COVID-19 vaccine in preventing SARS-CoV-2 infection and severe COVID-19 disease in immunocompromised individuals

4 Study Setting

KPSC is one of the largest not-for-profit health plans and integrated health care systems in the U.S., providing an ideal environment for population-based research. KPSC's population includes more than 4.5 million members, of which 702,136 adults are aged ≥65 years and 341,464 are between 12 and 17 years old (Table 1). The diverse demographic makeup, including 260 different ethnicities and more than 150 different languages, closely mirrors the Southern California population. Compared to the racial/ethnic distribution of the U.S. population, KPSC membership is composed of twice as many individuals of Asian/Pacific Island descent and three times as many Hispanic individuals. [13]

KPSC facilities include hospitals and medical offices, all linked by an information infrastructure that supports both clinical practice and business needs. Health information from this infrastructure can be leveraged for research purposes. More than 90 percent of members remain in the health plan after one year; more than three-quarters remain after three years. The large, diverse, and stable population permits the rapid accrual of a representative sample size and offers the ability to evaluate long-term implications of immunization.
Kaiser Permanente HealthConnect® is the largest and most advanced civilian electronic health record system available in the U.S. In addition to supporting patient care, this robust system facilitates research, providing access to electronic health records (EHR) for the research team. The medical record number serves as a unique identifier linking all medical encounters for each member. Care received in the outpatient, inpatient, and emergency settings is documented in the EHR and captured in research databases. Care received outside the KPSC system is captured through claims. Details of care are available at the fingertips of researchers in near real time.
Our EHR include a variety of data that can be used for research:
Membership: Includes demographic information such as sex, date of birth, and race/ethnicity.
Diagnosis: Includes International Classification of Diseases, 10th revision (ICD-10) codes.
Procedure: Includes ICD-10, Current Procedural Terminology (CPT), and Systematized Nomenclature of Medicine (SNOMED) codes.
Immunization: Includes vaccine name, date of vaccination, route of administration, facility where vaccine was administered, dose, manufacturer, and lot number.
Laboratory: Includes laboratory orders and results.
Pharmacy: Includes National Drug Codes (NDC) and Generic Product Identifier (GPI) codes. More than 95 percent of members have a drug benefit with minimal copayments.
Mortality: Includes deaths from hospital and membership databases, as well as from state and national death files, when available.
Birth: Includes pregnancy related information such as gestational age, birth weight, and Apgar scores.
Clinical notes: Allows for natural language processing of clinical notes to identify outcomes not easily identified through structured data.

Table 1. Demographic characteristics of Kaiser Permanente Southern California members on January 1, 2021

	Number of members
Total population	4,573,594
**Sex**	
Male	2,360,957
Female	2,212,637
**Age (years)**	
<6	278,559
6 to <12	308,704
12 to <18	341,464
18 to <65	2,942,731
65 to <75	432,045
≥75	270,091
**Race**	
White	2,470,912
Black or African American	359,270
American Indian & Alaska Native	21,137
Asian	487,947
Native Hawaiian and Other Pacific Islander	37,107
Other race	1,172,221
Two or more races	25,000
**Ethnicity**	
Hispanic or Latinx (of any race)	1,829,983

4.1 COVID-19 Vaccination at KPSC

Immunizations are an important part of KPSC's overall focus on preventive care. The organization is one of the top-rated health maintenance organizations for meeting national standards of care, which include measures of childhood and adult immunization. Recommended vaccines are provided at no cost to KPSC members. KPSC thus provides an excellent real-world setting in which to understand the effectiveness of vaccines used in the course of routine clinical care.

According to California's COVID-19 Vaccination Plan from October 2020, the state would allocate vaccines directly to large multi-jurisdictional entities, such as health providers and systems with locations in multiple counties. A multi-county entity (MCE) is a health system that has facilities in more than two California counties to centrally support local implementation in all of its locations, set policy for all of its facilities, order and store vaccine, has a centralized pharmacy, and has a demonstrated track record in immunizing their staff. KPSC is one of the largest California MCEs.

In December 2020, KPSC began to administer Moderna COVID-19 vaccine to eligible individuals aged ≥18 years and Pfizer-BioNTech COVID-19 vaccine to eligible individuals aged ≥16 years. In March 2021, KPSC began to administer Janssen COVID-19 vaccine to eligible

individuals aged ≥18 years. KPSC is able to differentiate COVID-19 vaccine products in the EHR.
Moderna COVID-19 vaccine was authorized and recommended for individuals 6 months to 17 years of age in June 2022. As KPSC administers Moderna COVID-19 vaccine to this population, they will be included in this study.
To ensure equitable distribution, California was allocating COVID-19 vaccines as they became available. KPSC followed the state's COVID-19 vaccine prioritization. Individuals were prioritized for vaccination as follows:

- Phase 1A (started in December 2020)
  - Healthcare workers
  - Long-term care residents
- Phase 1B (started in January 2021)
  - Individuals aged ≥65 years
  - Sector populations:
    - Education and childcare
    - Emergency services
    - Food and agriculture
- Individuals aged 16-64 years (started in March 2021)
  - At the very highest risk for morbidity and mortality from COVID-19 as a direct result of one or more of the severe health conditions
- Individuals aged 16 years and older (started in April 2021)
- Individuals aged 12 years and older (started in May 2021)
- Individuals aged 5 years and older (started in November 2021)
- Individuals aged 6 months and older (started in June 2022)

KPSC is an approved COVID-19 vaccine provider and is receiving Moderna COVID-19 vaccine from the state of California. KPSC also received Moderna COVID-19 vaccine directly from Moderna as part of this real-world effectiveness study.

4.2 COVID-19 at KPSC

At KPSC, diagnostic testing for SARS-CoV-2 is offered free of charge with an order from a KPSC physician, which can be requested by email, in person or through a virtual visit. Prioritization for testing has evolved during the pandemic, with an initial emphasis on individuals with symptoms (particularly high-risk groups) and prior to hospital admissions or certain outpatient procedures, with gradual expansion in 2020 to members without COVID-19 associated symptoms.
Testing in 2020 was primarily conducted by RT-PCR of nasopharyngeal/oropharyngeal swabs using the Roche cobas® SARS-CoV-2 assay on the Roche cobas® 6800 and 8800 analyzers or nasal/oropharyngeal swabs using the Aptima® SARS-CoV-2 assay on the Hologic Panther® analyzers. A small number of Abbott ID NOW™ COVID-19 rapid tests were conducted in limited settings (e.g., obstetrics, pulmonary medicine, and infectious disease departments); these were phased out by end of 2020. In November 2020, KPSC opened a new regional COVID-19 laboratory with Thermo Fisher Scientific Amplitude Solution instruments and also added saliva testing for asymptomatic individuals, increasing testing capacity to approximately 52,000 tests per day.

Table 2. SARS-CoV-2 molecular tests and COVID-19 diagnoses among members of Kaiser Permanente Southern California during 01/01/2020-12/31/2020

	Age (years)	Total

	12-17	18-64	≥65	
Number of patients tested for SARS-CoV-2 with RT-PCR test	48,290	825,670	184,197	1,058,157
Number of patients with a positive SARS-CoV-2 RT-PCR test	13,788	200,403	24,452	238,643
Number of patients with a SARS-CoV-2 infection[1]	15,807	247,628	31,960	295,395
Number of patients admitted to hospital with a SARS-CoV-2 infection[1]	87	10,871	7,980	18,938
Number of patients admitted to ICU with a SARS-CoV-2 infection[1]	13	2,928	2,227	5,168
Number of deaths within 31 days after the first SARS-CoV-2 infection[1]	1	715	2,038	2,754

[1]A SARS-CoV-2 infection includes those with a positive RT-PCR test result or a COVID-19 diagnosis code only.

As of December 31, 2020, over 1 million SARS-CoV-2 RT-PCR tests had been performed at KPSC, of which 17.4% were among individuals aged ≥65 years and 4.6% were among adolescents aged 12-17 years (Table 2). There were 238,643 members testing positive (13,788 members aged 12-17 years and 24,452 members aged ≥65 years). Additional members were diagnosed with COVID-19 based on positive test results outside of KPSC or clinical presentation and contact history, for a total of 56,752 members with a COVID-19 clinical diagnosis only. Approximately 25.0% (7,980/31,960) of COVID-19 patients aged ≥65 years were hospitalized, compared to only 4.4% (10,871/247,628) who were hospitalized among patients aged 18-64 years and 0.6% (87/15,807) who were hospitalized among patients aged 12-17 years. The proportion of COVID-19 patients being admitted to the ICU was 7.0% in patients aged ≥65 years versus 1.2% in patients aged 18-64 years. The proportion of COVID-19 patients who died within 31 days after a COVID-19 diagnosis or a positive test was 6.4% in patients aged ≥65 years versus 0.29% in patients aged 18-64 years.

In January 2021, voluntary weekly saliva testing was introduced for physicians and employees working in patient-facing areas.

As of July 2021, the TaqPath™ COVID-19 High-Throughput Combo Kit on the Thermo Fisher Scientific Amplitude Solution is used for the majority of SARS-CoV-2 molecular tests. A small proportion of tests are conducted using the Roche cobas® SARS-CoV-2 assay on the Roche cobas® 8800 System or the Roche cobas® SARS-CoV-2 & Influenza A/B assay on the Roche cobas® Liat® System; these tests can be ordered in limited circumstances when a provider requires an expedited result. The Aptima® SARS-CoV-2 assay on the Hologic Panther® System was phased out in early 2021.

As of July 2021, asymptomatic individuals who receive a COVID-19 test at KPSC may include individuals without COVID-19 symptoms who require or request testing. Both vaccinated and unvaccinated individuals are required to be tested for SARS-CoV-2 prior to KPSC procedures or admission. In addition, asymptomatic individuals can request testing (regardless of vaccination status) for any of the following reasons: travel, exposure to or close contact with a COVID-19-positive individual, residents or employees of congregate living facilities, employment (e.g., healthcare workers, first responders, essential workers, or any others who require testing for workplace), school or daycare, or any other reason. In addition, asymptomatic individuals who are physicians or other employees of KPSC can receive voluntary weekly saliva testing. Testing for asymptomatic individuals most commonly uses saliva samples rather than nasal/oropharyngeal swabs.

SARS-CoV-2 rapid antigen test results were collected at KPSC starting in approximately early 2022. These include member self-reported results as well as results from rapid antigen tests performed at KPSC.

4.3 SARS-CoV-2 Whole Genome Sequencing

KPSC began saving positive molecular SARS-CoV-2 test specimens in March 2021 to conduct whole genome sequencing (WGS). As part of this effort, positive SARS-CoV-2 molecular test specimens will be sent to an external laboratory for sequencing and variant identification. For specimens where SARS-CoV-2 lineage could not be identified by WGS, S-gene target failure (SGTF) data may be used when available along with dominant variant trends at the time of specimen collection.

5 Study Design Overview

For Primary Objectives 1 and 2, we will conduct an observational cohort study to evaluate the VE of 2 doses of Moderna COVID-19 vaccine in preventing SARS-CoV-2 infection and severe COVID-19. Vaccine exposure, COVID-19 outcomes, and covariates will be identified from EHR, as described in Section 7 below. All individuals who receive Moderna COVID-19 vaccine from December 2020 to December 2021 and meet eligibility criteria specified in Section 6.1 below will be included in the study ("vaccinated", hereafter). The unvaccinated comparator cohort will comprise individuals who have not received Moderna COVID-19 vaccine or any other COVID-19 vaccine as of the index date of their matched vaccinated individual (see Section 6.2 for details on index date) and who meet eligibility criteria specified in Section 6.1 below. Unvaccinated individuals who subsequently receive Moderna COVID-19 vaccine may contribute person-time to both unvaccinated and vaccinated cohorts. Individuals will be followed up through EHR for occurrence of COVID-19 outcomes until the end of the study period (December 31, 2023 for final analysis), or censoring events (termination of KPSC membership allowing for a 31-day gap, death, receipt of a COVID-19 vaccine).
Six interim analyses and a final analysis will be conducted. For the first and second interim analyses, unvaccinated individuals will be randomly selected and n:1 matched to vaccinated individuals by age, sex, and race/ethnicity (if sample size allows) and will be assigned an index date based on the vaccination date of their matched vaccinated individual. For subsequent interim analyses and the final analysis, the number of eligible unvaccinated individuals in the KPSC population may be insufficient for individual matching; if so, frequency matching will be employed. In all analyses, efforts will be dedicated to identifying and adjusting for potential confounders. Cox proportional hazards regression will be used to estimate unadjusted and adjusted HRs. VE (%) will be estimated as (1 – adjusted HR) × 100. Propensity score analyses with inverse probability of treatment weighting (IPTW) will be considered for final analyses. Analyses will also be conducted for Secondary Objectives 1-15 to estimate VE of 2 doses of Moderna COVID-19 vaccine in sub-populations of interest, to estimate VE of 2 doses of Moderna COVID-19 vaccine when received concomitantly with other vaccines, to estimate VE of 2 doses of Moderna COVID-19 vaccine in preventing asymptomatic SARS-CoV-2 infection and symptomatic SARS-CoV-2 infection, to estimate durability of 2 doses of Moderna COVID-19 vaccine, and to estimate VE of 1 dose of Moderna COVID-19 vaccine.

We will also conduct analyses to assess VE against specific SARS-CoV-2 variants (Secondary Objectives 16-17). The test-negative design and analytic plan for these analyses are detailed in Section 10 below.

Analyses will also be conducted to estimate the VE of a booster dose of Moderna COVID-19 vaccine in non-immunocompromised individuals (Secondary Objective 18) and the VE of 3 doses of Moderna COVID-19 vaccine in immunocompromised individuals (Secondary Objective 19). The cohort study design and analytic plan are detailed in Section 11 below.
Analyses will be conducted to estimate rVE, effectiveness of booster doses, effectiveness in children 6 months to 17 years of age, and effectiveness of new vaccine formulations (Section 17 Appendix 1).

6 Study Population

6.1 Eligibility Criteria

Individuals will be eligible for inclusion in the study if they meet the criteria below. Index dates are detailed in Section 6.2 below.

Inclusion criteria

- Aged ≥18 years at index date (individuals aged 6 months through 17 years are discussed in Section 17.3.3)
- KPSC member for ≥12 months prior to index date through 14 days after the index date (allowing a 31-day gap)
  Membership is necessary to follow-up individuals for COVID-19 outcomes in the EHR. In addition, prior membership allows comorbidities and other covariates to be identified and considered in analyses as potential confounders.

Exclusion criteria

- Receipt of a COVID-19 vaccine other than Moderna COVID-19 vaccine prior to or on the index date
- Receipt of 2 doses of Moderna COVID-19 vaccine <24 days apart [14] for 2-dose exposed cohort
- Receipt of any COVID-19 vaccine <14 days after the index date
- No health care utilization and no vaccination from the 2 years prior to the index date through the index date
- Occurrence of a COVID-19 outcome <14 days after the index date

6.2 Index Dates

All individuals meeting criteria in Section 6.1 will be included in analyses. Index dates will be assigned at each analysis. For vaccinated individuals, the index date for Primary Objectives 1-2 and Secondary Objectives 1-13 will be the date of receipt of the second dose of Moderna COVID-19 vaccine. For Secondary Objectives 14-15 among individuals who receive only 1 dose of Moderna COVID-19 vaccine, the index date for vaccinated individuals will be the date of receipt of the first dose of Moderna COVID-19 vaccine.

The index date for the comparison group will depend on the analysis. The comparison group will comprise a similar population meeting criteria in Section 6.1 who are unvaccinated at the index date. For the first and second interim analyses (if sample size allows), unvaccinated individuals will be randomly selected and n:1 matched to the vaccinated individuals by age (18-44 years, 45-64 years, 65-74 years, and ≥75 years), sex, and race/ethnicity (Non-Hispanic White, Non-Hispanic Black, Hispanic, Non-Hispanic Asian, and Other/Unknown). The matching ratio will depend on the sample size of the unvaccinated population, between 1:1 to 5:1. The index date for the unvaccinated match will be the same as his/her matched vaccinated counterpart.

For the subsequent interim analyses and final analysis, we expect that there will be insufficient unvaccinated individuals for 1:1 matching, as COVID-19 vaccination is expanded to the general population. If this is the case, unvaccinated individuals will be randomly selected and matched to the vaccinated individuals by the distribution (frequency) of age, sex, and race/ethnicity of vaccinated individuals. Since frequency matching is not conducted on the individual level, an index date will be assigned to unvaccinated individuals based on the distribution of calendar time of vaccinated individuals on the vaccination (index) date in each age, sex, and

race/ethnicity stratum. For example, if 5% of vaccinated individuals are vaccinated during the week of February 1, 2021, 5% of unvaccinated individuals will be assigned an index date of February 1, 2021. Hence, the distribution of index dates between the vaccinated group and the unvaccinated group will be matched. For each interim analysis during the accrual period (December 2020 to December 2021), the comparison group will be assessed and assigned with index dates independently based on all available data. See Sections 11.1, 11.2, and Section 17 Appendix 1 for updates to comparator groups, accrual periods, and analyses.

7 Measures

7.1 Exposure Definition

The primary exposure for this study (Primary Objectives 1-2, Secondary Objectives 1-13) will be receipt of 2 doses of Moderna COVID-19 vaccine received ≥24 days apart (allowing a 4-day grace period prior to the recommended interval of 28 days) during the accrual period. The exposure for Secondary Objectives 14-15 will be 1 dose of Moderna COVID-19 vaccine among individuals who received only 1 dose during the accrual period, and the exposure for Secondary Objective 9 will be receipt of 2 doses of Moderna COVID-19 vaccine during the accrual period, with either dose given concomitantly with another vaccine.

- CVX code: 207
- KPSC Immunization ID and description:
    - 124285 COVID-19, mRNA, LNP-S, PF (Moderna)
    - 124287 COVID-19 vaccine, Moderna, external administration
    - 124377 COVID-19 vaccine, mRNA, LNP-S, PF (Moderna)-50 mcg
    - 124385 COVID-19 vaccine, mRNA LNP-S, PF 6YRS-11YRS/ADULT BOOSTER, 50 mcg
    - 124386 COVID-19 vaccine, mRNA LNP-S, PF 6YRS-11YRS/ADULT BOOSTER, 50 mcg, external administration
    - 124398 COVID-19, mRNA LNP-S, PF 6MOS-5YRS, 25 mcg
    - 124399 COVID-19, mRNA LNP-S, PF 6MOS-5YRS, 25 mcg, external administration
- Manufacturer: Moderna, Inc
- Product name: Moderna COVID-19 Vaccine

7.2 Outcome Definitions

The primary outcomes for this study are:

1. SARS-CoV-2 infection will be defined as a positive molecular test or a COVID-19 diagnosis code (Primary Objective 1, Secondary Objectives 1-12, 14) (see Section 17 Appendix 1 for updates to definition)

2. Severe COVID-19 disease includes COVID-19 hospitalization (hospitalization with a SARS-CoV-2 positive test or a COVID-19 diagnosis, or a hospitalization occurring ≤7 days after a SARS-CoV-2 positive test, with chart review to confirm severe COVID-19 symptoms) and COVID-19 mortality (death during COVID-19 hospitalization) (Primary Objective 2, Secondary Objectives 13, 15)

We will ascertain the first occurrence of SARS-CoV-2 infection or severe COVID-19 disease ≥14 days after the index date.
Incident SARS-CoV-2 infections (identified through diagnosis code or positive molecular test) will be separated into symptomatic and asymptomatic SARS-CoV-2 infections. COVID-19 symptoms will be identified using a natural language processing (NLP) algorithm (currently being submitted for publication). The NLP algorithm will be applied to clinical notes before and after the incident SARS-CoV-2 infection date to search for COVID-19 related symptoms. SARS-CoV-2 infections with no symptoms identified will be considered asymptomatic cases.

Positive SARS-CoV-2 tests conducted for surveillance of asymptomatic individuals will also be used as a preliminary approach to identify asymptomatic SARS-CoV-2 infections until the NLP algorithm is finalized. The use of testing data in asymptomatic individuals will facilitate assessment of VE against asymptomatic SARS-CoV-2 infection for the first and second interim analyses, whereas the more comprehensive NLP approach will require more time to apply and will be used for subsequent analyses.

7.3 Other Variable Definitions

Other variables (Table 3) will be identified from EHR and considered in analyses when feasible and appropriate as covariates or stratification variables. The list of possible covariates may be modified for future analyses (e.g., add ages 17 years and under), as outlined in Section 17.3.3.

Table 3. List of possible covariates

	Categories
**Variables to be assessed at index date (Day 0)**	
Age at index date, years	18-44, 45-64, 65-74, 75+
Sex	Female, Male
Race/Ethnicity	Non-Hispanic White, Non-Hispanic Black, Non-Hispanic Asian, Hispanic, Other/Unknown
Month of index date	By calendar month
**Socioeconomic status**	
Medicaid	Yes, No
Neighborhood median household income	To be determined based on variable distribution
Medical center area	MC1, MC2, MC3, …
Pregnancy status	Yes (1st trimester, 2nd trimester, 3rd trimester), No
**Variables to be assessed in the two years prior to index date (-730 to -1 day, inclusive)**	
Any smoking behavior	Yes, No, Unknown
Most recent Body Mass Index (BMI) measurement	<18.5, 18.5 - <25, 25 - <30, ≥30 - <35, 35 - <40, 40 - <45, ≥45, Unknown
**Variables to be assessed in the year prior to index date (-365 to -1 day, inclusive)**	
Charlson comorbidity score [15]*	0, 1, 2+
**Autoimmune conditions**	Yes, No
Rheumatoid arthritis	Yes, No
Inflammatory bowel disease	Yes, No
Psoriasis	Yes, No
Psoriatic arthritis	Yes, No
Multiple sclerosis	Yes, No
Systemic lupus erythematosus	Yes, No
**Health care utilization**	
Number of virtual and outpatient encounters	0, 1-4, 5-10, 11+
Number of emergency encounters	0, 1, 2+

	Categories
Number of inpatient encounters	0, 1, 2+
Preventive care - with other vaccinations, screenings, and well-visits from all settings	Yes, No
**Chronic diseases**	
Kidney disease	Yes, No
Heart disease	Yes, No
Lung disease	Yes, No
Liver disease	Yes, No
Diabetes	Yes, No
**Other variables**	
KPSC physician/employee status at index date	Yes, No
Frailty index in year prior to index date (-365 to -1 day, inclusive), using method by Kim et al. [16]	To be determined based on quartiles
History of SARS-CoV-2 infection (from March 1, 2020 to index date, inclusive)	Yes, No
History of SARS-CoV-2 molecular test performed (from March 1, 2020 to index date, inclusive), regardless of result	Yes, No
Concomitant vaccine	Yes, No
**Immunocompromised status**	Yes, No
HIV/AIDS any time prior to index date	Yes, No
Leukemia, lymphoma, congenital immunodeficiencies, asplenia/hyposplenia any time prior to index date	Yes, No
Organ transplant any time prior to index date	Yes, No
Immunosuppressant medications at index date	Yes, No

* Charlson comorbidities include myocardial infarction, congestive heart failure, peripheral vascular disease, cerebrovascular disease, connective tissue disease, dementia, chronic pulmonary disease, connective tissue disease, peptic ulcer disease, diabetes mellitus, paraplegia and hemiplegia, renal disease, liver disease, cancer, metastatic solid tumor, human immunodeficiency virus (HIV), and acquired immunodeficiency syndrome (AIDS)

The covariates included in adjusted analyses will be determined by scientific relevance, association with exposure, and data availability. Specifically, we will select covariates by following these steps:

1. The distribution of covariates will be reviewed. Some categories of a categorical variable with small sample sizes may be redefined or combined into one category.
2. The association of baseline covariates with exposure will be assessed. We will use standardized difference to assess the balance of covariates between exposed and unexposed cohorts. Unlike p-values, for which magnitude is highly related to sample size, standardized difference is a unified approach to quantifying the magnitude of difference between groups regardless of sample size, where an absolute value less than 0.1 is considered a negligible difference. Potential confounders will be determined by absolute standardized difference (ASD) >0.1.

3. All potential confounders from Step 2 will be included in the analyses. Steps 1 and 2 will be repeated for the 2-dose cohort and the 1-dose cohort. Matching variables (age, sex, race/ethnicity, and index date), which are considered important risk factors, will be kept in the adjusted model for possible imbalance on matching variables due to loss to follow-up or subgroup analysis (e.g., by baseline comorbidities).

8 Statistical Analysis for Study Objectives 1-15

8.1 Analysis Plan

For Primary Objectives 1-2, interim analyses will be conducted twice in the first year, in July 2021 and October 2021 (Figure 1). The first interim analysis will include all vaccinated individuals who are accrued in the first ~3 months of the study (December 2020 to March 2021) and followed for at least 3 months after completion of the second dose. The second and third interim analyses will be performed in October 2021 and January 2022 with cumulative accrual from December 2020 to June 2021 and December 2020 to September 2021, respectively. Interim analyses will be performed every 6 months in the second and the third year. Matching of the unvaccinated comparator cohort to the vaccinated cohort will be conducted separately at each of the first 4 interim analyses, such that each analysis will have a different unvaccinated cohort. However, the unvaccinated cohort will remain fixed beginning with the 4th interim analysis through the final analysis (Primary Objectives 1-2 and Secondary Objectives 1-15), which will include all vaccinated individuals accrued in 2021, with follow-up through December 2023. No data lags are applied for the first 3 interim analyses, but a 3-month data lag is applied starting with the 4th interim analysis. The removal of the data lag for the first 3 interim analyses will permit more rapid generation of results. Without the 3-month data lag, some events reported through claims may be missed, leading to an underestimation of incidence rates. However, we expect the proportion of claims to be non-differential in the vaccinated and unvaccinated cohorts. Due to evolving public health priorities, we may replace some planned interim analyses with other analyses to address urgent public health questions. The timelines in Figure 1 and Table 6 may be shifted accordingly. See Section 11 and Section 17 Appendix 1 for updates to planned analyses.

Figure 1. Analysis Plan for Study Objectives 1-15

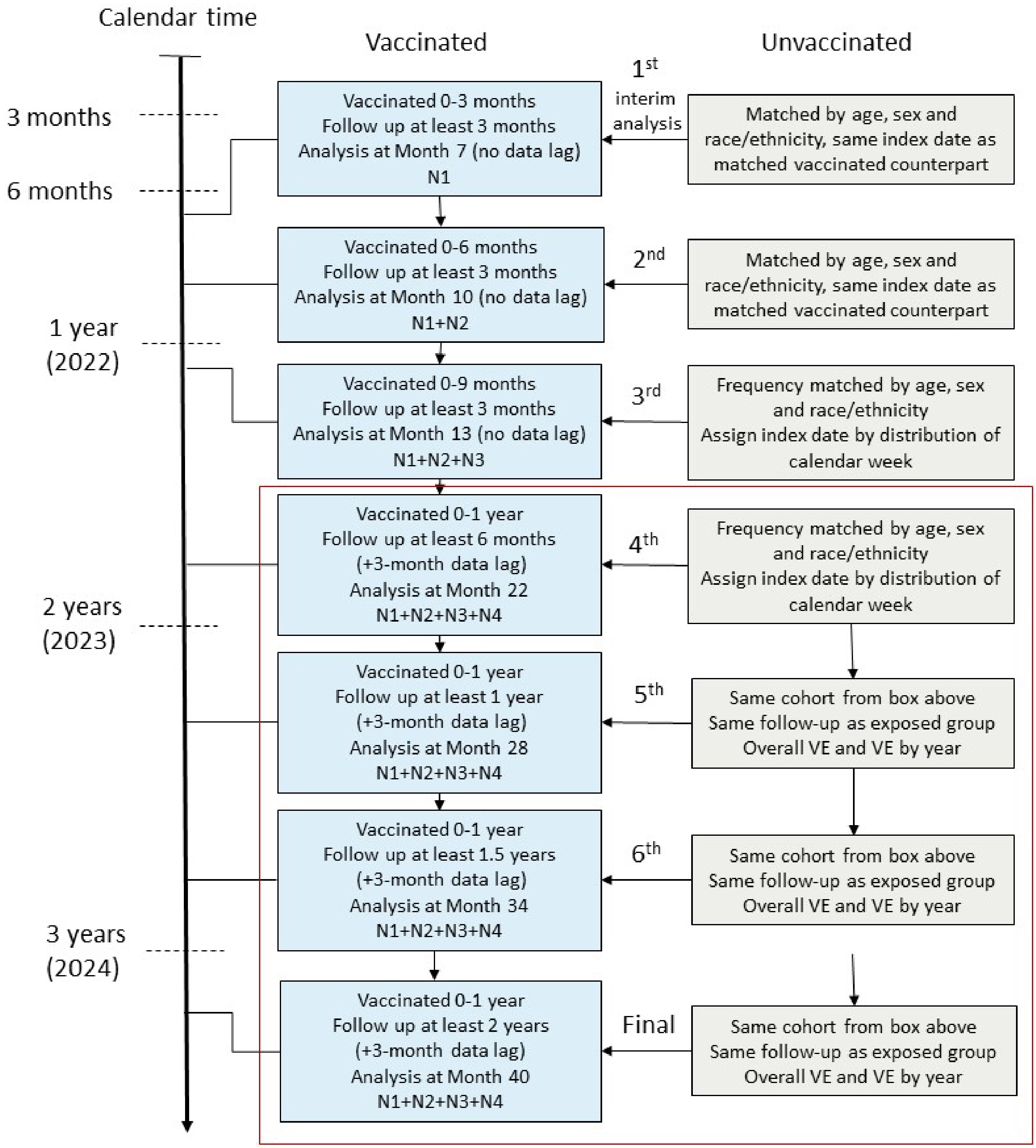

8.2 Descriptive Analysis

Descriptive attributes of vaccinated and unvaccinated cohorts will be presented as absolute numbers and percentages. We will use a χ^2 test to test for significant differences in the distribution of the categorical covariates among individuals between each of the vaccinated and unvaccinated cohorts at cohort entry. Continuous variables will be presented as the mean with standard deviation and/or median with interquartile ranges, with p-values for the two-sample t-test or Wilcoxon rank-sum test, as appropriate. Absolute standardized differences will be calculated to assess the balance of covariates.
Overall incidence rates of SARS-CoV-2 infection and of severe COVID-19 for the vaccinated and unvaccinated cohorts will be calculated by dividing the number of incident events by the total number of person-years.

8.3 Primary Analyses

The primary analyses addressing Primary Objectives 1-2 will be conducted at interim and final analyses (Figure 1).
Unadjusted hazard ratios (HR) and adjusted HR and confidence intervals (CIs) comparing SARS-CoV-2 infection or severe COVID-19 disease in vaccinated and unvaccinated individuals will be estimated by Cox proportional hazards regression models without and with adjustment for potential confounders described in Section 7.3. Unadjusted VE (%) will be calculated as (1-unadjusted HR) x 100. Adjusted VE (%) will be calculated as (1 – adjusted HR) × 100. For final analysis of primary objectives, we may also conduct propensity score analyses with inverse probability of treatment weighting (IPTW) to balance covariates across exposure groups.
We will conduct up to six interim analyses and a final analysis. The test significance level may be adjusted using an alpha spending approach without a stopping rule (similar to the Bonferroni correction, p=0.05/n, n is the total number of tests). This is a conservative approach to keep the overall Type I error below 0.05. As such, we may be calculating adjusted CIs for interim and final analyses. We will provide 95% CIs for each analysis as well.

8.4 Secondary Analyses (Secondary Objectives 1-15)

Secondary analyses will be conducted for the 4th interim and the final analysis if sample size allows. Select secondary analyses will also be conducted for the 1st interim analysis (VE by age, sex, race/ethnicity, history of COVID-19, and asymptomatic SARS-CoV-2 infection [based on individuals with a positive result from a surveillance/asymptomatic test]). If feasible and as sample size allows, analyses may also be conducted for select secondary objectives at various interim analysis timepoints based on public health needs. See Section 17 Appendix 1 for updates to analyses from the 4th interim analysis onward.
For each secondary analysis, we will examine the distribution of index dates in vaccinated individuals; we will assign index dates by stratified variables so that the frequency distribution of their index dates matches that of vaccinated individuals.
Secondary Objective 1: For Secondary Objective 1, analyses will be stratified by sex and age category (18-44 years, 45-64 years, 65-74 years, and 75+ years) at index date. For each stratum, we will calculate SARS-CoV-2 infection incidence, unadjusted and adjusted HRs and VEs, as described for the primary analysis above.
Secondary Objective 2: For Secondary Objective 2, analyses will be stratified by race/ethnicity (Non-Hispanic White, Non-Hispanic Black, Hispanic, and Non-Hispanic Asian). For each stratum, we will calculate SARS-CoV-2 infection incidence, unadjusted and adjusted HRs and VEs, as described for the primary analysis above.

Secondary Objective 3: For Secondary Objective 3, analyses will include individuals with chronic diseases (e.g., chronic kidney disease, lung disease including COPD and asthma, diabetes, identified by ICD-10 codes). For each disease, we will calculate SARS-CoV-2 infection incidence, unadjusted and adjusted HRs and VEs, as described for the primary analysis above.
Secondary Objective 4: For Secondary Objective 4, analyses will include individuals who are immunocompromised (e.g., HIV, cancer, transplant, immunosuppressive medications identified by ICD-10 codes, registries, and pharmacy data). We will calculate SARS-CoV-2 infection incidence, unadjusted and adjusted HRs and VEs, as described for the primary analysis above.
Secondary Objective 5: For Secondary Objective 5, analyses will include individuals with autoimmune conditions (e.g., rheumatoid arthritis, inflammatory bowel disease, psoriasis, psoriatic arthritis, multiple sclerosis, systemic lupus erythematosus). We will calculate SARS-CoV-2 infection incidence, unadjusted and adjusted HRs and VEs, as described for the primary analysis above.
Secondary Objective 6: For Secondary Objective 6, analyses will include individuals who are frail, identified using the method by Kim et al. [16] We will calculate SARS-CoV-2 infection incidence, unadjusted and adjusted HRs and VEs, as described for the primary analysis above.
Secondary Objective 7: For Secondary Objective 7, analyses will include pregnant women. We will calculate SARS-CoV-2 infection incidence, unadjusted and adjusted HRs and VEs, as described for the primary analysis above.
Secondary Objective 8: For Secondary Objective 8, analyses will include individuals with a history of SARS-CoV-2 infection. Incident SARS-CoV-2 infection (a COVID-19 reinfection, >90 days after the most recent prior COVID-19 diagnosis code or SARS-CoV-2 positive molecular test) will be assessed. We will calculate SARS-CoV-2 infection incidence, unadjusted and adjusted HRs and VEs, as described for the primary analysis above.
Secondary Objective 9: CDC initially recommended that COVID-19 vaccines be routinely administered alone, with a minimum interval of 14 days before or after administration with any other vaccine. In May 2021, CDC updated their recommendations to indicate that COVID-19 vaccines and other vaccines may be administered without regard to timing; this includes administration of COVID-19 vaccines and other vaccines on the same day as well as within 14 days of each other. For Secondary Objective 9, we will compare individuals who received either the first or second dose of Moderna COVID-19 vaccine concomitantly with another vaccine (i.e., on the same day) with unvaccinated individuals. We will calculate SARS-CoV-2 infection incidence and unadjusted and adjusted HRs and VEs, as described for the primary analysis above.
Secondary Objective 10: For Secondary Objective 10, we will calculate VE of 2 doses of Moderna COVID-19 vaccine against asymptomatic SARS-CoV-2 infection. We will calculate the incidence of asymptomatic SARS-CoV-2 infection and estimate unadjusted and adjusted HRs and VEs, as described for the primary analysis above.
Secondary Objective 11: For Secondary Objective 11, we will calculate VE of 2 doses of Moderna COVID-19 vaccine against symptomatic SARS-CoV-2 infection. We will calculate the incidence of symptomatic SARS-CoV-2 infection and estimate unadjusted and adjusted HRs and VEs, as described for the primary analysis above.
Secondary Objective 12: For Secondary Objective 12, we will estimate the VE against SARS-CoV-2 infection by year. We will calculate SARS-CoV-2 infection incidence by follow-up year by dividing the number of incident events occurring within that year by the number of person-years of follow-up for individuals at risk in that year. We will use time-varying Cox regression models to estimate unadjusted and adjusted HRs and VEs, for follow-up Year 1, Year 2, and Year 3 after the index date.
Secondary Objective 13: For Secondary Objective 13, we will estimate the VE against severe COVID-19 by year. We will calculate severe COVID-19 incidence, unadjusted and adjusted HRs

and VEs, as described for the Secondary Objective 12 analysis above, for follow-up Year 1, Year 2, and Year 3 after the index date.

Secondary Objective 14: For Secondary Objective 14, we will estimate the VE against SARS-CoV-2 infection among those who only received 1 dose of Moderna COVID-19 vaccine compared to unvaccinated individuals. We will calculate SARS-CoV-2 infection incidence, unadjusted and adjusted HRs and VEs, as described for the primary analysis above.

Secondary Objective 15: For Secondary Objective 15, we will estimate the VE against severe COVID-19 among those who only received 1 dose of Moderna COVID-19 vaccine compared to unvaccinated individuals. We will calculate severe COVID-19 incidence, unadjusted and adjusted HRs and VEs, as described for the primary analysis above.

9 Sample Size and Power Estimation

Power calculations depend on Moderna COVID-19 vaccine implementation, uptake, and the number of individuals meeting criteria for comparable vaccinated and unvaccinated cohorts. We estimate that a total of 80,000 individuals will receive 2 doses of Moderna COVID-19 vaccine from December 2020 through March 2021, assuming 80% series completion (first interim analysis). We estimate an incidence of 30 SARS-CoV-2 infections per 1,000 unvaccinated adult KPSC members during an average 4-month follow-up period (accounting for possible censoring). We expect to have >99.9% power to detect a VE of 70%, with various sample sizes in the unvaccinated group (Table 4), using a 2-sided test with alpha=0.007 (0.05 adjusted for 6 interim analyses and 1 final analysis; this is a conservative estimate based on the maximum number of potential analyses). The calculation was performed using SAS software package (version 9.4) PROC POWER procedure.

Table 4. Power calculations for 1st interim analysis of Primary Objective 1 for various sample sizes and vaccine effectiveness estimates

Sample Size Ratio (Vaccinated vs Unvaccinated)	Incidence rate in unvaccinated group (cases/1000 persons)	VE (%) (reduction rate)	Power
1 vs 0.5	30	90	>.999
	30	80	>.999
	30	70	>.999
1 vs 1	30	90	>.999
	30	80	>.999
	30	70	>.999
1 vs 5	30	90	>.999
	30	80	>.999
	30	70	>.999

For the first interim analysis of Primary Objective 2, we estimate an incidence of 2 COVID-19 hospitalizations and 0.3 deaths per 1,000 unvaccinated adult KPSC members during an average 4-month follow-up period. We expect to have >99.9% power to detect a 70% VE against COVID-19 hospitalizations, with various sample sizes in the unvaccinated group (Table 5).

Table 5. Power calculations for 1st interim analysis of Primary Objective 2 for various sample sizes and vaccine effectiveness estimates

Sample Size Ratio (Vaccinated vs Unvaccinated)	Incidence rate in unvaccinated group (cases/1000 persons)	VE (%) (reduction rate)	Power
1 vs 0.5	2	90	>.999
	2	80	>.999
	2	70	>.999
1 vs 1	2	90	>.999
	2	80	>.999
	2	70	>.999
1 vs 5	2	90	>.999
	2	80	>.999
	2	70	>.999
1 vs 0.5	0.3	90	0.84
	0.3	80	0.69
	0.3	70	0.51
1 vs 1	0.3	90	0.93
	0.3	80	0.81
	0.3	70	0.62
1 vs 5	0.3	90	0.99
	0.3	80	0.97
	0.3	70	0.82

Based on the current Moderna COVID-19 vaccine implementation plan and anticipated uptake, we expect to accrue 500,000 to 1,000,000 individuals receiving 2 doses of Moderna COVID-19 vaccine for analyses of primary objectives by the end of 2021.

10 Statistical Analysis Plan for Vaccine Effectiveness Against SARS-CoV-2 Variants (Secondary Objectives 16-17)

As multiple SARS-CoV-2 variants have been identified over the course of the pandemic, it is essential to identify the viral variant causing disease in this long-term effectiveness study. WGS of SARS-CoV-2 positive specimens from vaccinated and unvaccinated individuals may be performed to evaluate the following secondary objectives:

16. To assess the VE of two doses of Moderna COVID-19 vaccine against SARS-CoV-2 variants (test-negative design)
17. To assess the VE of one dose of Moderna COVID-19 vaccine against SARS-CoV-2 variants (test-negative design only)

To assess VE of 2 doses and 1 dose of Moderna COVID-19 vaccine against SARS-CoV-2 variants (Secondary Objectives 16 and 17), we aim to use a test-negative design (TND). The TND includes all sequencing data and more easily facilitates both 2-dose and 1-dose VE analyses. For specimens where SARS-CoV-2 lineage cannot be identified by WGS, additional SGTF data may be used when available along with dominant variant trends at the time of specimen collection. If feasible and as sample size allows, we may use TND to assess VE of a booster dose of Moderna COVID-19 vaccine (in non-immunocompromised individuals) and VE of a third dose of Moderna COVID-19 vaccine (in immunocompromised individuals) against SARS-CoV-2 variants. The threshold for testing individuals with symptoms suggestive of COVID-19 at KPSC does not differ by vaccination status. Variants will be selected for analyses based on prevalence in the KPSC population and scientific interest. Descriptive analyses will include the percentages of specimens from vaccinated and unvaccinated individuals sequenced with valid results, stratifying by cycle threshold (Ct) values, specimen type, and symptomatic/asymptomatic test types. We will also examine the proportions of vaccinated and unvaccinated individuals with each variant overall, by specific characteristics (e.g., age, sex, race/ethnicity, immunocompromised status, other comorbidities, history of COVID-19), COVID-19 hospitalization/hospitalized death, and month of specimen collection.
Analyses will be conducted to assess rVE against SARS-CoV-2 variants in adults ≥18 years (Section 17 Appendix 1).

10.1 Test-negative design (TND)

Inclusion criteria

- Aged ≥18 years (individuals aged 6 months through 17 years are discussed in Section 17.3.3) as of date of specimen collection for the SARS-CoV-2 test (Day 0)
- KPSC member for ≥12 months prior to Day 0 (Day -365 to Day 0, inclusive, and allowing a 31-day gap)
- Tested positive for SARS-CoV-2 by PCR at KPSC with specimen collected on or after 3/1/2021 and sent for WGS, without a history of a COVID-19 diagnosis code or SARS-CoV-2 positive molecular test in the 90 days prior to the test; or tested negative for SARS-CoV-2 by PCR with specimen collected on or after 3/1/2021 (if WGS is unavailable, SGTF data may also be used)

Exclusion criteria

- Receipt of a COVID-19 vaccine other than Moderna COVID-19 vaccine on or prior to the date of specimen collection for the SARS-CoV-2 test
- Receipt of 2 doses of Moderna COVID-19 vaccine <24 days apart

- Receipt of a Moderna COVID-19 vaccine <14 days prior to the date of specimen collection for the SARS-CoV-2 test (Day -13 to Day 0, inclusive)
- Receipt of more than 2 doses of Moderna COVID-19 vaccine on or prior to the date of specimen collection for the SARS-CoV-2 test
- Having a history of a COVID-19 diagnosis code or SARS-CoV-2 positive molecular test between 12/18/2020 and 2/28/2021, inclusive.

The exclusion criteria may be adjusted to permit evaluation of booster and third doses of the Moderna COVID-19 vaccine.

Matching

Test-positive cases: Individuals who meet the inclusion/exclusion criteria, and have a positive SARS-CoV-2 test on or after 3/1/2021 with the specimen sent for WGS will be defined as cases (if WGS is unavailable, SGTF data may also be used). For individuals who have more than one positive test after 3/1/2021, sequencing data from the first specimen during the study period will be included in analyses. The date of specimen collection for the positive test will be referred to as the index date.

Test-negative controls: Individuals who meet the inclusion/exclusion criteria, have a negative SARS-CoV-2 molecular test on or after 3/1/2021, and do not have a positive test on or after 3/1/2021 will be defined as controls. Controls will be randomly selected and matched.

Individual matching will be applied for both the 2-dose analysis and 1-dose analysis. Controls will be matched to cases by age group (18-44 years, 45-64 years, 65-74 years, and 75+ years at specimen collection date), sex, race/ethnicity (Non-Hispanic White, Non-Hispanic Black, Hispanic, Non-Hispanic Asian, and Other/Unknown), and specimen collection date (+/- 10 days). The matched date of specimen collection for the negative test will be the index date for the matched control.

Outcomes

Variants being monitored include:

- Alpha (B.1.1.7 and Q lineages)
- Beta (B.1.351 and descendent lineages)
- Gamma (P.1 and descendent lineages)
- Delta (B.1.617.2 and AY lineages)
- Epsilon (B.1.427 and B.1.429)
- Eta (B.1.525)
- Iota (B.1.526)
- Kappa (B.1.617.1)
- B.1.617.3
- Mu (B.1.621, B.1.621.1)
- Zeta (P.2)

Variants of concern (VOC) include:

- Omicron (B.1.1.529, BA.1, BA.1.1, BA.2, BA.3, BA.4 and BA.5 lineages)

Variants of interest (VOI) include:

- None as of April 2022

Groupings and naming of variants are determined based on SARS-CoV-2 Variant Classifications and Definitions (2021) by Centers for Disease Control and Prevention (CDC) as of 4/26/2022. (Available from: https://www.cdc.gov/coronavirus/2019-ncov/variants/variant-classifications.html). Sub-lineages B.1.526.1 and B.1.526.2 are added to Iota in addition to B.1.526 considering the phylogenetic finding that the B.1.526 lineage is comprised of these two closely related sub-lineages. [17] Updates may be applied at the time of analysis. For severe COVID-19 disease, a variant with specimen collection date during a hospitalization or with a hospitalization occurring ≤7 days after the specimen collection date (Day 0 to Day 7) will be identified and chart reviewed. COVID-19 hospitalization is defined as hospitalization with severe COVID-19 disease confirmed by

chart review. A variant with death during the COVID-19 hospitalization is considered a COVID-19 hospitalized death. These outcomes will only be analyzed if samples sizes are sufficient.

Exposures

For the 2-dose analysis, the exposure will be defined as receipt of the second dose of Moderna COVID-19 vaccine ≥14 days prior to the SARS-CoV-2 test date vs. not receiving a dose of any COVID-19 vaccine prior to the SARS-CoV-2 test date.

For the 1-dose analysis, the exposure will be defined as receipt of only 1 dose of Moderna COVID-19 vaccine ≥14 days prior to the SARS-CoV-2 test date vs. not receiving a dose of any COVID-19 vaccine prior to the SARS-CoV-2 test date.

Statistical Analysis

Separate analyses will be conducted using the TND for 2-dose and 1-dose VE.

For each variant, characteristics of test-positive cases and their matched test-negative controls will be described and compared. Specimens with unsuccessful sequencing may also be considered a test-positive case group for analysis. Continuous covariates such as age in years will be summarized by mean, standard deviation, median, quartiles, minimum, and maximum value, and compared using t-test or Wilcoxon rank-sum test, as appropriate; categorical covariates will be summarized by frequency and percentage and compared using chi-square test or Fisher's exact test, as appropriate. Absolute standardized difference will be calculated to assess the balance of covariates. Potential confounders will be determined based on bivariate analyses and scientific relevance. Given the small sample size and possible need to limit the number of covariates in an adjusted model, potential confounders will be determined by absolute standardized difference (ASD) >0.1 and p-value<0.1, or scientific relevance.

Conditional logistic regression will be used to estimate the odds ratio (OR) and confidence intervals (CIs) of being vaccinated among cases vs controls, without and with adjustment for potential confounders determined from bivariate analyses. VE (%) will be calculated as (1- OR) x 100.

The TND analysis will be performed at multiple timepoints. For each analysis, the test significance level may be adjusted using the Bonferroni correction (p=0.05/n tests, n is the total number of tests). As such, we may be calculating adjusted CIs for each analysis. We will provide 95% CIs for each analysis as well. If sample sizes allow, separate analyses using the same methods described above will be considered for variants associated with severe COVID-19 disease (COVID-19 hospitalization).

For selected variants (e.g., Delta variant), if sample sizes allow, separate analyses will be conducted to assess the VE of Moderna COVID-19 vaccine against SARS-CoV-2 variants by time since most recent Moderna COVID-19 vaccination using the methods similar to those described above. Matching possibly will not be maintained in the subgroup analyses. Matching variables (age, sex, and race/ethnicity) and month of specimen collection will be kept in the adjusted model.

10.2 Other considerations

Variants will be selected for analysis based on prevalence in the KPSC population and scientific interest. For a 1:5 matched TND, assuming 40% vaccination rate in test negative controls, 26 variant cases are needed to detect a VE of 0.8 with 80% statistical power.

Analyses will be performed at multiple time points. We will conduct an analysis in September 2021 including specimens collected through July 2021 (individuals with variants and matched test-negative controls). We will also conduct an analysis in August 2022 with specimens collected through June 2022 (see Section 17 Appendix 1 for updates to this planned analysis). If feasible and as sample size allows, analyses of emerging variants may also be conducted at additional timepoints based on public health needs.

11 Statistical Analysis Plan for Vaccine Effectiveness of Booster and Third Dose (Secondary Objectives 18-19)

The EUA for Moderna COVID-19 vaccine was amended on August 12, 2021 to authorize a third primary series dose (100 µg dose) at least 1 month following the second dose for individuals at least 18 years of age who have undergone solid organ transplantation or are diagnosed with conditions that are considered to have an equivalent level of immunocompromise.[5] The CDC recommended that the additional primary dose (i.e., third dose) of Moderna COVID-19 vaccine should be administered at least 28 days after completion of the initial 2-dose Moderna COVID-19 primary series in moderately or severely immunocompromised persons aged 18 years and older.[14]

On October 20, 2021, the FDA authorized use of Moderna COVID-19 vaccine as a booster dose (50 µg dose) in adults. In October 2021, CDC recommended use of the Moderna COVID-19 vaccine as a booster dose at least 6 months after completion of the primary series in certain risk groups. The EUA for Moderna COVID-19 vaccine was amended on November 19, 2021 to authorize use of the vaccine as a single booster dose in individuals 18 years of age or older, at least 6 months after completing the primary series of this vaccine (i.e., as a homologous booster dose), and to authorize use of the vaccine as a booster dose following completion of primary vaccination with another authorized or approved COVID-19 vaccine (i.e., as a heterologous booster dose) in individuals 18 years of age or older.[5] As of November 2021, the CDC expanded its recommendations for booster doses of COVID-19 vaccines. All adults ages 18 and older should receive a single COVID-19 booster dose at least 6 months after completing their mRNA primary series or at least 2 months after completing their Janssen primary vaccine dose. While any of the FDA-authorized or FDA-approved COVID-19 vaccines can be used for booster vaccination, our focus in this analysis is receipt of a Moderna booster dose following a Moderna primary series (homologous booster dose). Additional booster dose recommendations are described in Section 17 Appendix 1.

The following two secondary objectives will evaluate the VE of Moderna COVID-19 vaccine given these new recommendations:

18. To assess the VE of a booster dose of Moderna COVID-19 vaccine in preventing SARS-CoV-2 infection and severe COVID-19 disease in non-immunocompromised individuals
19. To assess the VE of 3 doses (primary series) of Moderna COVID-19 vaccine in preventing SARS-CoV-2 infection and severe COVID-19 disease in immunocompromised individuals

11.1 Booster Vaccination in Non-Immunocompromised Individuals

Overview: For Secondary Objective 18, among non-immunocompromised individuals who received a booster dose in addition to the initial 2-dose Moderna primary series, we will estimate the rVE of the booster dose against SARS-CoV-2 infection and severe COVID-19 disease compared to non-immunocompromised individuals who received the initial 2-dose Moderna primary series only. We will calculate the incidence of SARS-CoV-2 infection and severe COVID-19 disease, as well as unadjusted and adjusted HRs and rVEs as described for the primary analysis above. We will not include immunocompromised status as a covariate as it will be assessed in Secondary Objective 19.

The non-immunocompromised matched cohort will be developed in a similar way as the matched cohort assembled for the primary analyses (Section 6.1 and Section 6.2, Primary Objectives 1-2) with adults aged ≥18 years at index date. The cohort eligibility criteria will be applied based on the modified index date.

The primary exposure for Secondary Objective 18 will be receipt of the booster dose of Moderna COVID-19 vaccine given at least 6 months after receipt of the second dose during the accrual period. The outcomes will be as described in Section 7.2.

Analyses related to boosters will be conducted for Secondary Objective 18. The accrual period will start on 10/20/2021 with the last booster dose given on 12/31/2021 for the booster dose first analysis. Follow-up for this analysis will continue until 01/31/2022. No data lags will be applied. The index date will be the date of receipt of the booster Moderna COVID-19 vaccine. See Section 17 Appendix 1 for updates to additional booster analyses.

The comparator group for these analyses will consist of non-immunocompromised individuals who received only two doses of Moderna COVID-19 vaccine by the index date. Individuals will be randomly selected and n:1 matched to individuals who received the booster dose by age, sex, race/ethnicity, and the date of the second dose, if sample size allows. Individuals in the comparator group will be assigned an index date based on the date of receipt of the booster dose of their matched individual.
Covariates will be same as those in Section 7.3. Analyses will be conducted as described previously in Sections 8.2 and 8.3. Booster dose rVE may be evaluated in subgroups of interest. If feasible and as sample size allows, we may also evaluate the durability of protection, and booster dose analyses may also be conducted at additional timepoints based on public health needs.

11.2 Third Primary Dose in Immunocompromised Individuals

Overview: For Secondary Objective 19, among immunocompromised individuals, we will calculate the rVE of 3 doses of Moderna COVID-19 vaccine against SARS-CoV-2 infection and severe COVID-19 disease compared to immunocompromised individuals who received 2 doses only. We will calculate SARS-CoV-2 infection and severe COVID-19 disease incidence and unadjusted and adjusted HRs and rVEs, as described for the primary analysis above.
Immunocompromised status will be assessed as described in Section 7.3, Table 3. The immunocompromised matched cohort will be developed in a similar way as the matched cohort assembled for the primary analyses (Section 6.1 and Section 6.2, Primary Objectives 1-2) with adults aged ≥18 years at index date. The cohort eligibility criteria will be applied based on the modified index date.
The primary exposure for this objective will be receipt of 3 doses of the 3-dose primary series of Moderna COVID-19 vaccine, each received ≥24 days apart (allowing a 4-day grace period prior to the recommended interval of 28 days) during the accrual period. The outcomes will be as described in Section 7.2.
Analyses related to rVE of the 3-dose primary series will be conducted for Secondary Objective 19. The accrual period will start 8/12/2021 with the third dose given by 12/31/2021 for the 3-dose first analysis. Follow-up for the 3-dose first analysis will continue until 01/31/2022. No data lags will be applied. The index date will be the date of receipt of the third dose of Moderna COVID-19 vaccine. See Section 17 Appendix 1 for future analyses of booster doses in immunocompromised individuals.
The comparator group will be immunocompromised individuals who received two doses of the Moderna COVID-19 vaccine by the index date. Individuals in the comparator group will be randomly selected and n:1 matched to 3-dose vaccinated immunocompromised individuals by age, sex, race/ethnicity, and date of the second dose (if sample size allows). The 2-dose comparators will be assigned an index date based on the date of receipt of the third dose of their matched vaccinated individual.
Covariates will be same as those in Section 7.3. Analyses will be conducted as described previously in Sections 8.2 and 8.3. rVE of 3 doses of Moderna COVID-19 vaccine may be evaluated in subgroups of interest. If feasible and as sample size allows, we may also evaluate the durability of protection for the 3 doses in immunocompromised individuals, and analyses may also be conducted at additional timepoints based on public health needs.

12 Timelines

Table 6. Estimated Project Timelines

Description	Estimated timeline*
Contract executed	01/28/2021
KPSC to submit protocol to Moderna	02/09/2021
KPSC to obtain IRB approval	04/30/2021
KPSC to submit final Project Management Plan to Moderna	05/30/2021
KPSC to submit final Data Management and Statistical Analysis Plan to Moderna	06/16/2021
Vaccinated individuals accrual	12/18/2020-12/31/2021
End of follow-up	12/31/2023
KPSC to submit first interim analysis manuscript to Moderna (Vaccination between 12/18/2020-3/31/2021)	08/31/2021
KPSC to submit variant VE manuscript #1 to Moderna (interim results) (Specimen collection between March 2021-July 2021)	10/31/2021
KPSC to submit second interim analysis manuscript to Moderna (Vaccination between 12/18/2020-6/30/2021)	11/30/2021
KPSC to submit omicron VE manuscript to Moderna (Specimen collection between 12/6/2021–12/31/2021)	01/07/2022
KPSC to submit 3-dose VE (in immunocompromised) manuscript #1 to Moderna ¥ (Third dose between 8/12/2021–12/31/2021)	06/30/2022
KPSC to submit booster VE (in immunocompetent) manuscript #1 to Moderna ¥ (Booster dose between 10/20/2021-12/31/2021)	07/15/2022
KPSC to submit variant VE manuscript #2 to Moderna (Specimen collection between 1/1/2022-6/30/2022)	11/30/2022
KPSC to submit fourth interim analysis manuscript/report to Moderna	01/31/2023
KPSC to submit VE/rVE manuscript/report to Moderna †	05/31/2023
KPSC to submit VE/rVE manuscript/report to Moderna †	07/30/2023
KPSC to submit sixth interim analysis manuscript/report to Moderna	11/30/2023
KPSC to submit end-of-study (final) analysis report tables to Moderna	09/30/2024
KPSC submission of Final Report to Moderna	03/31/2025
KPSC to submit final manuscript to Moderna	6/30/2025

*Analysis plans and vaccination accrual dates are subject to change based on public health needs. Timelines are approximate.
¥Interim analysis 3 in the original protocol (Figure 1) has been replaced by the booster in immunocompetent and 3-dose in immunocompromised analyses.
†Interim analysis 5 in the original protocol (Figure 1) has been replaced by VE/rVE analyses based on public health needs and updated vaccine recommendations.

13 Data Management

Data management activities will be performed by KPSC. The KPSC EHR will be the data source for extracting information on exposures, outcomes, and covariates. KPSC will develop the study datasets, maintain documentation, and perform data quality checks.

Programmers will extract data per protocol and reference documentation for KPSC EHR databases. Study cohorts and outcomes will be double programmed by two programmers independently. Programmers will conduct data quality checks which include data integrity control and program review. Data integrity control will include checks such as sample size, duplications, formatting, etc. All decisions made during data extraction and data quality checks will be documented.

14 Limitations

This study has several potential limitations. Misclassification of the exposure is possible if COVID-19 vaccines were received outside of the KPSC system. However, external COVID-19 vaccination can be captured in the KPSC EHR data by manual entry and electronic updates from the California Immunization Registry (CAIR) (for which KPSC has a proactive mechanism in place for obtaining regular updates from CAIR). Misclassification of COVID-19 outcomes is also possible due to imperfect capture and sensitivity of SARS-CoV-2 molecular diagnostic tests. For variant VE analyses (Secondary Objectives 16-17), sequencing may be unsuccessful if viral loads are low, which may be more common among vaccinated vs. unvaccinated individuals. However, samples with unsuccessful sequencing may also be considered a test-positive case group for analysis. For patients with COVID-19 symptoms, sensitivity of molecular diagnostic tests is generally high (>90%). Efforts are made to ask patients about positive tests conducted outside of KPSC and to document in the EHR with internal diagnosis codes. In addition, we will be unable to capture some potential confounders, such as occupational risk exposures, behavioral factors (e.g., masking, distancing, handwashing), and residential factors (e.g., congregate settings) that impact an individual's risk for COVID-19. Thus, despite all efforts to ensure comparability, there may be some unmeasured differences between vaccinated and unvaccinated cohorts. Some secondary analyses may be underpowered; their meaningfulness will depend on uptake of Moderna COVID-19 vaccine in these patient populations.

15 Human Subjects Protection

The study will be reviewed and approved by the KPSC Institutional Review Board (IRB). All study staff with access to protected health information are trained in procedures to protect the confidentiality of subject data. We will obtain a waiver of informed consent as this is an observational study of authorized and recommended Moderna COVID-19 vaccine administered in the course of routine clinical care.

The HIPAA Privacy Rule governs the use and disclosure of personally identifiable information (protected health information; PHI) from covered entities. Throughout the course of this study, no PHI will be disclosed, however it will be accessed through the EHR. This information will only be accessed by those authorized to do so and will not be shared with Moderna or anyone outside of the KPSC study team. As this access presents no more than minimal risk to individuals and the research could not be practically done if required to obtain written authorization for usage, we will obtain a waiver for written HIPAA authorization for research involving use of the EHR.

16 References

1. World Health Organization. *Coronavirus disease (COVID-19) Weekly Epidemiological Update*. 2021 January 19; Available from: www.who.int/publications/m/item/weekly-epidemiological-update---19-january-2021.
2. Mascola, J.R., B.S. Graham, and A.S. Fauci, *SARS-CoV-2 Viral Variants-Tackling a Moving Target.* JAMA, 2021. **325**(13): p. 1261-1262.
3. Moore, J.P. and P.A. Offit, *SARS-CoV-2 Vaccines and the Growing Threat of Viral Variants.* JAMA, 2021. **325**(9): p. 821-822.
4. Rubin, R., *COVID-19 Vaccines vs Variants-Determining How Much Immunity Is Enough.* JAMA, 2021. **325**(13): p. 1241-1243.
5. US Food and Drug Administration. *Moderna COVID-19 Vaccine EUA Letter of Authorization*. 2020; Available from: www.fda.gov/media/144636/download.
6. Baden, L.R., et al., *Efficacy and Safety of the mRNA-1273 SARS-CoV-2 Vaccine.* N Engl J Med, 2020.
7. Shang, J., et al., *Cell entry mechanisms of SARS-CoV-2.* Proc Natl Acad Sci U S A, 2020. **117**(21): p. 11727-11734.
8. Anderson, E.J., et al., *Safety and Immunogenicity of SARS-CoV-2 mRNA-1273 Vaccine in Older Adults.* N Engl J Med, 2020. **383**(25): p. 2427-2438.
9. Jackson, L.A., et al., *An mRNA Vaccine against SARS-CoV-2 - Preliminary Report.* N Engl J Med, 2020. **383**(20): p. 1920-1931.
10. US Food and Drug Administration. *Approval Letter - SPIKEVAX*. 2022 [cited 2022 June 24]; Available from: https://www.fda.gov/media/155815/download.
11. US Food and Drug Administration. *Coronavirus (COVID-19) Update: FDA Takes Key Action by Approving Second COVID-19 Vaccine*. 2022 [cited 2022 June 24]; Available from: https://www.fda.gov/news-events/press-announcements/coronavirus-covid-19-update-fda-takes-key-action-approving-second-covid-19-vaccine.
12. US Food and Drug Administration. *Coronavirus (COVID-19) Update: FDA Authorizes Moderna and Pfizer-BioNTech COVID-19 Vaccines for Children Down to 6 Months of Age*. 2022 [cited 2022 June 24]; Available from: https://www.fda.gov/news-events/press-announcements/coronavirus-covid-19-update-fda-authorizes-moderna-and-pfizer-biontech-covid-19-vaccines-children.
13. Koebnick, C., et al., *Sociodemographic characteristics of members of a large, integrated health care system: comparison with US Census Bureau data.* Perm J, 2012. **16**(3): p. 37-41.
14. US Centers of Disease Control and Prevention. *Interim Clinical Considerations for Use of COVID-19 Vaccines Currently Approved or Authorized in the United States*. 2021 November 29; Available from: https://www.cdc.gov/vaccines/covid-19/clinical-considerations/covid-19-vaccines-us.html.
15. Quan, H., et al., *Coding algorithms for defining comorbidities in ICD-9-CM and ICD-10 administrative data.* Med Care, 2005. **43**(11): p. 1130-9.
16. Kim, D.H., et al., *Measuring Frailty in Medicare Data: Development and Validation of a Claims-Based Frailty Index.* J Gerontol A Biol Sci Med Sci, 2018. **73**(7): p. 980-987.
17. Annavajhala, M.K., et al., *Emergence and Expansion of the SARS-CoV-2 Variant B.1.526 Identified in New York.* medRxiv, 2021.
18. Wallace, M., et al., *The Advisory Committee on Immunization Practices' Recommendation for Use of Moderna COVID-19 Vaccine in Adults Aged >/=18 Years and Considerations for Extended Intervals for Administration of Primary Series Doses of mRNA COVID-19 Vaccines - United States, February 2022.* MMWR Morb Mortal Wkly Rep, 2022. **71**(11): p. 416-421.
19. US Centers of Disease Control and Prevention. *COVID-19 vaccination guidance for people who are moderately or severely immunocompromised*. Interim Clinical Considerations for Use of COVID-19 Vaccines Currently Approved or Authorized in the United States 2022 [cited 2022 June 24]; Available from: https://www.cdc.gov/vaccines/covid-19/clinical-considerations/interim-considerations-us.html#immunocompromised.
20. US Centers of Disease Control and Prevention. *COVID-19 vaccination guidance for people who are not moderately or severely immunocompromised*. Interim Clinical Considerations for Use of COVID-19 Vaccines Currently Approved or Authorized in the United States 2022 [cited 2022 June 24]; Available

from: https://www.cdc.gov/vaccines/covid-19/clinical-considerations/interim-considerations-us.html#not-immunocompromised.

21. US Centers of Disease Control and Prevention. *ACIP Update to the Evidence to Recommendations for a 2nd COVID-19 Booster Dose in Adults Ages 50 Years and Older and Immunocompromised Individuals*. 2022 [cited 2022 June 24]; Available from: https://www.cdc.gov/vaccines/acip/recs/grade/covid-19-second-booster-dose-etr.html.

17 Appendix 1 - Protocol Amendment 4 (version 5.0)

17.1 Updates to COVID-19 Vaccination

17.1.1 Primary Series

In February 2022, the CDC recommended a 4-8-week interval between the 2-dose primary series of mRNA vaccines for immunocompetent individuals, including males aged 12-39 years due to the small risk of myocarditis associated with mRNA COVID-19 vaccines. [18]
For moderately or severely immunocompromised individuals aged 6 months and older, the CDC recommended a shorter interval of 4 weeks between the first and second dose, and a third dose ≥4 weeks after, as part of the 3-dose primary series.[19]

17.1.2 Booster Dose

In March 2022, the CDC recommended a second booster dose ≥4 months after the first booster dose, which is recommended ≥5 months after the final dose in the 2-dose primary series, in immunocompetent adults aged ≥50 years. [20, 21]
In moderately or severely immunocompromised individuals, the CDC recommended a shorter interval (3 months) between the completion of the 3-dose primary series and the first booster dose (fourth dose), and recommended a second booster (fifth dose) ≥4 months after the first booster dose. [19]

17.2 Modification to 4th Interim Analysis

Background: We will conduct the 4th interim analysis in immunocompetent individuals aged ≥50 years. We will evaluate the rVE of a second booster dose (4th dose vs. 3rd dose). We will use 3-dose vaccinated immunocompetent individuals as the comparator group instead of unvaccinated immunocompetent individuals. As more vaccinations are administered, individual behaviors likely become increasingly different between the unvaccinated and vaccinated individuals; factors associated with individual behaviors impacting care-seeking behaviors and the risk of COVID-19 are difficult to measure and control for. Therefore, we will use 3-dose vaccinated individuals as the comparator group as opposed to unvaccinated individuals to minimize such differences as well as other potential unmeasured confounders. In addition, with continued expansion of COVID-19 vaccination recommendations, the number of unvaccinated individuals to match with vaccinated individuals continues to decrease, making matching increasingly difficult; using 3-dose vaccinated individuals as the comparator group will alleviate this difficulty.
Study Design: Individuals who received 3 doses of Moderna COVID-19 vaccine will be randomly selected and n:1 matched to individuals who received 4 doses by age, sex, race/ethnicity, and third dose date (if sample size allows) and will be assigned an index date based on the fourth dose vaccination date of their matched individual.
Study Population: Immunocompetent individuals aged ≥50 years who also meet the eligibility criteria.
Accrual and Follow-up Period:

- First booster doses in immunocompetent individuals aged ≥50 years will be accrued between 10/20/2021 and 03/31/2022
- Second booster doses in immunocompetent individuals aged ≥50 years will be accrued between 03/29/2022 and 07/30/2022, with second booster dose date ≥4 months after the first booster dose date
- Follow-up through 08/31/2022

Statistical Analysis Plan:

- Of the Secondary Objectives 1-15 originally planned, Secondary Objectives 4 (immunocompromised), 7 (pregnant women), 9 (concomitant vaccination), 14 (1-dose VE against infection), and 15 (1-dose VE against severe COVID-19) will not be assessed in the 4th interim analysis. Other Secondary Objectives will be evaluated if sample size allows.

17.3 Study Design Modifications

17.3.1 SARS-CoV-2 Infection Definition

For future analyses, due to an increase in rapid antigen testing, the SARS-CoV-2 infection definition will include a positive antigen test result as well as a positive molecular test or a COVID-19 diagnosis code. This SARS-CoV-2 infection definition applies to the SARS-CoV-2 infection outcome, the covariate “history of SARS-CoV-2 infection”, and inclusion/exclusion criteria involving SARS-CoV-2 infection. The covariate “history of SARS-CoV-2 molecular test performed, regardless of result” will remain unchanged. Rapid antigen test results will not be applied for Secondary Objectives 16 and 17 (variant VE) because variant identification from WGS and SGTF data relies on molecular testing.

17.3.2 Relative Vaccine Effectiveness

For individuals aged 18 years and older, we may evaluate rVE in future analyses (variant VE #2 and 4th interim analysis onward) based on public health needs.

17.3.3 Vaccine Effectiveness Among Children Ages 6 months Through 17 Years

On June 17, 2022, the FDA amended the Moderna COVID-19 vaccine EUA to authorize vaccination to younger age groups (individuals aged 6 months through 17 years). On June 18, 2022, the CDC recommended the vaccine to be administered as a 2-dose primary series, 4-8 weeks apart in individuals aged 6 months to 5 years (25 µg dose). On June 23, 2022, the CDC further recommended the vaccine to be administered as a 2-dose primary series, 4-8 weeks apart, in individuals aged 6-11 years (50 µg dose) and 12-17 years (100 µg dose). [20]

Given these new recommendations, we will evaluate the VE of Moderna COVID-19 vaccine among those aged 6 months through 17 years.

We will calculate the incidence of SARS-CoV-2 infection and severe COVID-19 disease, as well as unadjusted and adjusted HRs and VEs as described for the primary analysis above.

The unvaccinated matched cohort will be developed in a similar way as the matched cohort assembled for the primary analyses (Section 6.1 and Section 6.2, Primary Objectives 1-2) with individuals aged 6 months through 17 years at index date. The cohort eligibility criteria will be applied based on the index date.

The primary exposure will be receipt of the second dose of Moderna COVID-19 vaccine given 4-8 weeks after receipt of the first dose during the accrual period. The outcomes will be as described in Section 7.2 and Section 17.3.1. The index date will be the date of receipt of the second dose of the Moderna COVID-19 vaccine.

The comparator group for these analyses will consist of unvaccinated individuals who did not receive any COVID-19 vaccine by the index date. Individuals will be randomly selected and n:1 matched to individuals who received the second dose by age, sex, and race/ethnicity, if sample size allows. Individuals in the comparator group will be assigned an index date based on the date of receipt of the second dose of their matched individual.

Covariates will be the same as those in Section 7.3 with potential modifications to reflect characteristics of individuals aged 6 months through 17 years.

Analyses will be conducted as described previously in Sections 8.2 and 8.3. VE may be evaluated in subgroups of interest. If feasible and as sample size allows, we may also evaluate the durability of protection, and analyses may also be conducted at additional timepoints based on public health needs. As recommendations change in this group, we may also evaluate rVE against SARS-CoV-2 infection and severe COVID-19 disease in booster-dose/3-dose primary series vaccinated individuals versus 2-dose vaccinated individuals.

17.3.4 Vaccine Effectiveness Against SARS-CoV-2 Variants

We will conduct an analysis of rVE of ≥3 doses vs. 2 doses of Moderna COVID-19 vaccine in August 2022 with specimens collected through June 2022.

17.3.5 Booster doses in Immunocompromised and Non-Immunocompromised Individuals

We may evaluate rVE of first, second, and any additional booster doses in immunocompromised and non-immunocompromised individuals.

17.3.6 New or Updated mRNA-1273 Formulations

We may evaluate the VE of the updated bivalent vaccine designed to include the Omicron variant (e.g., mRNA-1273.214, mRNA-1273.222) or other updated formulations in future analyses.
