# Supplementary material for: Effectiveness of mRNA-1273 vaccination against SARS-CoV-2 omicron subvariants BA.1, BA.2, BA.2.12.1, BA.4, and BA.5
Source: Nat Commun. 2023 Jan 12;14:189. doi: 10.1038/s41467-023-35815-7 (PMC9836332; doi:10.1038/s41467-023-35815-7)
Supplement: Supplementary file 2 — Reporting Summary [file 41467_2023_35815_MOESM2_ESM.pdf]

nature portfolio

Corresponding author(s): Hung Fu Tseng

Last updated by author(s): Dec 15, 2022

Reporting Summary

Nature Portfolio wishes to improve the reproducibility of the work that we publish. This form provides structure for consistency and transparency in reporting. For further information on Nature Portfolio policies, see our Editorial Policies and the Editorial Policy Checklist.

Statistics

For all statistical analyses, confirm that the following items are present in the figure legend, table legend, main text, or Methods section.

n/a	Confirmed	
☐	☒	The exact sample size (*n*) for each experimental group/condition, given as a discrete number and unit of measurement
☒	☐	A statement on whether measurements were taken from distinct samples or whether the same sample was measured repeatedly
☐	☒	The statistical test(s) used AND whether they are one- or two-sided<br>*Only common tests should be described solely by name; describe more complex techniques in the Methods section.*
☐	☒	A description of all covariates tested
☐	☒	A description of any assumptions or corrections, such as tests of normality and adjustment for multiple comparisons
☐	☒	A full description of the statistical parameters including central tendency (e.g. means) or other basic estimates (e.g. regression coefficient) AND variation (e.g. standard deviation) or associated estimates of uncertainty (e.g. confidence intervals)
☐	☒	For null hypothesis testing, the test statistic (e.g. *F*, *t*, *r*) with confidence intervals, effect sizes, degrees of freedom and *P* value noted<br>*Give P values as exact values whenever suitable.*
☒	☐	For Bayesian analysis, information on the choice of priors and Markov chain Monte Carlo settings
☒	☐	For hierarchical and complex designs, identification of the appropriate level for tests and full reporting of outcomes
☒	☐	Estimates of effect sizes (e.g. Cohen's *d*, Pearson's *r*), indicating how they were calculated

Our web collection on statistics for biologists contains articles on many of the points above.

Software and code

Policy information about availability of computer code

Data collection	SAS v9.4
Data analysis	SAS v9.4

For manuscripts utilizing custom algorithms or software that are central to the research but not yet described in published literature, software must be made available to editors and reviewers. We strongly encourage code deposition in a community repository (e.g. GitHub). See the Nature Portfolio guidelines for submitting code & software for further information.

Data

Policy information about availability of data

All manuscripts must include a data availability statement. This statement should provide the following information, where applicable:

- Accession codes, unique identifiers, or web links for publicly available datasets
- A description of any restrictions on data availability
- For clinical datasets or third party data, please ensure that the statement adheres to our policy

Individual-level data reported in this study involving human research participants are not publicly shared due to potentially identifying or sensitive patient information. Upon request to corresponding author [HFT], and subject to review and approval of an analysis proposal, KPSC may provide the deidentified aggregate-level data that support the findings of this study within 6 months. Anonymized data (deidentified data including participant data as applicable) that support the findings of this study may be made available from the investigative team in the following conditions: (1) agreement to collaborate with the study team on all

publications, (2) provision of external funding for administrative and investigator time necessary for this collaboration, (3) demonstration that the external investigative team is qualified and has documented evidence of training for human subjects protections, and (4) agreement to abide by the terms outlined in data use agreements between institutions.

Human research participants

Policy information about studies involving human research participants and Sex and Gender in Research.

Reporting on sex and gender	Sex of the participants was considered in the study design; data on the sex of participants was collected through self-report measures, and overall sex distribution was reported in the study. Sex- and gender-based analyses were not performed as results were not expected to vary by sex or gender. Additionally, such analyses would have further limited the sample size.
Population characteristics	Individuals were aged ≥18 years, and had ≥12 months of KPSC membership before the specimen collection date (for accurate ascertainment of exposure status and covariates). Randomly sampled controls were 3:1 matched to cases participants by age (18–44 years, 45–64 years, 65–74 years and ≥75 years), sex, race/ethnicity (non-Hispanic White, non-Hispanic Black, Hispanic, non-Hispanic Asian and other/unknown) and specimen collection date (±10 days).
Recruitment	Participants included those who had ≥12 months of KPSC membership before the specimen collection date. Participants data were extracted from the KPSC integrated health care system. Cases included individuals individuals with positive SARS-CoV-2 RT-PCR tests from specimens collected between 1/1/2022 and 6/30/2022 that were sent for WGS and controls that were identified from those with only negative SARS-CoV-2 RT-PCR tests during the same period. Vaccination is a self-selection exposure affected by a variety of social and personal factors which may lead to self-selection bias.
Ethics oversight	The study was approved by the KPSC Institutional Review Board, which waived requirements for written informed consent and written Health Insurance Portability and Accountability Act authorization, as the use of EHRs for this observational study involved minimal risk.

Note that full information on the approval of the study protocol must also be provided in the manuscript.

Field-specific reporting

Please select the one below that is the best fit for your research. If you are not sure, read the appropriate sections before making your selection.

☒ Life sciences ☐ Behavioural & social sciences ☐ Ecological, evolutionary & environmental sciences

For a reference copy of the document with all sections, see nature.com/documents/nr-reporting-summary-flat.pdf

Life sciences study design

All studies must disclose on these points even when the disclosure is negative.

Sample size	We used a test-negative case-control design to assess the effectiveness of 3 and 4 doses of mRNA-1273 against SARS-CoV-2 Omicron subvariants. Cases were identified from individuals with positive SARS-CoV-2 RT-PCR tests from specimens collected between 1/1/2022 and 6/30/2022 that were sent for WGS and controls that were identified from those with only negative SARS-CoV-2 RT-PCR tests during the same period. Individuals were included if they were aged ≥18 years and had ≥12 months of KPSC membership before the specimen collection date (necessary for accurate ascertainment of exposure status and covariates).
Data exclusions	Individuals were excluded if they had a history of SARS-CoV-2 infection in the 90 days prior to specimen collection date, received any dose of mRNA-1273 <14 days before the specimen collection date, received any 2 doses of mRNA-1273 <24 days apart or >4 doses of mRNA-1273 before the specimen collection date, or received a COVID-19 vaccine other than mRNA-1273.
Replication	Results can be replicated with de-identified data (including participant data as applicable) upon approval of an analysis proposal and a signed data access agreement.
Randomization	Randomization is not applicable because this is a real-world retrospective observational study for which there was no intervention randomization; this was not a randomized control trial.
Blinding	Blinding was not required because this is a real-world observational study in which exposure (vaccine) was given as part of routine clinical practice; this was not a blinded trial.

Reporting for specific materials, systems and methods

We require information from authors about some types of materials, experimental systems and methods used in many studies. Here, indicate whether each material, system or method listed is relevant to your study. If you are not sure if a list item applies to your research, read the appropriate section before selecting a response.

Materials & experimental systems

n/a	Involved in the study
☒	☐ Antibodies
☒	☐ Eukaryotic cell lines
☒	☐ Palaeontology and archaeology
☒	☐ Animals and other organisms
☒	☐ Clinical data
☒	☐ Dual use research of concern

Methods

n/a	Involved in the study
☒	☐ ChIP-seq
☒	☐ Flow cytometry
☒	☐ MRI-based neuroimaging
